# Catalytic enantioselective synthesis of 1,4-dihydropyridines *via* the addition of C(1)-ammonium enolates to pyridinium salts†

**DOI:** 10.1039/d1sc03860e

**Published:** 2021-08-06

**Authors:** Calum McLaughlin, Jacqueline Bitai, Lydia J. Barber, Alexandra M. Z. Slawin, Andrew D. Smith

**Affiliations:** EaStCHEM, School of Chemistry, University of St Andrews St Andrews Fife KY16 9ST UK ads10@st-andrews.ac.uk

## Abstract

The regio- and stereoselective addition of C(1)-ammonium enolates – generated *in situ* from aryl esters and the isothiourea catalyst (*R*)-BTM – to pyridinium salts bearing an electron withdrawing substituent in the 3-position allows the synthesis of a range of enantioenriched 1,4-dihydropyridines. This represents the first organocatalytic approach to pyridine dearomatisation using pronucleophiles at the carboxylic acid oxidation level. Optimisation studies revealed a significant solvent dependency upon product enantioselectivity, with only toluene providing significant asymmetric induction. Using DABCO as a base also proved beneficial for product enantioselectivity, while investigations into the nature of the counterion showed that co-ordinating bromide or chloride substrates led to higher product er than the corresponding tetrafluoroborate or hexafluorophosphate. The scope and limitations of this process are developed, with enantioselective addition to 3-cyano- or 3-sulfonylpyridinium salts giving the corresponding 1,4-dihydropyridines (15 examples, up to 95 : 5 dr and 98 : 2 er).

## Abstract 2

The regio- and stereoselective addition of C(1)-ammonium enolates – generated *in situ* from aryl esters and the isothiourea catalyst (*R*)-BTM – to pyridinium salts allows the synthesis of a range of enantioenriched 1,4-dihydropyridines.

## Graphical abstract

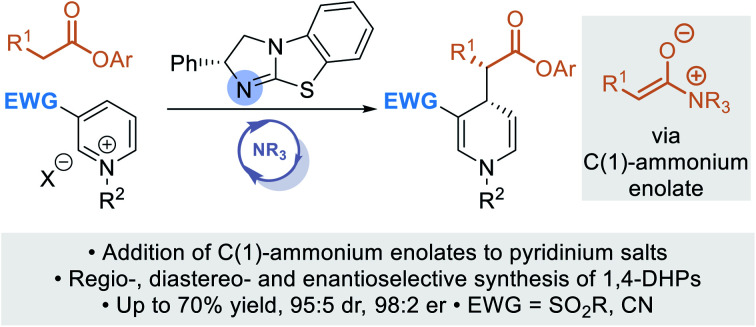

## 1. Introduction

**Scheme 1:**
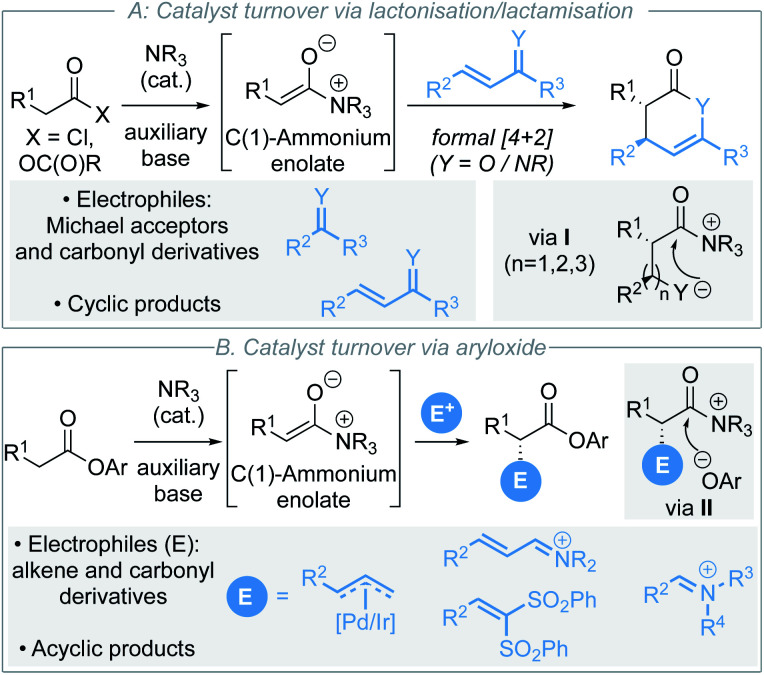
Strategies for isothiourea catalysis *via* C(1)-ammonium enolates.

C(1)-Ammonium enolates,^1^ derived from chiral tertiary amine Lewis base catalysts,^2^ have emerged as synthetically powerful catalytically-generated intermediates for enantioselective C–C bond formation. Traditionally, reaction of C(1)-ammonium enolates with electrophiles such as Michael acceptors and carbonyl derivatives (ketones, imines) in formal [4+2]^3^ and [2+2]^4^ cycloadditions has enabled a wide variety of enantioenriched heterocycles to be accessed (Scheme 1A).^5^ The electrophile is generally required to contain a latent nucleophilic site for catalyst turnover to be achieved through intramolecular cyclisation (usually lactonisation/lactamisation *via* I) to give formal cycloaddition products. Although powerful in concept, this approach also represents a fundamental limitation in these processes where initially cyclic products are formed. Recent work has focused on using electron-deficient aryl esters as C(1)-ammonium enolate precursors in isothiourea catalysis, in which an alternative catalyst turnover pathway can be accessed.^6^ In this case, the aryloxide anion, released upon *N*-acylation of the Lewis base, can react with the post-reaction acyl ammonium ion (Scheme 1B). This approach presents a strategy for the formation of acyclic α-functionalised products at the carboxylic acid oxidation level, significantly broadening the potential applicability of C(1)-ammonium enolates in enantioselective catalysis. Despite these advances, to date electrophilic partners have been limited to derivatives of alkenes (metal allyl complexes, Michael acceptors)^7^ and carbonyl derivatives (iminium ions).^8^ More appealing would be the expansion of this approach to compatible electrophiles that would allow direct installation of desirable functionality such as heterocycles.

**Scheme 2:**
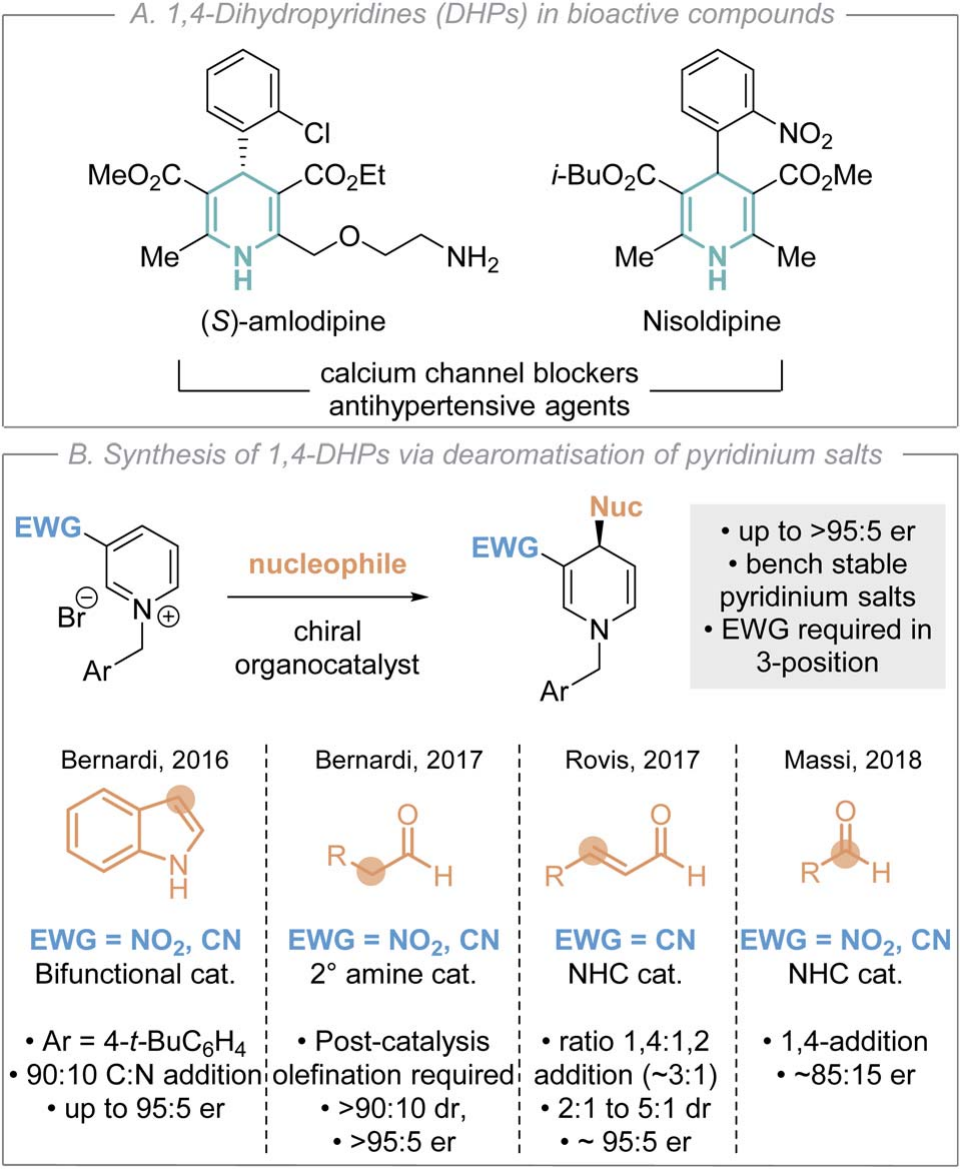
1,4-Dihydropyridines and previous organocatalytic enantioselective routes to them (EWG = electron-withdrawing group).

In this context, nitrogen-containing heterocycles are an important structural motif prevalent in a diverse range of biologically relevant compounds including pharmaceuticals,^9^ agrochemicals,^10^ natural products and dyes.^11^ Their importance is readily quantified as 59% of US FDA approved small-molecule drugs contain a nitrogen heterocycle,^12^ with these heterocycles commonly incorporated into molecules to improve physicochemical properties. 1,4-Dihydropyridines (DHPs) are a privileged class of heterocycle found in pharmaceuticals as potent calcium channel blockers for the treatment of hypertension (Scheme 2A).^13^ Although a less common fragment in terms of total number of commercial medicines (10 small-molecule drugs), incorporation of 1,4-DHP units is of increasing interest for structural diversity in the treatment of cardiorenal diseases, cancer and asthma.^14^ Additionally, 1,4-DHPs can serve as versatile intermediates in contemporary organic synthesis, such as being precursors to pyridines and piperidines.^15^ As a consequence, direct and mild methods for the efficient preparation of DHPs are highly sought after within the synthetic community. For example, Glorius and co-workers have recently developed a selective pyridine dearomatisation approach to achiral 1,4-DHPs through reduction with amine borane.^16^

Nucleophilic dearomatisation of electron deficient pyridines with carbon nucleophiles is a powerful method for the enantioselective synthesis of DHPs.^17^ Although early methods utilised stoichiometric chiral auxiliaries^18^ and preformed nucleophiles^19^ to impart stereoselectivity, recent advances have focused on the development of catalytic enantioselective protocols. Transition metal-catalysed dearomatisation reactions of pyridines^20^ have been achieved using various nucleophiles (cyanide,^20*a*^ alkyne,^20*b*,*c*,*h*^ alkyl/aryl zinc)^20*d*,*f*,*j*^ however these typically yield the C(2)- or C(6)-addition products. In recent years, a number of effective organocatalytic strategies for pyridinium dearomatisation have been demonstrated (Scheme 2B).

In 2015, Mancheño and co-workers disclosed the seminal organocatalytic enantioselective dearomatisation of *in situ* generated *N*-acyl pyridinium intermediates using silyl ketene acetal nucleophiles by employing an anion-binding catalyst to give enantioenriched 1,2-DHPs.^21^ As alternatives to these *N*-acyl derivatives, *N*-alkyl pyridinium salts are bench stable solids that can be conveniently prepared in one step from the corresponding, widely available, pyridine precursor and alkylating agent. The addition of various nucleophiles to *N*-alkyl pyridinium salts for the enantioselective synthesis of 1,4-DHPs has also been reported using organocatalytic methods (Scheme 2B). In each case, the incorporation of an electron-withdrawing nitro, nitrile or acyl group in the 3-position is required to activate the pyridinium salt, rendering it more electrophilic for nucleophilic addition. This approach was first demonstrated by Bernardi, Fochi and co-workers.^22^ They showed that a bifunctional H-bond donor could promote the asymmetric addition of indole to generate 1,4-DHPs, giving a 90 : 10 mixture of C-addition (95 : 5 er) : N-addition (50 : 50 er) products in excellent yield, requiring stoichiometric proton sponge as an additive, toluene as solvent and an *N*-4-*tert*-butylbenzyl substituent for optimal enantioselectivity. Further work from Bernardi, Fochi and co-workers used secondary amine catalysts to promote the enantioselective addition of aldehydes to *N*-alkyl pyridiniums *via in situ* generated enamines in toluene.^23^ Subsequent Wittig-olefination was used to selectively generate the isolable 1,4-DHP products in generally good yields, >90 : 10 dr and >95 : 5 er. Related manuscripts from the groups of Rovis and Massi demonstrated two alternative NHC-catalysed dearomatisation processes. Rovis and co-workers showed that homoenolates generated by NHCs from enals could be used, generating preferentially 1,4-DHPs (ratio 1,4- to 1,2-products typically ∼3 : 1) with moderate to good diastereocontrol (2 : 1 to 5 : 1) and good enantiocontrol (typically ∼95 : 5 er).^24^ Notably an acidic additive was necessary to prevent the formation of an off-cycle catalyst-pyridinium adduct. Massi demonstrated an alternative approach, showing that NHCs could promote the addition of aliphatic aldehydes to *N*-alkyl pyridiniums through the formation of an *in situ* generated Breslow intermediate, generating exclusively 1,4-DHPs with good yields and enantioselectivity (typically ∼85 : 15 er).^25^

**Scheme 3:**
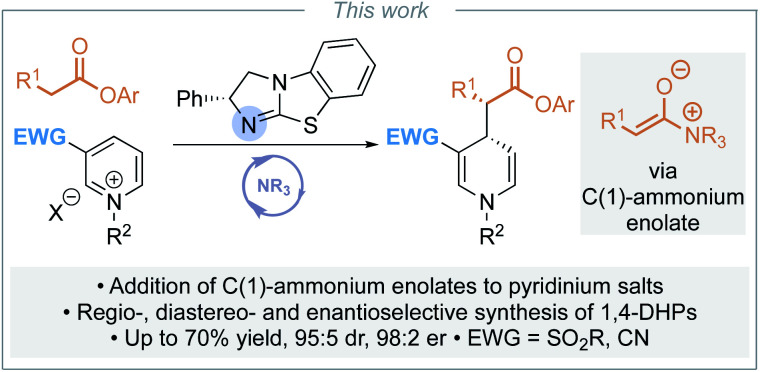
This work: enantioselective synthesis of 1,4-dihydropyridines.

Although a range of enantioselective organocatalytic approaches to dearomatisation of *N*-alkyl pyridiniums has been developed, to date none use pronucleophiles at the carboxylic acid oxidation level. To address this limitation, we envisaged addition of a C(1)-ammonium enolate intermediate, generated *in situ* from the corresponding aryl ester, to pyridinium salts for the enantioselective synthesis of α-functionalised ester substituted 1,4-DHPs (Scheme 3). The key challenges in the development of this process would be the compatibility of the reagents (nucleophilic catalyst and aryloxide with pyridinium electrophile), achieving sufficient reactivity whilst maintaining stereoselectivity, and control of regioselectivity (C(4)- *vs.* C(2)- addition). In this manuscript, optimisation of this approach is investigated, with the scope and limitations of this methodology demonstrated for the synthesis of a range of 1,4-DHPs.

## 2. Results and discussion

### 2.1. Reaction optimisation

Preliminary investigations focused on the reaction of *para*-nitrophenyl ester **1** and *N*-benzyl pyridinium salt **2** using tetramisole·HCl **3** (20 mol%) and i-Pr_2_NEt in dichloromethane at room temperature for 20 hours (Table 1, entry 1). Chromatographic purification of the product ester was unsuccessful, presumably as a consequence of hydrolytic degradation. The ester product was therefore derivatised to the corresponding isolable benzyl amide following addition of benzylamine at the end of the catalytic reaction. The desired amide **6** was furnished in good yield (73%) with moderate diastereoselectivity (70 : 30 dr) but as a racemate. Use of alternative isothiourea catalysts benzotetramisole (BTM) **4** and HyperBTM **5** also gave promising reactivity, but in each case furnished product **6** as a racemic mixture (entries 2 and 3).

**Table 1:** Reaction optimisation

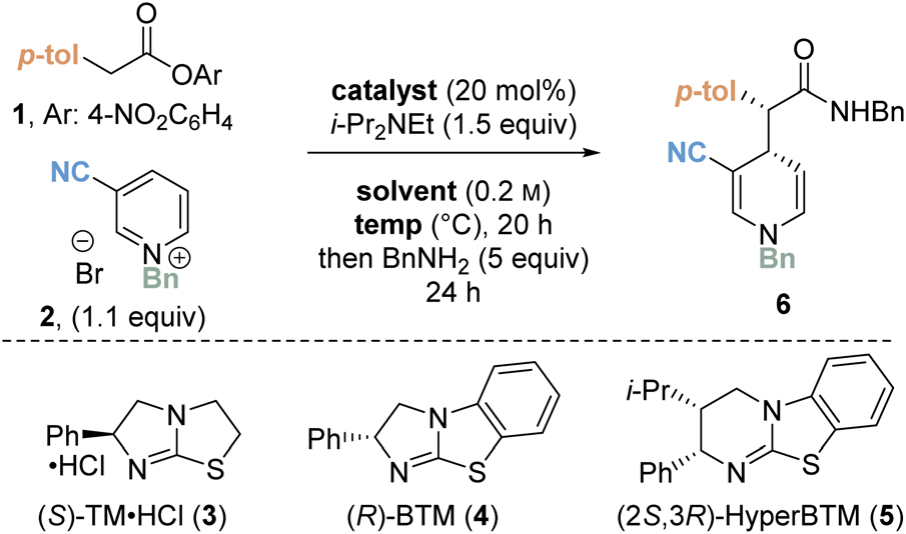						
Entry	Cat.	Solvent	Temp (°C)	Yield^a^ (%)	dr^b^	er^c^
1	**3**	CH_2_Cl_2_	RT	73	70 : 30	50 : 50
2	**4**	CH_2_Cl_2_	RT	70	70 : 30	51 : 49
3	**5**	CH_2_Cl_2_	RT	65	80 : 20	50 : 50
4	**4**	DMF	RT	65	75 : 25	52 : 48
5	**4**	MeCN	RT	70	75 : 25	50 : 50
6	**4**	THF	RT	75	75 : 25	50 : 50
7	**4**	PhMe	RT	74	85 : 15	65 : 35
8	**4**	PhMe	0	48	90 : 10	82 : 18
9	**4**	PhMe	−10	46	85 : 15	82 : 18
10	**4**	PhMe	−40	0	—	—
11	**4**	MeOC_6_H_5_	0	54	95 : 5	68 : 32
12	**4**	ClC_6_H_5_	0	61	90 : 10	73 : 27
13	**4**	FC_6_H_5_	0	59	90 : 10	72 : 28

a Combined yield of major and minor diastereoisomers. Determined by ^1^H NMR analysis of the crude reaction mixture using 1,3,5-trimethoxybenzene as internal standard.

b Determined by ^1^H NMR analysis of the crude reaction mixture.

c Determined by HPLC analysis on a chiral stationary phase.

Variation of the reaction solvent was next investigated. Whilst reactions in DMF, acetonitrile and THF all afforded racemic product (entries 4–6), encouragingly, conducting the reaction in toluene (entry 7) provided the product in high yield (74%) with moderate enantioselectivity (65 : 35 er). Reactions in toluene using isothiourea catalysts TM·HCl **3** and HyperBTM **5** also gave the product as a racemic mixture (see ESI† for further details). Subsequent variation of the reaction temperature (entries 8–10) revealed that 1,4-DHP product **6** could be formed in moderate yield (48%) with improved enantioselectivity (82 : 18 er) at 0 °C. Interestingly, the use of alternative aromatic solvents (anisole, chlorobenzene and fluorobenzene) also gave product **6** with some enantioinduction, albeit in moderate levels (entries 11–13).

Further studies sought to improve the enantioselectivity of the reaction in toluene through variation of the auxiliary base (Table 2). Firstly, the presence of a base was shown to be essential for effective reactivity as only trace amounts of product are observed in its absence (entry 1). This presumably reflects the need to neutralise the HBr generated in the catalytic process and limit deactivation of the isothiourea catalyst by protonation. Compared to Hunig's base (entry 2), use of pentamethylpiperidine (PMP) led to no improvement, giving 1,4-DHP **6** in a similar yield and stereoselectivity (entry 3). Employing triethylamine (entry 4) as the auxiliary base gave amide **6** in lower diastereoselectivity (80 : 20 dr) but with improved enantioselectivity (89 : 11 er). Pleasingly, carrying out the reaction in the presence of DABCO (entry 5) afforded product **6** in improved yield (60%) and stereoselectivity (85 : 15 dr, 91 : 9 er). The use of a weaker organic base, pyridine, led to no conversion of starting materials (entry 6). When the strong organic base polymer supported BEMP was employed, full conversion of starting materials was observed, but no product was detected (entry 7). Furthermore, the use of an inorganic base (caesium carbonate) was also unproductive (entry 8). These results demonstrate that the identity of the auxiliary base has a significant effect on the outcome of the reaction. Further refinement of the reaction conditions through reversing the stoichiometry, increasing the equivalents of *para*-nitrophenyl ester **6**, lowering the reaction concentration (0.15 M) and extending the reaction time to 24 h (entry 9), gave 1,4-DHP **6** in 65% NMR yield with excellent stereoselectivity (90 : 10 dr, 91 : 9 er). Chromatographic purification gave the 1,4-DHP in 46% yield as a single diastereoisomer.

**Table 2:** Optimisation of reaction base

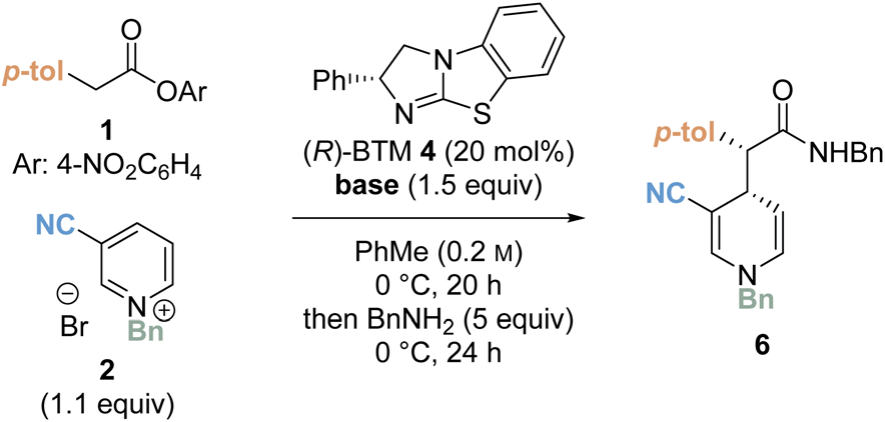				
Entry	Base	Yield^a^ (%)	dr^b^	er^c^
1^d^	None	<10	—	—
2	i-Pr_2_NEt	48	90 : 10	82 : 18
3	PMP	41	90 : 10	83 : 17
4	Et_3_N	45	80 : 20	89 : 11
5	DABCO	60	85 : 15	91 : 9
6	Pyridine	0	—	—
7	PS-BEMP	0	—	—
8	Cs_2_CO_3_	<10	—	—
9^e^	DABCO	65 (46)	90 : 10	91 : 9

a Combined yield of major and minor diastereoisomers. Determined by ^1^H NMR analysis of the crude reaction mixture using 1,3,5-trimethoxybenzene as internal standard. Isolated yield of major diastereoisomer in parentheses.

b Determined by ^1^H NMR analysis of the crude reaction mixture.

c Determined by HPLC analysis on a chiral stationary phase.

d Room temperature.

e 1.5 equiv. 1, 1.0 equiv. 2, 0.15 M concentration, 24 h.

As a final optimisation step, the effect of the counterion of the pyridinium salt was investigated. A series of pyridinium salts were prepared through counterion exchange and then subjected to the catalytic reaction to examine the effect on the yield and stereoselectivity (Table 3). In comparison to the parent bromide pyridinium salt **2** (entry 1), the use of a similarly co-ordinating chloride counterion containing pyridinium salt **7** proved amenable in the catalysis protocol, affording product **6** in comparable yields and stereoselectivity (entry 2). However, use of larger, non-coordinating counterions **8** (PF_6_^−^) and **9** (BF_4_^−^) led to significantly diminished yields, and lower er (entries 3 and 4). A similar reduction in enantioselectivity with change in counterion was observed in our previous work concerning the isothiourea-promoted addition of esters to tetrahydroisoquinoline-derived iminium ions or acyclic iminium ions.^8^

**Table 3:** Probing the effect of the counterion

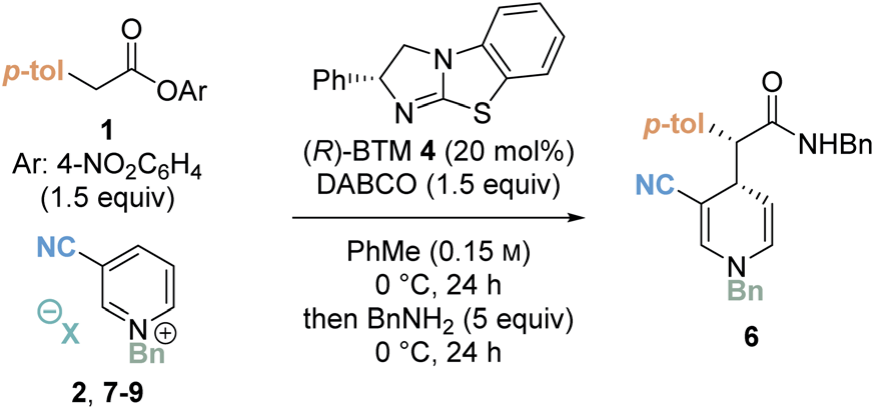				
Entry	X	Yield^a^ (%)	dr^b^	er^c^
1	Br (**2**)	65	90 : 10	91 : 9
2	Cl (**7**)	58	90 : 10	91 : 9
3	PF_6_ (**8**)	22	nd	83 : 17
4	BF_4_ (**9**)	14	nd	84 : 16

a Combined yield of major and minor diastereoisomers. Determined by ^1^H NMR analysis of the crude reaction mixture using 1,3,5-trimethoxybenzene as internal standard.

b Determined by ^1^H NMR analysis of the crude reaction mixture.

c Determined by HPLC analysis on a chiral stationary phase (nd: not determined).

### 2.2. Reaction scope and limitations

Next, the generality of the transformation was investigated by exploring the scope of the pyridinium salt component (Table 4). Firstly, the effect of the electron withdrawing group was examined (Table 4A). Previous organocatalytic dearomatisation protocols for the synthesis of 1,4-DHPs have been shown to be sensitive to changes in electron-withdrawing group, resulting in reduced reactivity and/or stereoselectivity, and have been limited to nitro, nitrile and acetyl substituents.^22–25^ Whilst the use of ethyl ester substituted salt **16** gave 1,4-DHP **25** as a racemate, employing pyridinium salt **17** bearing a 3-phenylsulfonyl substituent gave 1,4-DHP **26** in good yield (58%, isolated) with excellent stereocontrol (90 : 10 dr, 94 : 6 er). An electron withdrawing substituent in the 3-position is necessary for reactivity, as unsubstituted, 3-chloro- and 3-amido substituted pyridinium salts **21–23** were all unreactive in this protocol, with only starting materials returned. This presumably reflects the reduced electrophilicity of these substrates compared to the 3-phenylsulfonyl derivative. Next, the scope of the process was tested by variation of the N-substituent within the alkyl-pyridinium salts. Employing 3-phenylsulfonyl-substituted pyridinium salts, electron-neutral 2-naphthyl substituted salt **18** gave the corresponding amide **27** in high yield (60%, isolated) with excellent stereoselectivity (90 : 10 dr, 94 : 6 er). Benzyl pyridinium salts substituted with an electron donating group (4-methoxy) also proved amenable, furnishing amide **28** in good yield (65%) with high stereoselectivity. 3-Fluoro substituted 1,4-DHP **29** could also be accessed with high stereocontrol (90 : 10 dr, 93 : 7 er). However, the isolated yield in this case (31%) was substantially lower than that observed by ^1^H NMR analysis of the crude reaction mixture (57%), due to difficult product isolation. An *N*-benzyl derived substituent was essential for effective reactivity in this protocol; using *N*-methyl pyridinium salt **24**, no desired product was formed, with only starting materials returned. We speculate this could potentially be due to (i) alkylation of the isothiourea catalyst with **24** and inhibition of the catalytic reaction;^26^ or (ii) stabilising π–cation or π–π interactions in the transition state involving the benzyl group;^27^ however this was not investigated further.

**Table 4:** Reaction scope^a^

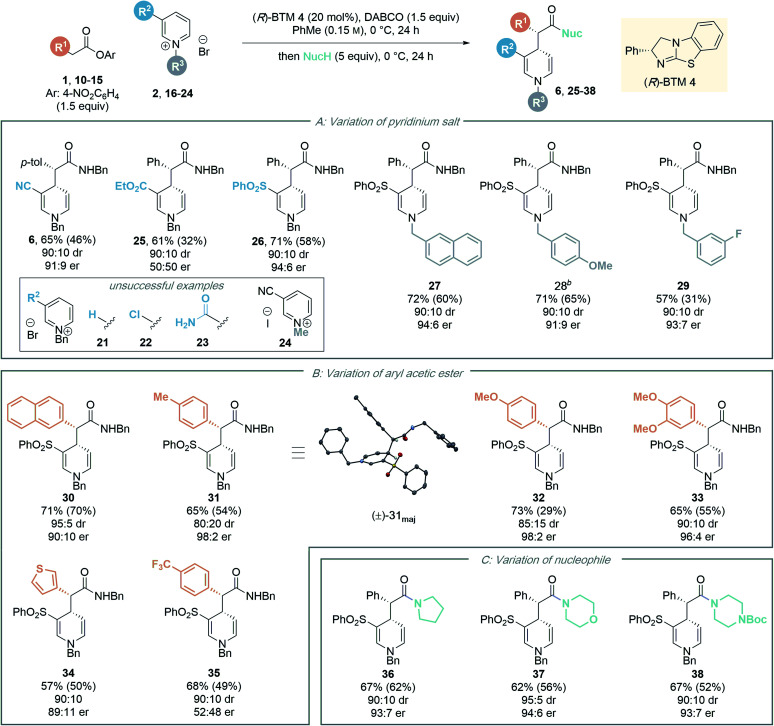

a Combined yield of major and minor diastereoisomers determined by ^1^H NMR analysis of the crude reaction mixture using 1,3,5-trimethoxybenzene as internal standard; isolated yield in parenthesis; dr determined by ^1^H NMR analysis of the crude reaction mixture; er determined by HPLC analysis on a chiral stationary phase.

b Using the corresponding pyridinium benzyl chloride as starting material.

The scope of the developed process was further tested by variation of the ester component (Table 4B). A range of substituted aryl acetic *para*-nitrophenyl esters with different steric and electronic properties was explored. Esters containing a 2-naphthyl or 4-tolyl substituent provided the corresponding amide products **30** and **31** in good yield and diastereoselectivity, and in high enantiomeric ratio (90 : 10 er and 98 : 2 er, respectively). The relative configuration of the major diastereoisomer **31** was determined by single crystal X-ray crystallography of a racemic product,^28^ with the absolute configuration assigned by analogy to the known stereochemical outcome of (*R*)-BTM **4** in reactions of C(1)-ammonium enolates.^1*c*^ The configuration within all other products were assigned by analogy. The incorporation of electron-donating aryl substituents was also tolerated in the methodology, with 4-methoxyphenyl and 3,4-dimethoxyphenyl substituted esters furnishing the corresponding 1,4-DHP's **32** and **33** with high stereocontrol (98 : 2 and 96 : 4 er respectively).^29^ The use of a heterocyclic 3-thiophenyl substituted ester was also successful, giving 1,4-DHP **34** in 50% yield with high stereocontrol (90 : 10 dr, 89 : 11 er). Notably, aryl substituents incorporating an electron-withdrawing 4-trifluoromethylphenyl substituent within the ester proved incompatible with this enantioselective dearomatisation methodology. While significant product was isolated (49% yield) the 4-trifluoromethylphenyl substituted product **35** was racemic. Interestingly, a significant base-promoted background reaction was confirmed in toluene in this case by carrying out a control reaction with no catalyst present (see ESI† for further details). This suggests a narrow reactivity window for the aryl ester component. With a strongly electron withdrawing 4-trifluoromethyl substituent the ester is acidic and a racemic background reaction is facilitated, while electron rich aryl substituents may play a significant part in promoting enantioselectivity by participating in cation–π interactions with the pyridinium ion while minimising competitive background reaction processes.

**Scheme 4:**
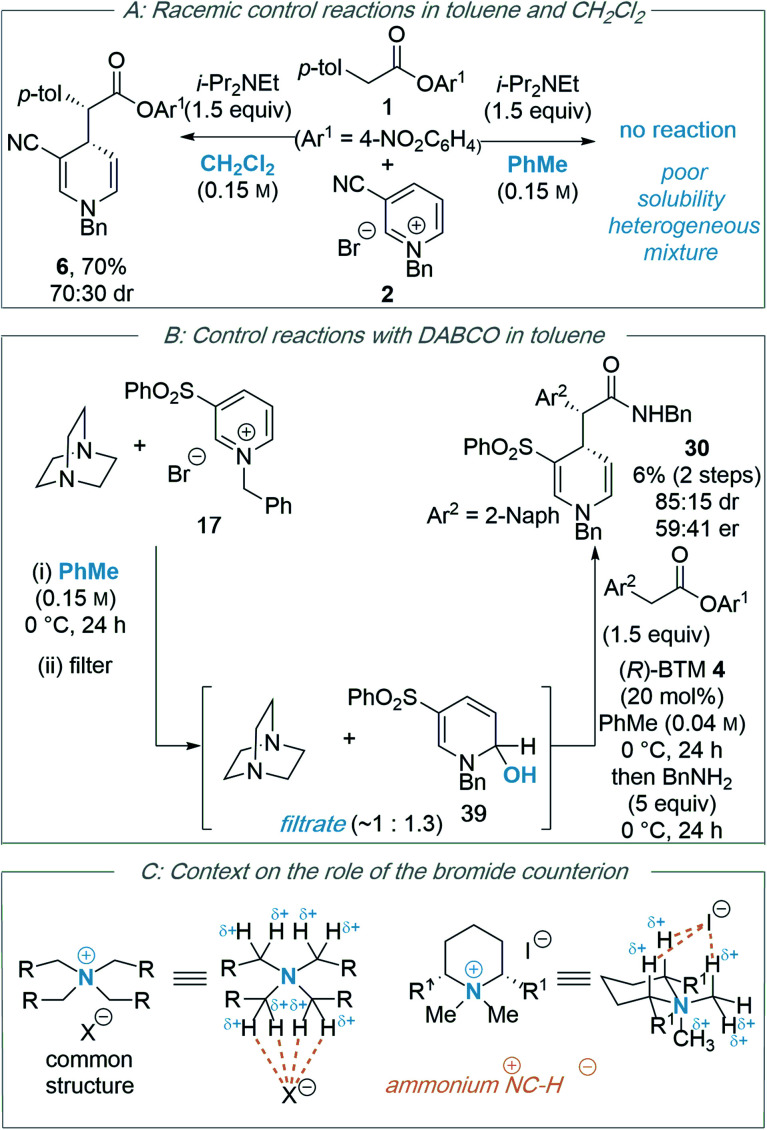
Control studies and further context for this work.

To further demonstrate the synthetic utility of the dearomatisation protocol, a range of nucleophiles were added at the end of the catalytic reactions allowing access to various carbonyl motifs (Table 4C). Amine nucleophiles were demonstrated to be generally applicable: addition of pyrrolidine, morpholine and *N*-Boc piperazine enabled access to amides **36–38** in good isolated yields (up to 62%) with high diastereo- and enantioselectivities (>90 : 10 dr, >93 : 7 er).^30^

### 2.3. Further control studies and mechanistic discussion

During the optimisation of this process, the nature of the base (DABCO proving optimal), solvent (toluene being key for any product enantioselectivity) and the counterion of the *N*-alkyl pyridinium salt (bromide or chloride) proved critical for effective reactivity. The significance of the solvent and the counterion, alongside further control experiments, is discussed below.

#### 2.3.1. Significance of toluene as reaction solvent

One notable feature of this process is the requirement for toluene as a solvent in order to affect significant enantioselectivity. Notably, the use of toluene is consistent with three of the manuscripts illustrated in Scheme 2B that demonstrate enantioselective organocatalytic pyridinium dearomatisation processes for optimal enantioselectivity.^22,23,25^ Firstly, in toluene the pyridinium salts show limited solubility resulting in a heterogeneous reaction mixture, while for example in dichloromethane the reaction is more homogeneous. Control reactions (Scheme 4A) in the absence of isothiourea catalyst (*R*)-BTM **4** indicated a significant base catalysed racemic background reaction occurred in dichloromethane (70% yield, 70 : 30 dr), but not in toluene (less than 5% product observed). The high conversion observed in dichloromethane in this control reaction, coupled with poor enantioselectivity in the isothiourea catalysed process, is consistent with a competitive racemic background reaction in dichloromethane that is generally inhibited in toluene presumably due to limited solubility.

Further control studies in both toluene and CH_2_Cl_2_ probed alternative roles of the isothiourea Lewis base and DABCO. For example, Bernardi and co-workers have previously described cinchona-derived catalysts as nucleophilic promoters through adding to the pyridinium salt,^22^ while reversible NHC addition to pyridinium salts was observed by Rovis.^24^ In this case, control studies between (*R*)-BTM **4** and pyridinium salt **17** in both dichloromethane or toluene showed no addition of the Lewis base catalyst to the pyridinium electrophile (see ESI†). Further control experiments were performed using the stoichiometric addition of DABCO to *N*-benzyl pyridinium bromide **17** (Scheme 4B). In toluene, mixing these reactants gave a heterogeneous mixture; filtration gave a solid that was identified as pyridinium salt **17**, while the filtrate gave a mixture of DABCO and hydroxy-dihydropyridine derivative **39**. Although **39** could not be purified to homogeneity it was characterised from this mixture, with its constitution confirmed by a combination of ^1^H, ^13^C and 2-D NMR techniques, and presumably arises from addition of adventitious water under the reaction conditions (see ESI†). Previous reports have described the *in situ* generation of related hydroxy-dihydropyridine “pseudo-base” derivatives by treatment of pyridinium salts with aqueous base,^31^ and Gündel and co-workers have demonstrated their use as precursors in selective nucleophilic addition of carboxamides to generate the corresponding 1,4-DHP species.^32^ The use of a crude mixture containing hydroxy-dihydropyridine derivative **39** and DABCO as a potential precursor was next tested. Through treatment of this mixture with an excess of 2-Naph-substituted ester **11** (1.5 equivalents) in the presence of (*R*)-BTM **4** (20 mol%) without additional DABCO, followed by addition of benzylamine post catalysis, product **30** was isolated in 6% yield, 85 : 15 dr and 59 : 41 er. While this indicates that hydroxy-dihydropyridine **39** is a potential soluble intermediate, the low yield and enantioselectivity indicate this may be only a minor reaction pathway. Furthermore, the reversible formation of pyridinium ion **17***in situ* from **39** cannot be ruled out.^33^ No similar product could be isolated from the corresponding reaction of DABCO and *N*-benzyl pyridinium bromide **17** in dichloromethane, with addition of DABCO to dichloromethane, and starting materials, observed by ^1^H NMR analysis (see ESI†).

#### 2.3.2. Significance of halide counterion

Bromide and chloride counterions were shown to be beneficial for both reactivity (conversion to product) and enantioselectivity in comparison to alternative non-coordinating counterions (PF_6_^−^, BF_4_^−^). Potential effects of this counterion upon the assumed ammonium enolate generated in this process, and the pyridinium salt, are sequentially considered.

In recent work by Lassaletta and co-workers, similar variation in reactivity and enantioselectivity has been observed in the dearomatization of isoquinolines with *N-tert*-butylhydrazones using thiourea catalysis.^34^ While a chloride counterion led to high enantioselectivity and reactivity (99 : 1 er, >95 : 5 dr, 84% yield), a tetrafluoroborate counterion gave reduced reactivity and selectivity (87 : 13 er, 95 : 5 dr, 22% yield). The origin of this effect was proposed to be due to binding of chloride to the thiourea and reactants, leading to a highly ordered transition structure stabilised by multiple non-covalent C–H/N–H⋯Cl interactions. Interestingly, the well-documented halide binding ability of electron deficient *N*-benzyl pyridinium salts has been used in catalysis,^35^ facilitating the addition of silyl ketene acetals to 1-chloroisochromans and related electrophiles.^36^ Interestingly, Maruoka and co-worker have developed tetra-alkylammonium salts as hydrogen-bonding catalysts using their known ability to behave as distributed cations (through formal ^+^NC–H⋯X^−^ interactions with donor anions)^37^ as pioneered by Reetz (Scheme 4C).^38^ The delocalization of positive charge in such ammonium salts is so extensive that these adjacent C–H bonds have a similar donor ability to alcohols,^39^ allowing them to potentially interact with hydrogen bond acceptors such as halide counterions. Taking these precedents and concepts into consideration, within an assumed isothiouronium enolate intermediate, the formal positive charge can be delocalised between the two nitrogen atoms derived from the isothiourea catalyst, allowing the adjacent C–H bonds to potentially play a key structural part in enantiorecognition through ^+^NC–H⋯Br^−^ complexation, and this is built into our proposed model for enantiocontrol. Notably, in previous work using isothiourea catalysis to promote enantioselective 2,3-rearrangements of allylic ammonium ylides computational analysis showed that inclusion of such ^+^NC–H⋯Br^−^ bromide co-ordination within the transition state was feasible, with further stabilising ArC–H⋯Br^−^ interactions from the *ortho*-position of the stereodirecting phenyl substituent also observed.^7*d*^

#### 2.3.3 Proposed mechanism

**Scheme 5:**
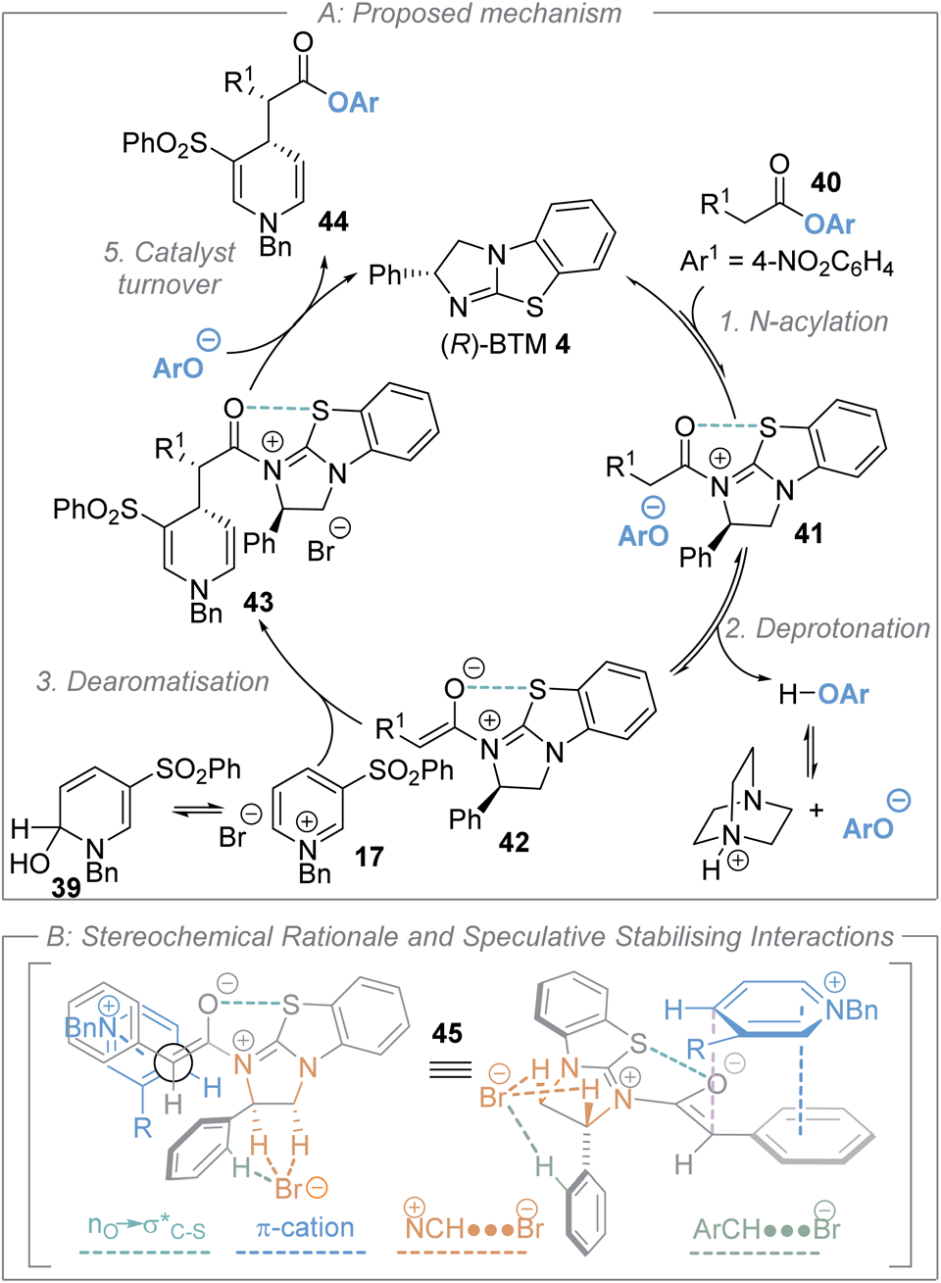
Proposed mechanism.

Taking all of these factors into consideration, a simplified reaction mechanism for the developed procedure can be proposed. Analogous to similar isothiourea-catalysed processes *via* C(1)-ammonium enolate intermediates using aryl ester precursors, catalysis is initiated by acylation of the isothiourea catalyst (*R*)-BTM **4** by the aryl ester **40** to afford acyl ammonium ion pair **41** (Scheme 5). Deprotonation of the acyl ammonium intermediate by the aryloxide then occurs to afford C(1)-ammonium enolate **42**. The phenol released from step two can be deprotonated by the DABCO auxiliary base regenerating the aryloxide. Regioselective addition of the C(1)-ammonium enolate to the 4-position of either the benzyl pyridinium salt **17** or hydroxy-dihydropyridine derivative **39** generates acyl ammonium **43**. For simplicity, despite its poor solubility in toluene the use of benzyl pyridinium bromide salt **17** in low concentration is invoked rather than hydroxy-dihydropyridine **39**. However, the intermediacy of **39** cannot be conclusively excluded. Catalyst turnover can be achieved through addition of the aryloxide to intermediate **43** to release BTM **4** and generate the product **44**. The observed diastereo- and enantioselectivity can be rationalised tentatively by pre-transition state assembly **45**, where steric interactions are minimized about the forming C–C bond.^40^ Key additional factors that contribute to selectivity include 1,5-S⋯O chalcogen bonding,^41–43^ π–cation, as well as ^+^NC–H⋯Br^−^ interactions. Primarily, a 1,5-S⋯O interaction (
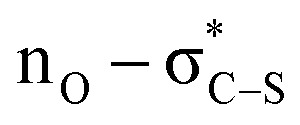
 delocalisation) between the enolate O and catalyst S substituents provides a conformational lock.^44^ Pyridinium salts are recognised anion receptors,^45^ with anion–π interactions,^46^ C–H directed anion–π interactions^47^ and pyridinium C–H–anion interactions,^48^ all observed and proposed. However, in this model, the widely recognised ability of pyridinium salts to interact with donor π-systems (in this instance derived from the aryl acetic ester substituent) through a π–cation-interaction is considered dominant. This is also consistent with highest enantioselectivity being observed with the introduction of electron rich aryl substituents within the ester component of the reaction. The observation of highest enantioselectivity with a bromide (or chloride) counterion is tentatively attributed to stabilising ^+^NC–H⋯X^−^ and ArC–H⋯X^−^ interactions that may potentially help to solubilise the pyridinium salt and promote reactivity. Further long-range interactions that involve pyridinium ion substituents with the bromide counterion may also play a role but remain speculative.

## 3. Conclusions

In conclusion, a procedure for the regio-, diastereo- and enantioselective dearomatisation of pyridinium salts using isothiourea catalysis *via* C(1)-ammonium enolates has been developed. This broadens the scope of compatible electrophiles in isothiourea catalysis *via* C(1)-ammonium enolates, which had been previously limited to alkene and carbonyl derivatives, enabling the synthesis of enantioenriched 1,4-DHP heterocyclic motifs. This protocol required extensive optimisation to achieve high enantioselectivities alongside synthetically useful product yields. Key factors in the optimisation included the essential use of toluene as the reaction solvent, DABCO as the auxiliary base, bromide as a counterion and carrying out the reaction at low temperature (0 °C). The developed methodology was employed for the synthesis of a suite of 1,4-DHPs in good yield with excellent stereocontrol (15 examples, up to 70% isolated yield, 95 : 5 dr, 98 : 2 er).

## Data availability

The research data underpinning this publication can be found at https://doi.org/10.17630/e7825bd7-6a4a-41c4-a017-1fe615471562.

## Author contributions

Conceptualization, A. D. S. and C. M.; investigation, C. M.; J. B.; L. J. B.; X-ray crystallographic analysis, A. M. Z. S.; writing—original draft preparation, C. M.; writing—review and editing, A. D. S. and all authors; supervision and funding acquisition, A. D. S.

## Conflicts of interest

There are no conflicts to declare.

## Supplementary Material

SC-012-D1SC03860E-s001

SC-012-D1SC03860E-s002
