# Supplementary material for: Catalytic enantioselective synthesis of 1,4-dihydropyridines via the addition of C(1)-ammonium enolates to pyridinium salts
Source: Chem Sci. 2021 Aug 6;12(36):12001–11. doi: 10.1039/d1sc03860e (PMC8457386; doi:10.1039/d1sc03860e)
Supplement: SC-012-D1SC03860E-s001 [file SC-012-D1SC03860E-s001.pdf]

Electronic Supplementary Material (ESI) for Chemical Science.
This journal is © The Royal Society of Chemistry 2021

Catalytic Enantioselective Synthesis of 1,4-Dihydropyridines via the Addition of C(1)-Ammonium Enolates to Pyridinium Salts

Calum McLaughlin, Jacqueline Bitai, Lydia J. Barber, Alexandra M. Z. Slawin and Andrew D. Smith*

Supporting Information

1. General experimental 2

2. General procedures 5

3. Synthesis of aryl esters 7

4. Synthesis of pyridinium salts 11

5. Synthesis of catalysis products 19

6. Control experiments 36

7. Additional optimisation studies 41

8. X-Ray structure 42

9. References 43

10. Appendix I : ^{1}H and $^{13}C\{^{1}H\}$ NMR spectra of novel compounds 44

11. Appendix II. HPLC traces 69

1. General experimental

All reagents and solvents were obtained from commercial suppliers and were used as received without further purification unless otherwise stated. Purification was carried out according to standard laboratory methods. Tetramisole (TM) HCl **3** was purchased from Sigma-Aldrich, benzotetramisole (BTM) **4** and HyperBTM **5** were prepared in house.[1,2] All diastereomeric ratios (dr) of crude reaction mixtures analysed by ^{1}H NMR are reported to the nearest multiple of 5.

1.1. Purification of solvents

Anhydrous solvents (CH_2Cl_2, THF, PhMe) were obtained after passing through an alumina column (Mbraun SPS-800). Anhydrous MeCN was purchased from Sigma-Aldrich and used without further purification. Anhydrous DMF was purchased from Acros and used without further purification. Petrol is defined as petroleum ether 40-60 °C. EtOAc, Et_2O, CH_2Cl_2 and petrol for purification purposes were used as obtained from suppliers without further purification.

1.2. Purification of reagents

Dry Hunig's base was obtained by distillation over KOH and transferred to and stored in a screw-top vial over KOH and purged with and stored under nitrogen.

1.3. Experimental details

Reactions were carried out in flame-dried glassware under an inert atmosphere (N_2) using standard vacuum line techniques. Purging refers to a vacuum/nitrogen-refilling procedure. Room temperature (RT) refers to 20-25 °C. Temperatures of 0 °C and were obtained using an ice/water. Temperatures of 0 °C, −10 °C and −40 °C for catalysis reactions were obtained using an immersion cooler (HAAKE EK 90). Reactions involving heating were performed using DrySyn blocks, sand, and a contact thermocouple. Under reduced pressure refers to the use of either a Büchi Rotavapor R-200 with a Büchi V-491 heating bath and Büchi V-800 vacuum controller, a Büchi Rotavapor R-210 with a Büchi V-491 heating bath and Büchi V-850 vacuum controller, a Heidolph Laborota 4001 with vacuum controller, an IKA RV10 rotary evaporator with a IKA HB10 heating bath and

ILMVAC vacuum controller, or an IKA RV10 rotary evaporator with a IKA HB10 heating bath and Vacuubrand CVC3000 vacuum controller. Rotary evaporator condensers are fitted to Julabo FL601 Recirculating Coolers filled with ethylene glycol and set to –5 °C. In vacuo refers to the use of a Schlenk line manifold and high vacuum pump.

1.4. Purification of products

Analytical thin layer chromatography was performed on pre-coated aluminium plates (Kieselgel 60 F254 silica) and visualisation was achieved using ultraviolet light (254 nm) and/or staining with either aqueous $KMnO_4$ solution or ethanolic Vanillin solution followed by heating. Manual column chromatography was performed in glass columns fitted with porosity 3 sintered discs over Kieselgel 60 silica using the solvent system stated. Automated chromatography was performed on a Biotage Isolera Four running Biotage OS578 with a UV/Vis detector using the method stated and cartridges filled with Kieselgel 60 silica. Purification of catalysis products using a gradient between 0-10% Et_2O in CH_2Cl_2 was carried out using a stock solution of 10% Et_2O in CH_2Cl_2, diluted appropriately with CH_2Cl_2.

1.5. Analysis of products

Melting points (mp) were recorded on an Electrothermal 9100 melting point apparatus, (dec) refers to decomposition.

Optical rotations $[\alpha]_D^{20}$ were measured on a Perkin Elmer Precisely/Model-341 polarimeter operating at the sodium D line with a 100 mm path cell at 20 °C.

HPLC analyses were obtained on either a Shimadzu HPLC consisting of a DGU-20A$_5$ degassing unit, LC-20AT liquid chromatography pump, SIL-20AHT autosampler, CMB-20A communications bus module, SPD-M20A diode array detector and a CTO-20A column oven or a Shimadzu HPLC consisting of a DGU-20A$_{5R}$ degassing unit, LC-20AD liquid chromatography pump, SIL-20AHT autosampler, SPD-20A UV/Vis detector and a CTO-20A column oven. Separation was achieved using either DAICEL CHIRALCEL OD-H and OJ-H columns or DAICEL CHIRALPAK AD-H, AS-H, IA, IB, IC and ID columns using the method stated. HPLC traces of enantiomerically enriched compounds were compared with authentic racemic spectra.

Infrared spectra (ν_{max}) were recorded on a Shimadzu IRAffinity-1 Fourier transform IR spectrophotometer fitted with a Specac Quest ATR accessory (diamond puck). Spectra were recorded of either thin films or solids, with characteristic absorption wavenumbers (ν_{max}) reported in cm^{-1}.

^{1}H, ^{13}C{^{1}H}, and ^{19}F NMR spectra were acquired on either a Bruker AV400 with a BBFO probe (^{1}H 400 MHz; ^{13}C{^{1}H} 101 MHz; ^{19}F{^{1}H} 377 MHz), a Bruker AVII 400 with a BBFO probe (^{1}H 400 MHz; ^{13}C{^{1}H} 101 MHz; ^{19}F{^{1}H} 376 MHz), a Bruker AVIII-HD 500 with a SmartProbe BBFO+ probe (^{1}H 500 MHz, ^{13}C{^{1}H} 126 MHz, ^{19}F{^{1}H} 470 MHz), or a Bruker AVIII 500 with a CryoProbe Prodigy BBO probe (^{1}H 500 MHz, ^{13}C{^{1}H} 126 MHz, ^{19}F 470 MHz), in the deuterated solvent stated. All chemical shifts are quoted in parts per million (ppm) relative to the residual solvent peak. All coupling constants, *J*, are quoted in Hz. Multiplicities are indicated as s (singlet), d (doublet), t (triplet), q (quartet), m (multiplet), and multiples thereof. The abbreviation Ar denotes aromatic and app denotes apparent. NMR peak assignments were confirmed using 2D ^{1}H correlated spectroscopy (COSY), 2D ^{1}H-^{13}C heteronuclear single quantum coherence (HSQC), 2D ^{1}H-^{13}C heteronuclear multiple-bond correlation spectroscopy (HMBC), 2D ^{1}H total correlation spectroscopy (TOCSY) and 2D ^{1}H nuclear Overhauser effect spectroscopy (NOESY), where necessary.

High resolution mass spectrometry (HRMS) data were acquired by either electrospray ionisation (ESI), electron impact (EI), atmospheric solids analysis probe (ASAP), or nanospray ionisation (NSI) at either the University of St Andrews Mass Spectrometry Facility, the EPSRC UK National Mass Spectrometry Facility at Swansea University, or at the School of Chemistry University of Edinburgh mass spectrometry service.

2. General procedures

2.1. General procedure A: synthesis of esters

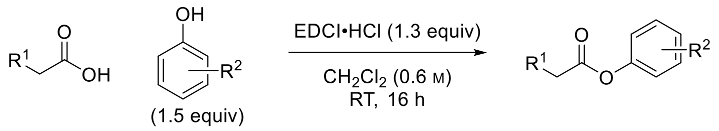

The appropriate acetic acid (1.0 equiv) and EDCI·HCl (1.3 equiv) were dissolved in anhydrous CH_2Cl_2 (0.6 M) and the reaction mixture was stirred at room temperature. After 10 mins, the appropriate phenol (1.5 equiv) was added, and the reaction stirred at room temperature for 16 h. The reaction mixture was diluted with H_2O and extracted with CH_2Cl_2 (3 ×). The organic layer was dried with $MgSO_4$, filtered, and concentrated under reduced pressure to give the crude product that was purified by flash silica column chromatography.

2.2. General procedure B: synthesis of pyridinium salts

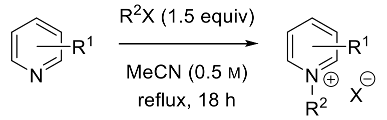

The appropriate electrophile (1.5 equiv) was added to the appropriate pyridine (1.0 equiv) in MeCN (0.5 M) and the reaction was refluxed for 18 h. The reaction mixture was cooled to 0 °C and cold Et_2O was added with stirring until a precipitate formed. The solid was filtered, washed with cold Et_2O and dried in vacuo.

2.3. General procedure C: optimisation of the dearomatisation reaction

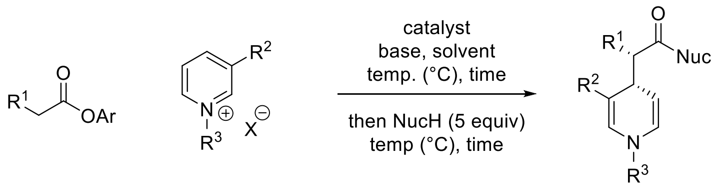

The appropriate ester, pyridinium salt, base (if solid) and catalyst were dissolved in solvent. The appropriate base (if liquid) was added, and the reaction was stirred at the stated temperature for the required time. The appropriate nucleophile (5.0 equiv) was added and the reaction was stirred at the stated temperature for the required time. The

reaction mixture was quenched with 1 M NaOH (10 mL) and extracted with CH_2Cl_2 (3 × 10 mL). The organic layer was washed successively with 1 M NaOH (2 × 10 mL) and brine (10 mL), dried over $MgSO_4$, filtered, and concentrated under reduced pressure. The crude product was purified by flash silica column chromatography.

2.4. General procedure D: isothiourea-catalysed dearomatisation of pyridinium salts

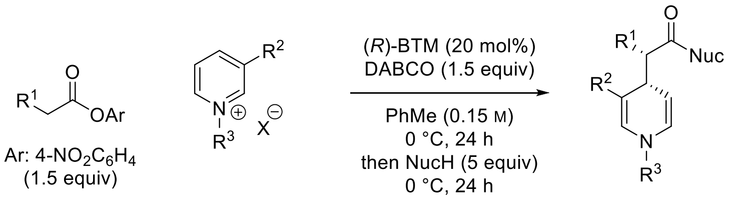

The appropriate ester (1.5 equiv), electrophile (1.0 equiv), DABCO (1.5 equiv) and catalyst (20 mol%) were weighed into a 20 mL test tube. The test tube was sealed, purged, lowered into a cryostat bath at 0 °C. PhMe (0.15 M) was added and the reaction was stirred at 0 °C for 24 h. The appropriate nucleophile (5.0 equiv) was added and the reaction was stirred at 0 °C for a further 24 h. The reaction mixture was quenched with 1 M NaOH (20 mL) and extracted with CH_2Cl_2 (3 × 20 mL). The organic layer was washed successively with 1 M NaOH (2 × 10 mL) and brine (10 mL), dried over $MgSO_4$, filtered, and concentrated under reduced pressure. The crude product was purified by column chromatography.

3. Synthesis of aryl esters

4''-Nitrophenyl 2-(4'-tolyl)acetate (**1**)

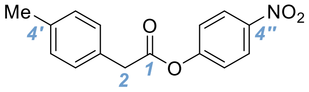

1

Following general procedure A, using 2-(*p*-tolyl)acetic acid (5.00 g, 34 mmol, 1.0 equiv), EDCI·HCl (8.40 g, 44 mmol, 1.3 equiv), 4-nitrophenol (7.00 g, 50 mmol, 1.5 equiv) and CH_2Cl_2 (56 mL, 0.6 M) for 22 h gave, after purification by column chromatography (CH_2Cl_2, R_f 0.58), the title compound (5.81 g, 64%) as a colourless solid with spectroscopic data in accordance with the literature.[3] **mp** 60-62 °C {Lit.[4] 60-62 °C}; **^{1}H NMR** (400 MHz, $CDCl_3$) δ_H: 2.39 (3H, s, C*H*$_3$), 3.88 (2H, s, C(2)*H*$_2$), 7.22 (2H, d, *J* 8.0, ArC(3',5')*H*), 7.24 – 7.32 (4H, m, ArC(2',6')*H* and ArC(2'',6'')*H*), 8.27 (2H, d, *J* 9.2, ArC(3'',5'')*H*).

4''-Nitrophenyl 2-phenylacetate (**10**)

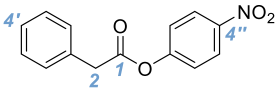

10

Following general procedure A, using phenylacetic acid (5.44 g, 40 mmol, 1.0 equiv), EDCI·HCl (9.97 g, 52 mmol, 1.3 equiv), 4-nitrophenol (8.35 g, 60 mmol, 1.5 equiv) and CH_2Cl_2 (67 mL, 0.6 M) for 21 h gave, after purification by column chromatography (CH_2Cl_2, R_f 0.6), the title compound (7.28 g, 71%) as a colourless solid with spectroscopic data in accordance with the literature.[5] **mp** 58-60 °C {Lit.[4] 58-60 °C}; **^{1}H NMR** (400 MHz, $CDCl_3$) δ_H: 3.90 (2H, s, C(2)*H*$_2$), 7.26 (2H, d, *J* 9.2, ArC(2',6')*H*), 7.28 – 7.46 (5H, m, ArC*H*), 8.25 (2H, d, *J* 9.2, C(3'',5'')*H*).

4''-Nitrophenyl 2-(naphthalen-2'-yl)acetate (**11**)

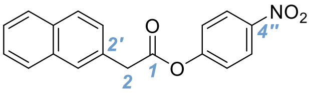

11

Following general procedure A, using 2-naphthylacetic acid (1.86 g, 10 mmol, 1.0 equiv), EDCI·HCl (2.50 g, 13 mmol, 1.3 equiv), 4-nitrophenol (2.09 g, 15 mmol, 1.5 equiv) and CH_2Cl_2 (17 mL, 0.6 M) for 72 h gave, after purification by Biotage® Isolera™ 4 [SNAP KP-

Sil 50 g, 100 mL min^{-1}, CH_2Cl_2 in Petrol (50% 5 CV, 50-75% 10 CV), R_f 0.68 at 100% CH_2Cl_2] the title compound (2.21 g, 72%) as a colourless solid with spectroscopic data in accordance with the literature.[6] **mp** 96-98 °C {Lit.[7] 95-97 °C}; **^{1}H NMR** (400 MHz, $CDCl_3$) δ_H: 4.09 (2H, s, C(2)H_2), 7.28 (2H, d, *J* 9.3, C(2'',6'')*H*), 7.45 – 7.62 (3H, m, ArC*H*), 7.79 – 7.97 (4H, m, ArC*H*), 8.27 (2H, d, *J* 9.2, C(3'',5'')*H*).

4''-Nitrophenyl 2-(4'-methoxyphenyl)acetate (**12**)

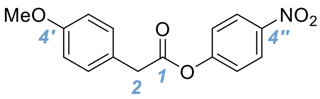

12

Following general procedure A, using 4-methoxyphenylacetic acid (0.66 g, 4 mmol, 1.0 equiv), EDCI·HCl (1.0 g, 5.2 mmol, 1.3 equiv), 4-nitrophenol (0.84 g, 6 mmol, 1.5 equiv) and CH_2Cl_2 (6.7 mL, 0.6 M) for 21 h gave, after purification by column chromatography (CH_2Cl_2, R_f 0.56), the title compound (0.61 g, 53%) as a colourless solid with spectroscopic data in accordance with the literature.[4] **mp** 89-91 °C {Lit.[4] 88-90 °C}; **^{1}H NMR** (400 MHz, $CDCl_3$) δ_H: 3.82 (3H, s, CH_3), 3.84 (2H, s, C(2)H_2), 6.92 (2H, d, *J* 8.7, C(3',5')*H*), 7.25 (2H, d, *J* 9.2, C(2'',6'')*H*), 7.29 (2H, d, *J* 8.8, C(2',6')*H*), 8.24 (2H, d, *J* 9.2, C(3'',5'')*H*).

4''-Nitrophenyl 2-(3',4'-dimethoxyphenyl)acetate (**13**)

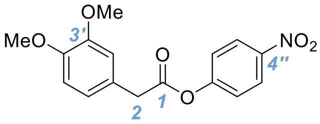

13

Following general procedure A, using 3,4-methoxyphenylacetic acid (3.9 g, 20 mmol, 1.0 equiv), EDCI·HCl (5.00 g, 26 mmol, 1.3 equiv), 4-nitrophenol (4.2 g, 30 mmol, 1.5 equiv) and CH_2Cl_2 (34 mL, 0.6 M) for 20 h gave, after purification by Biotage® Isolera™ 4 [SNAP KP-Sil 50 g, 100 mL min^{-1}, CH_2Cl_2 in petrol (70% 5 CV, 70-80% 7 CV), R_f 0.40 at 100% CH_2Cl_2] the title compound (3.1 g, 49%) as a colourless solid with spectroscopic data in accordance with the literature.[8] **mp** 77-79 °C {Lit.[8] 126-128 °C}; **^{1}H NMR** (500 MHz, $CDCl_3$) δ_H: 3.86 (2H, s, C(2)H_2), 3.91 (3H, s, C(4')OCH_3), 3.92 (3H, s, C(3')OCH_3), 6.89 (1H, d, *J* 8.2, C(5')*H*), 6.91 (1H, d, *J* 1.9, C(2')*H*), 6.94 (1H, dd, *J* 8.1, 2.0, C(6')*H*), 7.28 (2H, d, *J* 9.2,

C(2'',6'')*H*), 8.27 (2H, d, *J* 9.2, C(3'',5'')*H*); **^{13}C{^{1}H} NMR** (126 MHz, $CDCl_3$) δ_C: 40.9 (*C*(2)H_2), 56.0 (C(3')OCH_3), 56.0 (C(4')OCH_3), 111.4 (*C*(5')H), 112.4 (*C*(2')H), 121.7 (*C*(6')H), 122.5 (*C*(2'',6'')H), 125.1 (*C*(1')), 125.3 (*C*(3'',5'')H), 145.4 (*C*(4'')), 148.6 (*C*(4')OCH_3), 149.2 (*C*(3')OCH_3), 155.5 (*C*(1'')), 169.5 (*C*(1)=O). The observed melting point was significantly lower than the one reported. However, we are satisfied that the structure of the compound is correct and of high purity based on the other characterisation data.

4''-Nitrophenyl 2-(thiophen-3'-yl)acetate (**14**)

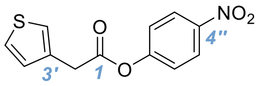

14

Following general procedure A, using 3-thiophene acetic acid (1.42 g, 10 mmol, 1.0 equiv), EDCI·HCl (2.50 g, 13 mmol, 1.3 equiv), 4-nitrophenol (2.10 g, 15 mmol, 1.5 equiv) and CH_2Cl_2 (17 mL, 0.6 M) for 72 h gave, after purification by Biotage® Isolera™ 4 [SNAP KP-Sil 50 g, 100 mL min^{-1}, CH_2Cl_2 in petrol (40-100% 21 CV), R_f 0.62 at 100% CH_2Cl_2] the title compound (1.73 g, 66%) as a colourless solid with spectroscopic data in accordance with the literature.[4] **mp** 55-57 °C {Lit.[4] 55-57 °C}; **^{1}H NMR** (500 MHz, $CDCl_3$) δ_H: 3.95 (2H, s, C(2)*H*$_2$), 7.13 (1H, dd, *J* 5.0, 1.2, C(4')*H*), 7.24 – 7.30 (3H, m, C(2'',6'')*H* and C(2')*H*), 7.36 (1H, dd, *J* 4.9, 3.0, C(5')*H*), 8.26 (2H, d, *J* 9.1, C(3'',5'')*H*).

4''-Nitrophenyl 2-(4'-(trifluoromethyl)phenyl)acetate (**15**)

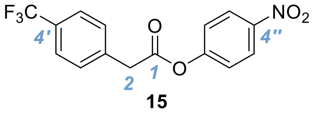

15

Following general procedure A, using 4-(trifluoromethyl)phenylacetic acid (1.63 g, 8 mmol, 1.0 equiv), EDCI·HCl (2.00 g, 10.4 mmol, 1.3 equiv), 4-nitrophenol (1.67 g, 12 mmol, 1.5 equiv) and CH_2Cl_2 (13 mL, 0.6 M) for 21 h gave, after purification by by Biotage® Isolera™ 4 [SNAP KP-Sil 50 g, 100 mL min^{-1}, CH_2Cl_2 in petrol (40% 5 CV, 50-60% 6 CV), R_f 0.72 at 100% CH_2Cl_2] the title compound (1.41 g, 54%) as a colourless solid with spectroscopic data in accordance with the literature.[4] **mp** 71-73 °C {Lit.[4] 65-67 °C}; **^{1}H NMR** (500 MHz, $CDCl_3$) δ_H: 3.98 (2H, s, C(2)*H*$_2$), 7.27 (2H, d, *J* 9.2, C(2'',6'')*H*), 7.51 (2H,

d, *J* 8.1, C(2',6')*H*), 7.65 (2H, d, *J* 8.1, C(3',5')*H*), 8.26 (2H, d, *J* 9.1, C(3'',5'')*H*); **^{19}F NMR** (471 MHz, $CDCl_3$) δ_F: −62.6 (s).

4. Synthesis of pyridinium salts

1-Benzyl-3-cyanopyridin-1-ium bromide (**2**)

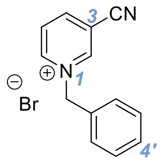

2

Following general procedure B, using benzyl bromide (5.0 mL, 42 mmol, 1.5 equiv), 3-pyridine carbonitrile (2.9 g, 28 mmol, 1.0 equiv) and MeCN (56 mL, 0.5 M) for 18 h gave the title compound (6.10 g, 79%) as a beige solid with spectroscopic data in accordance with the literature.[9] **mp** 144-148 °C {Lit.[9] 151-153 °C}; **^{1}H NMR** (500 MHz, D_2O) δ_H: 5.91 (2H, s, C*H*$_2$), 7.38 – 7.60 (5H, m, C(2',6')*H*, C(3',5')*H* and C(4')*H*), 8.26 (1H, app t, *J* 14.4, C(5)*H*), 8.93 (1H, d, *J* 8.2, C(4)*H*), 9.21 (1H, d, *J* 6.3, C(6)*H*), 9.46 (1H, s, C(2)*H*).

1-Benzyl-3-cyanopyridin-1-ium chloride (**7**)

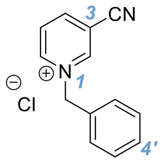

7

1-Benzylpyridin-1-ium bromide **2** (138 mg, 0.5 mmol, 1.0 equiv) and potassium chloride (37 mg, 0.5 mmol, 1.0 equiv) were combined in MeCN (5 mL. 0.1 M) and the reaction was stirred at room temperature for 72 h. The reaction mixture was filtered, washing with MeCN, and concentrated in vacuo to give the title compound (115 mg, quant.) as a beige solid. **mp** 130-132 °C; ν_{max} (solid): 2992 (C-H), 2918 (C-H), 1632, 1493, 1470, 1443, 1206, 1142, 721, 675; **^{1}H NMR** (500 MHz, DMSO) δ_H: 5.94 (2H, s, C*H*$_2$), 7.19 – 7.50 (3H, m, C(3',5')*H* and C(4')*H*), 7.62 (2H, dd, *J* 7.3, 2.1, C(2',6')*H*), 8.38 (1H, dd, *J* 7.9, 6.4, C(5)*H*), 9.13 (1H, app dt, *J* 8.0, 1.2, C(4)*H*), 9.48 (1H, d, *J* 6.3, C(6)*H*), 10.06 (1H, s, C(2)*H*); **^{13}C{^{1}H} NMR** (126 MHz, DMSO) δ_C: 63.9 (CH_2), 113.3 (*C*(3)), 113.9 (C(3)*C*N), 128.9 (*C*(5)H), 129.2 (*C*(2',6')H), 129.3 (*C*(3',5')H), 129.6 (*C*(4')H), 133.4 (*C*(1)), 148.1 (*C*(6)H), 149.0 (*C*(4)H or *C*(6)H), 149.1 (*C*(4)H or *C*(6)H); **HRMS** (ESI$^+$) $C_{13}H_{11}N_2$ [M−Cl]$^+$ found 195.0914, requires 195.0917 (−1.5 ppm).

1-Benzyl-3-cyanopyridin-1-ium hexafluorophosphate(V) (**8**)

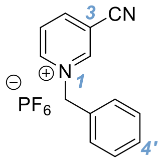

8

1-Benzylpyridin-1-ium bromide **2** (138 mg, 0.5 mmol, 1.0 equiv) and potassium hexafluorophosphate (92 mg, 0.5 mmol, 1.0 equiv) were combined in MeCN (5 mL. 0.1 M) and the reaction was stirred at room temperature for 72 h. The reaction mixture was filtered, washing with MeCN, and concentrated in vacuo to give the title compound (170 mg, quant.) as a peach solid. **mp** 118-120 °C; ν_{max} (solid): 1502, 1495, 1458, 1447, 1200, 1140, 825 (P-F); **^{1}H NMR** (500 MHz, DMSO) δ_H: 5.87 (2H, s, C*H*$_2$), 7.35 – 7.52 (3H, m, C(3',5')*H* and C(4')*H*), 7.58 (2H, dd, *J* 7.4, 2.0, C(2',6')*H*), 8.36 (1H, dd, *J* 8.0, 6.4, C(5)*H*), 9.11 (1H, app dt, *J* 8.1, 1.3, C(4)*H*), 9.39 (1H, d, *J* 6.3, C(6)*H*), 9.97 (1H, s, C(2)*H*); **^{19}F{^{1}H} NMR** (470 MHz, DMSO) δ_F: -70.1 (d, *J* 711.3); **^{13}C{^{1}H} NMR** (126 MHz, DMSO) δ_C: 64.2 (*C*H$_2$), 113.4 (*C*(3)), 113.9 (C(3)CN), 128.9 (*C*(5)H), 129.2 (*C*(2',6')H), 129.2 (*C*(3',5')H), 129.7 (*C*(4')H), 133.4 (*C*(1')), 148.1 (*C*(6)H), 149.0 (*C*(2)H), 149.2 (*C*(4)H); **^{31}P{^{1}H} NMR** (202 MHz, DMSO) δ_P: -144.2 (sept, *J* 711.3); **HRMS** (ESI$^+$) $C_{13}H_{11}N_2$ [M−PF$_6$]$^+$ found 195.0912, requires 195.0917 (−2.6 ppm).

1-Benzyl-3-cyanopyridin-1-ium tetrafluoroborate (**9**)

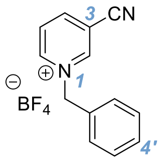

9

1-Benzylpyridin-1-ium bromide **2** (138 mg, 0.5 mmol, 1.0 equiv) and sodium tetrafluoroborate (55 mg, 0.5 mmol, 1.0 equiv) were combined in MeCN (5 mL. 0.1 M) and the reaction was stirred at room temperature for 72 h. The reaction mixture was filtered, washing with MeCN, and concentrated in vacuo to give the title compound (127 mg, 90%) as a white gum. ν_{max} (film): 3088 (ArC-H), 1634, 1497 (ArC=C), 1456, 1206, 1024, 723, 675; **^{1}H NMR** (500 MHz, DMSO) δ_H: 5.88 (2H, s, C*H*$_2$), 7.41 – 7.51 (3H, m, C(3',5')*H* and C(4')*H*),

7.58 (2H, dd, *J* 7.4, 2.0, C(2',6')*H*), 8.36 (1H, dd, *J* 8.0, 6.4, C(5)*H*), 9.11 (1H, d, *J* 8.1, C(4)*H*), 9.40 (1H, d, *J* 6.3, C(6)*H*), 9.98 (1H, s, C(2)*H*); **^{19}F{^{1}H} NMR** (471 MHz, CD_3CN) –151.9, –151.8; **^{13}C{^{1}H} NMR** (126 MHz, DMSO) δ_C: 64.1 (*C*H_2), 113.4 (*C*(3)), 113.9 (CN), 128.9 (*C*(5)H), 129.2 (*C*(2',6')H and *C*(3',5')H), 129.7 (*C*(4')H), 133.4 (*C*(1')), 148.1 (*C*(4)H), 149.0 (*C*(2)H or *C*(6)H), 149.2 (*C*(2)H or *C*(6)H); **HRMS** (ESI$^+$) $C_{13}H_{11}N_2$ $[M–BF_4]^+$ found 195.0914, requires 195.0917 (−1.5 ppm).

1-Benzyl-3-(ethoxycarbonyl)pyridin-1-ium bromide (**16**)

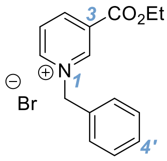

16

Following general procedure B, using benzyl bromide (2.7 mL, 22.5 mmol, 1.5 equiv), ethyl nicotinate (2.0 mL, 5 mmol, 1.0 equiv) and MeCN (30 mL, 0.5 M) for 18 h. Upon completion, the reaction mixture was concentrated in vacuo to give the title compound (4.80 g, quant.) as a beige solid with spectroscopic data in accordance with the literature.[10] **mp** 140-144 °C {no Lit. mp}; **^{1}H NMR** (400 MHz, acetone) δ_H: 1.41 (3H, t, *J* 7.1, $CO_2CH_2CH_3$), 4.48 (2H, q, *J* 7.1, $CO_2CH_2CH_3$), 6.49 (2H, s, CH_2), 7.21 – 7.64 (3H, m, C(3',5')*H* and C(4')*H*), 7.87 (2H, dd, *J* 6.7, 3.0, C(2',6')*H*), 8.42 (1H, dd, *J* 7.8, 6.4, C(5)*H*), 9.12 (1H, app dt, *J* 8.1, 1.5, C(4)*H*), 10.01 (1H, s, C(2)*H*), 10.09 (1H, app dt, *J* 6.2, 1.2, C(6)*H*).

3-(Phenylsulfonyl)pyridine (**S1**)

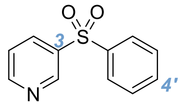

S1

Flame-dried 4 Å molecular sieves, benzenesulfinic acid, sodium salt (3.28 g, 20 mmol, 1.0 equiv), 3-pyridine boronic acid (3.69 g, 30 mmol, 1.5 equiv), copper(II) acetate monohydrate (1.25 g, 6.25 mmol, 1.25 equiv), triethylamine (12.5 mL, 90 mmol, 4.5 equiv), DMSO (100 mL, 0.2 M) and 1,4-dioxane were combined in a flame-dried 500 mL round bottom flask. The flask was sealed, and the reaction was stirred at 65 °C for 16 h. The reaction mixture was allowed to cool to room temperature, diluted with brine (200 mL)

and NH_4OH (12 mL), and extracted with EtOAc:$CHCl_3$ (3:1, 3 × 200 mL). The organic layers were combined, wash with brine (100 mL), dried over $MgSO_4$, filtered and concentrated under reduced pressure. The crude purified by flash column chromatography (0-100% EtOAc in petrol; R_f 0.26 at 60% EtOAc in petrol then 0-8% Et_2O in CH_2Cl_2; R_f 0.4 at 10% Et_2O in CH_2Cl_2) to give the title compound (1.35 g, 31%) as a white solid with spectroscopic data in accordance with the literature.[11] **mp** 100-102 °C {Lit.[11] 120-122 °C}; **^{1}H NMR** (400 MHz, $CDCl_3$) δ_H: 7.45 (1H, ddd, *J* 8.1, 4.9, 0.8, C(5)*H*), 7.50 – 7.58 (2H, m, C(3',5')*H*), 7.58 – 7.69 (1H, m, C(4')*H*), 7.90 – 8.05 (2H, m, C(2',6')*H*), 8.22 (1H, ddd, *J* 8.1, 2.3, 1.7, C(4)*H*), 8.79 (1H, dd, *J* 4.9, 1.6, C(6)*H*), 9.15 (1H, d, *J* 1.8, C(2)*H*).

1-Benzyl-3-(phenylsulfonyl)pyridin-1-ium bromide (**17**)

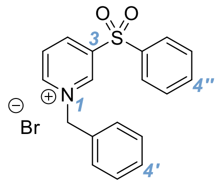

17

Following general procedure B, using benzyl bromide (0.8 mL, 7.0 mmol, 1.5 equiv), 3-(phenylsulfonyl)pyridine **S1** (1.03 g, 4.7 mmol, 1.0 equiv) and MeCN (29 mL, 0.16 M) for 18 h. Upon completion, the reaction mixture was concentrated in vacuo, diluted with Et_2O and filtered to give the title compound (1.64 g, 89%) as a peach solid. **mp** 172-174 °C; **ν_{max}** (solid): 2995 (C-H), 1489, 1450, 1333 (SO_2), 1315, 1176, 1146 (SO_2), 1122, 870, 596; **^{1}H NMR** (500 MHz, DMSO) δ_H: 5.99 (2H, s, C*H*$_2$), 7.40 – 7.51 (3H, m, C(3',5')*H* and C(4')*H*), 7.59 (2H, dd, *J* 7.5, 1.9, C(2',6')*H*), 7.74 (2H, t, *J* 7.8, C(3'',5'')*H*), 7.83 (1H, t, *J* 7.5, C(4'')*H*), 8.14 (2H, dd, *J* 8.4, 1.1, C(2'',6'')*H*), 8.37 (1H, dd, *J* 8.1, 6.2, C(5)*H*), 9.17 (1H, d, *J* 8.6, C(4)*H*), 9.39 (1H, d, *J* 6.1, C(6)*H*), 10.05 (1H, s, C(2)*H*); **^{13}C{^{1}H} NMR** (126 MHz, DMSO) δ_C: 63.9 (CH_2), 128.4 (*C*(2'',6'')H), 129.2 (*C*(2',6')H), 129.3 (*C*(3',5')H), 129.6 (*C*(4')H), 129.9 (*C*(5)H), 130.2 (*C*(3'',5'')H), 133.6 (*C*(1')), 135.4 (*C*(4'')H), 138.3 (*C*(1'')), 141.4 (*C*(3)), 144.5 (*C*(4)H or *C*(6)H), 144.6 (*C*(4)H or *C*(6)H), 149.0 (*C*(6)H); **HRMS** (ESI$^+$) $C_{18}H_{16}NO_2S$ [M−Br]$^+$ found 310.0890, requires 310.0896 (−1.9 ppm).

1-(Naphthalen-2'-ylmethyl)-3-(phenylsulfonyl)pyridin-1-ium bromide (**18**)

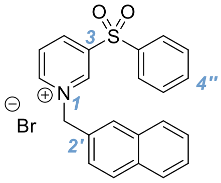

18

Following general procedure B, using 2-(bromomethyl)naphthalene (332 mg, 1.5 mmol, 1.5 equiv), 3-(phenylsulfonyl)pyridine **S1** (219 mg, 1.0 mmol, 1.0 equiv) and MeCN (6.2 mL, 0.16 M) for 72 h gave, the title compound (346 mg, 78%) as a white solid. **mp** 142-144 °C; **ν_{max}** (solid): 2986 (C-H), 2901 (C-H), 1634, 1474, 1329 (SO_2), 1150 (SO_2), 1126, 1080, 831, 766; **^{1}H NMR** (500 MHz, DMSO) δ_H: 6.16 (2H, s, CH_2), 7.51 – 7.62 (2H, m, C(6')*H* and C(7')*H*), 7.66 (1H, d, *J* 8.5, C(3')*H*), 7.73 (2H, t, *J* 7.8, C(3'',5'')*H*), 7.83 (1H, t, *J* 7.4, C(4'')*H*), 7.88 – 7.98 (2H, m, C(5')*H* and C(8')*H*), 8.00 (1H, d, *J* 8.5, C(4')*H*), 8.09 – 8.25 (3H, m, C(1')*H* and C(2'',6'')*H*), 8.36 (1H, dd, *J* 8.0, 6.3, C(5)*H*), 9.17 (1H, d, *J* 8.2, C(4)*H*), 9.43 (1H, d, *J* 6.1, C(6)*H*), 10.09 (1H, s, C(2)*H*); **^{13}C{^{1}H} NMR** (126 MHz, DMSO) δ_C: 64.1 (CH_2), 126.1 (*C*(3')H), 127.0 (ArCH), 127.3 (ArCH), 127.8 (ArCH), 128.1 (ArCH), 128.4 (*C*(2'',6'')H), 129.0 (ArCH), 129.1 (ArCH), 129.9 (*C*(5)H), 130.2 (*C*(3'',5'')H), 130.9 (ArC), 132.7 (ArC), 133.0 (ArC), 135.4 (*C*(4'')H), 138.3 (*C*(1'')), 141.4 (*C*(3)), 144.5 (*C*(4)H), 144.7 (*C*(2)H), 149.1 (*C*(6)H); **HRMS** (ESI$^+$) $C_{22}H_{18}NO_2S$ [M−Br]$^+$ found 360.1042, requires 360.1053 (−3.0 ppm).

1-(4'-Methoxybenzyl)-3-(phenylsulfonyl)pyridin-1-ium chloride (**19**)

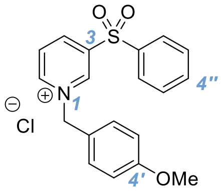

19

Following general procedure B, using 4-methoxybenzyl chloride (0.20 mL, 1.5 mmol, 1.5 equiv), 3-(phenylsulfonyl)pyridine **S1** (219 mg, 1.0 mmol, 1.0 equiv) and MeCN (6.2 mL, 0.16 M) for 72 h gave, after recrystallisation from CH_2Cl_2/Et_2O, the title compound (273 mg, 65%) as a white solid. **mp** 136-138 °C; **ν_{max}** (solid): 3051 (C-H), 2901 (C-H), 1628, 1610, 1516, 1447, 1337, 1252, 1175 (SO_2), 1152, 1112, 1014, 868; **^{1}H NMR** (500 MHz, DMSO) δ_H: 3.76 (3H, s, OCH_3), 5.93 (2H, s, CH_2), 7.00 (2H, d, *J* 8.6, C(3',5')*H*), 7.59 (2H, d, *J* 8.6,

C(2',6')*H*), 7.73 (2H, t, *J* 7.8, C(3'',5'')*H*), 7.83 (1H, t, *J* 7.4, C(4'')*H*), 8.15 (2H, d, *J* 7.5, C(2,6)*H*), 8.34 (1H, dd, *J* 7.9, 6.4, C(5)*H*), 9.13 (1H, d, *J* 8.2, C(4)*H*), 9.40 (1H, d, *J* 6.1, C(6)*H*), 10.04 (1H, s, C(2)*H*); **^{13}C{^{1}H} NMR** (126 MHz, DMSO) δ_C: 55.3 (OCH_3), 63.5 (CH_2), 114.6 (*C*(3',5')H), 125.4 (*C*(1')), 128.4 (*C*(2'',6'')H), 129.8 (*C*(5)H), 130.2 (*C*(3'',5'')H), 131.2 (*C*(2',6')H), 135.4 (*C*(4'')H), 138.3 (*C*(1'')), 141.3 (*C*(3)), 144.3 (*C*(2)H or *C*(4)H), 144.3 (*C*(2)H or *C*(4)H), 148.8 (*C*(6)H), 160.2 (*C*(4)); **HRMS** (ESI$^+$) $C_{19}H_{18}NO_3S$ [M−Cl]$^+$ found 340.0991, requires 340.1002 (−3.2 ppm).

1-(3-fluorobenzyl)-3-(phenylsulfonyl)pyridin-1-ium bromide (**20**)

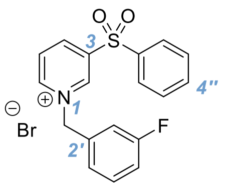

20

Following general procedure B, using 3-fluorobenzyl bromide (0.20 mL, 1.5 mmol, 1.5 equiv), 3-(phenylsulfonyl)pyridine **S1** (219 mg, 1.0 mmol, 1.0 equiv) and MeCN (6.2 mL, 0.16 M) for 48 h gave, after filtration at room temperature and washing with Et_2O, the title compound (280 mg, 67%) as a white solid. **mp** 158-160 °C; **ν_{max}** (solid): 2995, 1630, 1582, 1487, 1450, 1427, 1333, 1317, 1244, 1184, 1153, 1121, 1111, 1084, 854, 797, 758; **^{1}H NMR** (500 MHz, DMSO) δ_H: 5.99 (2H, s, CH_2), 7.29 (1H, app t, *J* 8.4, C(4')*H*), 7.44 (1H, d, *J* 7.6, C(6')*H*), 7.46 – 7.62 (2H, m, C(2')*H* and C(5')*H*), 7.74 (2H, app t, *J* 7.7, C(3'',5'')*H*), 7.83 (1H, app t, *J* 7.3, C(4'')*H*), 8.14 (2H, d, *J* 7.8, C(2'',6'')*H*), 8.28 – 8.46 (1H, m, C(5)*H*), 9.17 (1H, d, *J* 8.2, C(4)*H*), 9.39 (1H, d, *J* 5.9, C(6)*H*), 10.04 (1H, s, C(2)*H*); **^{19}F{^{1}H} NMR** (470 MHz, DMSO) δ_F: -111.9 (s); **^{13}C{^{1}H} NMR** (126 MHz, DMSO) δ_C: 63.1 (CH_2), 116.3 (d, $^2J_{C\text{-}F}$ 22.6, (*C*(2')H), 116.6 (d, $^2J_{C\text{-}F}$ 20.9, *C*(4')H), 125.5 (*C*(6')H), 128.4 (*C*(2'',6'')H), 130.0 (*C*(5)H), 130.3 (*C*(3'',5'')H), 131.4 (d, $^3J_{C\text{-}F}$ 8.3, (*C*(5')H), 135.4 (*C*(4'')H), 135.9 (d, $^3J_{C\text{-}F}$ 8.0, *C*(1')), 138.4 (*C*(1'')), 141.5 (*C*(3)), 144.7 (*C*(4)H), 144.8 (*C*(2)H), 149.1 (*C*(6)H), 162.2 (d, $^1J_{C\text{-}F}$ 244.9, *C*(3')); **HRMS** (ESI+) $C_{18}H_{15}FNO_2S$ [M−Br]$^+$ found 328.0792, requires 328.0802 (−3.0 ppm).

1-Benzylpyridin-1-ium bromide (**21**)

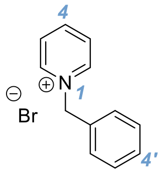

21

Benzyl bromide (2.2 mL, 18.3 mmol, 1.0 equiv) was slowly added to pyridine (1.5 mL, 18.7 mmol, 1.0 equiv) in CH_2Cl_2 (3 mL, 6.1 M) and the reaction was stirred at room temperature for 12 h. The reaction mixture was concentrated in vacuo to give the title compound (2.19 g, 48%) as a cream solid with spectroscopic data in accordance with the literature.[12] **mp** 66-70 °C {Lit.[13] 66-70 °C}; **^{1}H NMR** (400 MHz, $CDCl_3$) δ_H: 6.34 (2H, s, C*H*$_2$), 7.33 – 7.42 (3H, m, C(3',5')*H* and C(4')*H*), 7.63 – 7.72 (2H, m, C(2',6')*H*), 8.02 (2H, t, *J* 7.2, C(3,5)*H*), 8.41 (1H, tt, *J* 7.9, 1.3, C(4)*H*), 9.59 (2H, d, *J* 5.5, C(2,6)*H*).

1-Benzyl-3-chloropyridin-1-ium bromide (**22**)

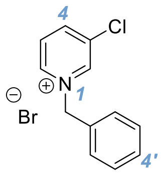

22

Following general procedure B, using benzyl bromide (1.1 mL, 9 mmol, 1.5 equiv), 3-chloropyridine (0.6 mL, 6 mmol, 1.0 equiv) and MeCN (12 mL, 0.5 M) for 18 h gave, the title compound (1.48 g, 87%) as a beige solid. **mp** 126-130 °C; **ν_{max}** (solid): 3003 (C-H), 2930 (C-H), 1620, 1491, 1452, 1206, 1187, 741, 714, 671; **^{1}H NMR** (400 MHz, $CDCl_3$) δ_H: 6.44 (2H, s, C*H*$_2$), 7.29 – 7.42 (3H, m, C(3',5')*H* and C(4')*H*), 7.76 (2H, dd, *J* 6.7, 2.9, C(2',6')*H*), 8.09 (1H, dd, *J* 8.4, 6.1, C(5)*H*), 8.38 (1H, ddd, *J* 8.4, 1.8, 1.1, C(4)*H*), 9.58 – 9.92 (2H, m, C(2)*H* and C(6)*H*); **^{13}C{^{1}H} NMR** (101 MHz, $CDCl_3$) δ_C: 64.2 (CH_2), 129.0 (*C*(5)H), 129.8 (*C*(3',5')H), 130.0 (*C*(2',6')H), 130.3 (*C*(4')H), 132.6 (*C*(1')), 135.8 (*C*(3)), 143.9 (*C*(2)H or *C*(6)H), 144.0 (*C*(2)H or *C*(6)H), 145.2 (*C*(4)H); **HRMS** (ESI$^+$) $C_{12}H_{11}ClN$ [M−Br]$^+$ found 204.0571, requires 204.0575 (−2.0 ppm).

1-Benzyl-3-carbamoylpyridin-1-ium bromide (**23**)

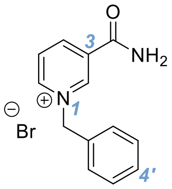

23

Following general procedure B, using benzyl bromide (1.8 mL, 15 mmol, 1.5 equiv), nicotinamide (1.22 g, 10 mmol, 1.0 equiv) and MeCN (20 mL, 0.5 M) for 18 h. The reaction mixture was filtered, washing with Et_2O, to give the title compound (2.7 g, 92%) as a white solid with spectroscopic data in accordance with the literature.[9] **mp** 215-217 °C {Lit.[9] 214-215 °C}; **^{1}H NMR** (400 MHz, D_2O) δ_H: 5.87 (2H, s, C*H*$_2$), 7.48 (5H, s, C(2',6')*H*, C(3',5')*H* and C(4')*H*), 8.16 (1H, dd, *J* 8.0, 6.3, C(5)*H*), 8.87 (1H, app dt, *J* 8.1, 1.4, C(4)*H*), 9.04 (1H, d, *J* 6.2, C(6)*H*), 9.33 (1H, s, C(2)*H*).

3-cyano-1-methylpyridin-1-ium iodide (**24**)

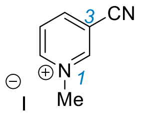

24

Following general procedure B, using methyl iodide (0.9 mL, 15.0 mmol, 1.5 equiv), 3-pyridinecarbonitrile (1.04 g, 10 mmol, 1.0 equiv) and MeCN (20 mL, 0.5 M) for 18 h gave the title compound (376 mg, 15%) as a yellow solid with spectroscopic data in accordance with the literature.[14] **mp** 196-198 °C (decomp.) {No Lit mp}; **^{1}H NMR** (400 MHz, DMSO) δ_H: 4.37 (3H, s, C*H*$_3$), 8.34 (1H, dd, *J* 8.0, 6.4, C(5)*H*), 9.07 (1H, d, *J* 8.2, C(4)*H*), 9.25 (1H, d, *J* 6.2, C(6)*H*), 9.74 (1H, s, C(2)*H*); **^{13}C{^{1}H} NMR** (101 MHz, DMSO) δ_C: 48.6 (*C*H$_3$), 112.1 (*C*(3)), 113.9 (CN), 128.0 (*C*(5)H), 148.1 (*C*(4)H), 149.1 (*C*(6)H), 149.8 (*C*(2)H).

5. Synthesis of catalysis products

Optimisation tables 1 and 2 were carried out according to general procedure C.

Table 3 (probing the effect of the counterion) was carried out according to general procedure D.

N-Benzyl-2-(1'''-benzyl-3'''-cyano-1''',4'''-dihydropyridin-4'''-yl)-2-(*p*-tolyl)acetamide (**6**)

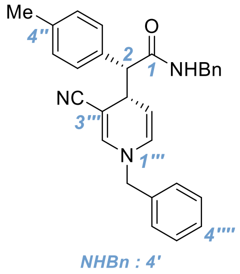

6

Following general procedure D, using 4-nitrophenyl 2-(*p*-tolyl)acetate **1** (81.4 mg, 0.30 mmol, 1.5 equiv), 1-benzyl-3-cyanopyridin-1-ium bromide **2** (55 mg, 0.20 mmol, 1.0 equiv), (*R*)-BTM (10.1 mg, 0.04 mmol, 20 mol%), DABCO (33.6 mg, 0.30 mmol, 1.5 equiv) and PhMe (1.3 mL, 0.15 M) for 24 h at 0 °C, then benzylamine (109 μL, 1.0 mmol, 5.0 equiv) for 24 h at 0 °C, gave the crude product (65%, 90:10 dr). Purification by flash column chromatography (0 to 50% EtOAc in petrol; R_f 0.30 at 40% EtOAc in petrol then 100% Et_2O; R_f 0.70 at 100% Et_2O) gave the title compound (40 mg, 46%, single major diastereoisomer) as a yellow foam. $[\alpha]_D^{20}$ −229.6 (*c* 0.5, $CHCl_3$); **HPLC**: Chiralpak AD-H, (85:15 hexane: IPA, flow rate 1.0 mLmin^{-1}, 211 nm, 30 °C) t_R (minor): 25.5 min, t_R (major): 31.7 min, 91:9 er; ν_{max} (film, cm^{-1}) 3312 (C(O)N-H), 2920 (C-H), 2189 (C≡N), 1670 (C=C-N), 1649 (C=O), 1589, 1510, 1412, 1180, 1121, 1028, 729, 696; **^{1}H NMR** (500 MHz, $CDCl_3$) δ_H: 2.41 (3H, s, C*H*$_3$), 3.71 (1H, d, *J* 4.1, C(2)*H*), 4.06 (2H, s, N(1''')C*H*$_2$Ph), 4.33 – 4.46 (3H, m, C(4''')*H* and NHC*H*$_2$Ph), 4.99 (1H, dd, *J* 8.2, 4.2, C(5''')*H*), 5.47 – 5.92 (2H, m, C(6''')*H* and N*H*), 6.40 (1H, d, *J* 1.4, C(2''')*H*), 6.76 (2H, d, *J* 6.0, C(2'''',6'''')*H*), 7.15 (2H, d, *J* 7.8, C(3'',5'')*H*), 7.18 (2H, d, *J* 7.1, C(2',6')*H*), 7.21 – 7.33 (8H, m, ArC*H*); **^{13}C{^{1}H} NMR** (126 MHz, $CDCl_3$) δ_C: 21.4 (CH_3), 37.1 (*C*(4''')H), 43.8 (NHCH_2Ph), 57.2 (N(1''')CH_2Ph), 57.5 (*C*(2)H), 80.8 (*C*(3''')CN), 104.3 (*C*(5''')H), 121.2 (CN), 126.9 (*C*(2'''')H), 127.5 (*C*(4')H), 127.8 (*C*(2')H), 128.0 (*C*(4'''')H), 128.8 (*C*(3')H), 128.9 (*C*(3'''')H), 129.2 (*C*(3'')H), 129.5 (*C*(6''')H), 130.7 (*C*(2'')H),

133.0 (C(1'')), 136.2 (C(1'''')), 137.2 (C(4'')), 138.3 (C(1')), 144.1 (C(2''')H), 171.7 (C(1)); **HRMS** (ESI+) $C_{29}H_{27}N_3NaO$ $[M+Na]^+$ found 456.2040, requires 456.2046 (−1.3 ppm).

Ethyl (*R*)-1-benzyl-4-((*R*)-2'-(benzylamino)-2'-oxo-1'-phenylethyl)-1,4-dihydropyrid-ine-3-carboxylate (**25**)

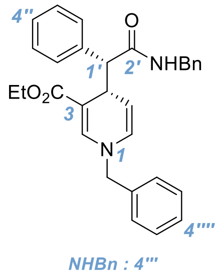

25

Following general procedure D, using 4-nitrophenyl 2-phenylacetate **10** (77.2 mg, 0.30 mmol, 1.5 equiv), 1-benzyl-3-(ethoxycarbonyl)pyridin-1-ium bromide **16** (64.4 mg, 0.20 mmol. 1.0 equiv), (*R*)-BTM (10.1 mg, 0.04 mmol, 20 mol%), DABCO (33.6 mg, 0.3 mmol, 1.5 equiv) and PhMe (1.3 mL, 0.15 M) for 24 h at 0 °C, then benzylamine (109 µL, 1.0 mmol, 5.0 equiv) for 24 h at 0 °C, gave crude product (61%, 90:10 dr). Purification by flash column chromatography (0 to 40% EtOAc in petrol; R_f 0.40 at 40% EtOAc in petrol) gave the title compound (30 mg, 32%, > 95:5 dr) as a yellow gum. **HPLC**: Chiralcel OD-H, (80:20 hexane: IPA, flow rate 1.0 mLmin^{-1}, 211 nm, 30 °C) t_R: 10.2 min and 18.0 min, 50:50 er; ν_{max} (film, cm^{-1}) 3323 (N-H), 3028, 2926 (C-H), 1672 (C(=O)OR), 1651 (C(=O)NH), 1581, 1495, 1452, 1265, 1204, 1163, 1072, 1026, 731, 696; **^{1}H NMR** (500 MHz, $CDCl_3$) δ_H: 1.28 (3H, t, *J* 7.1, $CO_2CH^AH^BCH_3$), 3.96 (1H, d, *J* 3.6, C(1')*H*), 4.06 (2H, s, NCH_2Ph), 4.15 – 4.26 (2H, m, $CO_2CH^AH^BCH_3$), 4.41 (2H, dd, *J* 5.7, 2.0, CONHCH^AH^BPh), 4.49 (1H, app t, *J* 4.2, C(4)*H*), 5.19 (1H, dd, *J* 8.0, 4.8, C(5)*H*), 5.70 (1H, dd, *J* 8.2, 1.1, C(6)*H*), 5.77 (1H, t, *J* 5.4, N*H*), 6.69 (2H, dd, *J* 6.5, 2.9, C(2'''',6'''')*H*), 7.01 (1H, d, *J* 1.6, C(2)*H*), 7.17 – 7.22 (2H, m, C(2''',6''')*H*), 7.22 – 7.33 (11H, m, ArC*H*); **^{13}C{^{1}H} NMR** (126 MHz, $CDCl_3$) δ_C: 14.7 ($CO_2CH^AH^BCH_3$), 36.9 (C(4)H), 43.6 (CONHCH^{A}H^{B}Ph), 56.9 (C(1')H), 57.4 (NCH_2Ph), 59.7 ($CO_2CH^AH^BCH_3$), 98.5 (C(3)), 105.9 (C(5)H), 126.9 (C(2'''',6'''')H), 127.1 (ArCH), 127.4 (ArCH), 127.7 (C(4'''')*H*), 127.8 (2 ArCH), 127.9 (2 ArCH), 128.7 (2 ArCH), 128.9 (2 ArCH), 129.5 (C(6)*H*), 130.9 (C(2'',6'')H), 136.8 (C(1'''')), 137.2 (C(1'')), 138.6 (C(1''')), 143.0 (C(2)H),

168.2 (CO_2Et), 172.6 (CONH); **HRMS** (ESI$^+$) $C_{30}H_{29}N_2O_3$ [M−H]$^+$ found 465.2161, requires 465.2184 (−4.9 ppm).

(*R*)-*N*-Benzyl-2-((*R*)-1'''-benzyl-3'''-(phenylsulfonyl)-1''',4'''-dihydropyridin-4'''-yl)-2-phenylacetamide (**26**)

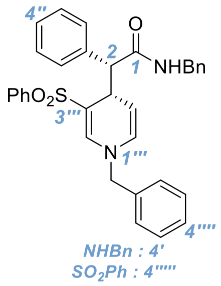

26

Following general procedure D, using 4-nitrophenyl 2-phenylacetate **10** (77 mg, 0.30 mmol, 1.5 equiv), 1-benzyl-3-(phenylsulfonyl)pyridin-1-ium bromide **17** (78.0 mg, 0.20 mmol. 1.0 equiv), (*R*)-BTM (10.1 mg, 0.04 mmol, 20 mol%), DABCO (33.6 mg, 0.3 mmol, 1.5 equiv) and PhMe (1.3 mL, 0.15 M) for 24 h at 0 °C, then benzylamine (109 µL, 1.0 mmol, 5.0 equiv) for 24 h at 0 °C, gave crude product (71%, 90:10 dr). Purification by flash column chromatography (0 to 5% Et_2O in CH_2Cl_2; R_f 0.35 at 5% Et_2O in CH_2Cl_2) gave the title compound (62 mg, 58%, 95:5 dr) as a white foam. $[\alpha]_D^{20}$ −471 (*c* 1.0, $CHCl_3$); **HPLC**: Chiralpak IB, (80:20 hexane: IPA, flow rate 1.0 mLmin^{-1}, 211 nm, 30 °C) t_R (minor): 12.2 min, t_R (major): 14.6 min, 94:6 er; ν_{max} (film, cm^{-1}) 3360 (N-H), 1666 (C=O), 1582, 1510 (C=C), 1495, 1281, 1136 (SO_2), 1086 (SO_2), 723, 688, 594; **^{1}H NMR** (500 MHz, $CDCl_3$) δ_H: 4.01 (2H, s, N(1''')C*H*$_2$Ph), 4.17 (1H, dd, *J* 5.0, 3.1, C(4''')*H*), 4.29 – 4.49 (3H, m, C(2)*H* and NHC*H*$_2$Ph), 5.09 (1H, dd, *J* 7.9, 5.1, C(5''')*H*), 5.56 – 5.76 (2H, m, N*H* and C(6''')*H*), 6.64 (2H, dd, *J* 7.6, 1.6, C(2'''',6'''')*H*), 7.08 (1H, d, *J* 1.2, C(2''')*H*), 7.17 (2H, d, *J* 6.9, C(2',6')*H*), 7.20 – 7.27 (4H, m, C(3'''',5'''')*H* and 2 ArC*H*), 7.26 – 7.30 (4H, m, C(3',5')*H* and 2 ArC*H*), 7.30 – 7.34 (1H, m, C(4'')*H*), 7.34 – 7.41 (2H, m, C(2'',6'')*H*), 7.52 (2H, t, *J* 7.5, C(3''''',5''''')*H*), 7.57 – 7.62 (1H, m, C(4''''')*H*), 7.85 – 7.94 (2H, m, C(2''''',6''''')*H*); **^{13}C{^{1}H} NMR** (126 MHz, $CDCl_3$) δ_C: 36.2 (*C*(4''')H), 43.6 (NHCH$_2$Ph), 57.2 (*C*(2)H), 57.5 (N(1''')CH$_2$Ph), 105.3 (*C*(5''')H), 105.5 (*C*(3''')), 127.0 (*C*(2'''',6'''')H), 127.4 (ArCH), 127.5 (ArCH), 127.5 (*C*(2''''',6''''')H), 127.7 (*C*(2',6')H), 127.9 (2 ArCH), 128.7 (2 ArCH), 128.9 (2 ArCH), 129.2 (*C*(3''''',5''''')H), 129.8 (*C*(6''')H), 131.5 (*C*(2'',6'')H), 132.7 (*C*(4''''')H), 135.9 (*C*(1'''')), 136.2 (*C*(1'')), 138.4 (*C*(1')),

141.0 (C(1''''')), 142.9 (C(2''')H), 172.6 (C(1)=O); **HRMS** (ESI$^+$) $C_{33}H_{30}N_2NaO_3S$ [M+Na]$^+$ found 557.1863, requires 557.1869 (−1.1 ppm).

(R)-N-Benzyl-2-((R)-1'''-(naphthalen-2''''-ylmethyl)-3'''-(phenylsulfonyl)-1''',4'''-dihydropyridin-4'''-yl)-2-phenylacetamide (**27**)

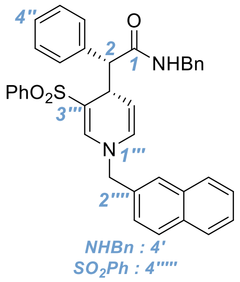

27

Following general procedure D, using 4-nitrophenyl 2-phenylacetate **10** (77.2 mg, 0.30 mmol, 1.5 equiv), 1-(naphthalen-2-ylmethyl)-3-(phenylsulfonyl)pyridin-1-ium bromide **18** (88.1 mg, 0.20 mmol. 1.0 equiv), (R)-BTM (10.1 mg, 0.04 mmol, 20 mol%), DABCO (33.6 mg, 0.3 mmol, 1.5 equiv) and PhMe (1.3 mL, 0.15 M) for 24 h at 0 °C, then benzylamine (109 μL, 1.0 mmol, 5.0 equiv) for 24 h at 0 °C, gave crude product (72%, 90:10 dr). Purification by flash column chromatography (0 to 5.5% Et_2O in CH_2Cl_2; R_f 0.32 at 5% Et_2O in CH_2Cl_2) gave the title compound (70 mg, 60%, 95:5 dr) as a yellow/white foam. **$[\alpha]_D^{20}$** −417 (c 0.5, $CHCl_3$); **HPLC**: Chiralpak IB, (80:20 hexane: IPA, flow rate 1.0 mLmin^{-1}, 211 nm, 30 °C) t_R (major): 16.8 min, t_R (minor): 20.8 min, 94:6 er; **ν_{max}** (film, cm^{-1}) 3343 (C(O)N-H), 3055, 1664, 1584, 1508, 1414, 1279, 1134 (SO_2), 1086, 1043, 816; **^{1}H NMR** (500 MHz, $CDCl_3$) δ_H: 4.16 (2H, s, N(1''')CH^AH^B), 4.18 – 4.24 (1H, m, C(4''')H), 4.37 – 4.45 (3H, m, NCH^AH^BPh and C(2)H), 5.12 (1H, dd, J 7.8, 5.2, C(5''')H), 5.65 – 5.86 (2H, m, C(6''')H and NH), 6.74 (1H, d, J 8.4, C(3'''')H), 7.19 (3H, app d, J 8.7, C(2',6')H and C(2''')H), 7.22 – 7.34 (6H, m, C(3',5')H, C(4')H, C(3'',5'')H and C(4'')H), 7.35 – 7.41 (3H, m, C(2'',6'')H and C(1'''')H), 7.49 – 7.57 (4H, m, C(6'''')H, C(7'''')H and C(3''''',5''''')H), 7.61 (1H, t, J 7.3, C(4''''')H), 7.75 (1H, d, J 8.5, C(4'''')H), 7.76 – 7.80 (1H, m, C(8'''')H), 7.82 – 7.89 (1H, m, C(5'''')H), 7.94 (2H, d, J 7.7, C(2''''',6''''')H); **^{13}C{^{1}H} NMR** (126 MHz, $CDCl_3$) δ_C: 36.2 (C(4''')H), 43.5 (NCH^{A}H^{B}Ph), 57.2 (C(2)H), 57.7 (N(1''')CH^{A}H^B), 105.3 (C(5)H), 105.6 (C(3)), 125.0 (C(3'''')H), 126.4 (ArCH), 126.5 (ArCH), 126.6 (ArCH), 127.4 (ArCH), 127.4 (ArCH), 127.5 (C(2''''',6''''')H), 127.6 (C(2',6')H), 127.8 (2 ArCH and ArCH), 127.9 (ArCH), 128.7

(C(3',5')H), 129.0 (C(4''''')H), 129.2 (C(3''''',5''''')H), 129.7 (C(6''')H), 131.4 (C(2'',6'')H), 132.7 (C(4''''')H), 133.0 (C(4''''a)), 133.2 (C(2'''')), 133.2 (C(8''''a)), 136.1 (C(1'')), 138.3 (C(1')), 141.0 (C(1''''')), 142.8 (C(2''')H), 172.5 (C(1)=O); **HRMS** (ESI+) $C_{37}H_{32}N_2NaO_3S$ [M+Na]+ found 607.2012, requires 607.2026 (−2.3 ppm).

(R)-N-Benzyl-2-((R)-1'''-(4''''-methoxybenzyl)-3'''-(phenylsulfonyl)-1''',4'''-dihydro-pyridin-4'''-yl)-2-phenylacetamide (**28**)

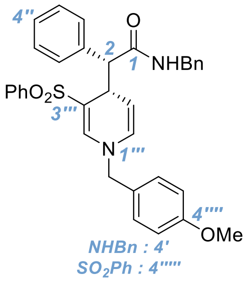

28

Following general procedure D, using 4-nitrophenyl 2-phenylacetate **10** (77.2 mg, 0.30 mmol, 1.5 equiv), 1-(4-methoxybenzyl)-3-(phenylsulfonyl)pyridin-1-ium chloride **19** (84.1 mg, 0.20 mmol. 1.0 equiv), (R)-BTM (10.1 mg, 0.04 mmol, 20 mol%), DABCO (33.6 mg, 0.3 mmol, 1.5 equiv) and PhMe (1.3 mL, 0.15 M) for 24 h at 0 °C, then benzylamine (109 μL, 1.0 mmol, 5.0 equiv) for 24 h at 0 °C, gave crude product (71%, 90:10 dr). Purification by flash column chromatography (0 to 7.5% Et_2O in CH_2Cl_2; R_f 0.33 at 7% Et_2O in CH_2Cl_2) gave the title compound (73 mg, 65%, 94:6 dr) as a yellow/white foam. $[\alpha]_D^{20}$ −423 (c 0.5, $CHCl_3$); **HPLC**: Chiralpak IB, (80:20 hexane: IPA, flow rate 1.0 mLmin^{-1}, 211 nm, 30 °C) t_R (major): 14.8 min, t_R (minor): 17.4 min, 91:9 er; ν_{max} (film, cm^{-1}) 3346 (C(O)N-H), 1664, 1582, 1510, 1414, 1281, 1246, 1134, 1086, 1026; **^{1}H NMR** (400 MHz, $CDCl_3$) δ_H: 3.81 (3H, s, OCH_3), 3.93 (2H, s, N(1''')CH_2Ph), 4.16 (1H, dd, J 5.1, 3.1, C(4''')H), 4.33 – 4.43 (3H, m, C(2)H and CH^AH^BPh), 5.08 (1H, dd, J 7.9, 5.1, C(5''')H), 5.64 (1H, dd, J 7.9, 1.3, C(6''')H), 5.69 (1H, app t, J 5.7, NH), 6.58 (2H, d, J 8.7, C(2'''',6'''')H), 6.76 (2H, d, J 8.7, C(3'''',5'''')H), 7.06 (1H, d, J 1.1, C(2''')H), 7.11 – 7.19 (2H, m, C(2',6')H), 7.21 – 7.34 (6H, m, C(4')H, C(3'',5'')H and C(4')H), 7.34 – 7.40 (2H, m, C(2'',6'')H), 7.51 (2H, t, J 7.4, C(3''''',5''''')H), 7.54 – 7.61 (1H, m, C(4''''')H), 7.84 – 7.94 (2H, m, C(2''''',6''''')H); **^{13}C{^{1}H} NMR** (101 MHz, $CDCl_3$) δ_C: 36.3 (C(4''')H), 43.6 (CH^AH^BPh), 55.4 (OCH_3), 56.9 (N(1)CH_2Ph), 57.2 (C(2)H), 105.1 (C(5''')H), 105.2 (C(3''')), 127.3 (ArCH), 127.4 (ArCH), 127.4 (2 ArCH), 127.6

(C(2',6')H), 127.7 (C(1''''')), 127.9 (2 ArCH), 128.5 (C(2'''',6'''')H), 128.7 (2 ArCH), 129.1 (C(3''''',5''''')H), 129.6 (C(6''')H), 131.5 (C(2'',6'')H), 132.7 (C(4''''')H), 136.2 (C(1'')), 138.4 (C(1')), 141.1 (C(1''''')), 142.7 (C(2''')H), 159.3 (C(4'''')), 172.5 (C(1)=O); **HRMS** (ESI$^+$) $C_{34}H_{32}N_2NaO_4S$ [M+Na]$^+$ found 587.1959, requires 587.1975 (−2.7 ppm).

(*R*)-*N*-Benzyl-2-((*S*)-1'''-(3''''-fluorobenzyl)-3'''-(phenylsulfonyl)-1''',4'''-dihydropyridin-4'''-yl)-2-phenylacetamide (**29**)

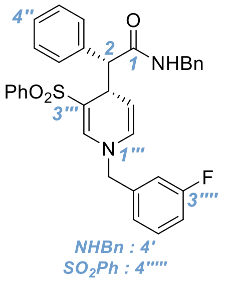

29

Following general procedure D, using 4-nitrophenyl 2-phenylacetate **10** (77.2 mg, 0.30 mmol, 1.5 equiv), 1-(3-fluorobenzyl)-3-(phenylsulfonyl)pyridin-1-ium bromide **20** (81.6 mg, 0.20 mmol. 1.0 equiv), (*R*)-BTM (10.1 mg, 0.04 mmol, 20 mol%), DABCO (33.6 mg, 0.3 mmol, 0.3 mmol, 1.5 equiv) and PhMe (1.3 mL, 0.15 M) for 24 h at 0 °C, then benzylamine (109 μL, 1.0 mmol, 5 equiv) for 24 h at 0 °C, gave crude product (57%, 90:10 dr). Purification by flash column chromatography (0 to 3.5% Et_2O in CH_2Cl_2; R_f 0.38 at 3% Et_2O in CH_2Cl_2 then 10 to 45% EtOAc in petrol; R_f 0.30 at 40% EtOAc in petrol) gave the title compound (34 mg, 31%, > 95:5 dr) as a pale yellow foam. $[\alpha]_D^{20}$ −483 (*c* 0.5, $CHCl_3$); **HPLC**: Chiralcel OD-H, (80:20 hexane: IPA, flow rate 1.0 mLmin^{-1}, 220 nm, 30 °C) t_R (major): 14.5 min, t_R (minor): 18.9 min, 93:7 er; ν_{max} (film, cm^{-1}) 3350 (N-H), 3061, 2963, 1688 (C=O), 1587, 1533, 1447, 1412, 1287, 1211, 1140, 1088, 1043, 1022; **^{1}H NMR** (500 MHz, $CDCl_3$) δ_H: 4.00 (2H, s, N(1''')CH_2Ar), 4.16 (1H, dd, *J* 5.0, 3.1, C(4''')*H*), 4.33 – 4.51 (3H, m, NCH_2Ph and C(2)*H*), 5.11 (1H, dd, *J* 7.9, 5.1, C(5''')*H*), 5.50 – 5.74 (2H, m, N*H* and C(6''')*H*), 6.36 – 6.47 (2H, m, C(2'''')*H* and C(6'''')*H*), 6.95 (1H, td, *J* 8.3, 2.1, C(4'''')*H*), 7.06 (1H, d, *J* 1.3, C(2''')*H*), 7.15 – 7.19 (2H, m, C(2',6')*H*), 7.19 – 7.33 (7H, m, C(3',5')*H*, C(4')*H*, C(3'',5'')*H*, C(4'')*H* and C(5'''')*H*), 7.32 – 7.38 (2H, m, C(2'',6'')*H*), 7.53 (2H, app t, *J* 7.5, C(3''''',5''''')*H*), 7.56 – 7.63 (1H, m, C(4''''')*H*), 7.86 – 7.95 (2H, m, C(2''''',6''''')*H*); **^{19}F{^{1}H} NMR** (470 MHz, $CDCl_3$) δ_F: -111.9 (s); **^{13}C{^{1}H} NMR** (126 MHz, $CDCl_3$) δ_C: 36.1 (C(4''')H), 43.6 (NCH$_2$Ph),

57.0 (C(2)H and N(1''')CH_2Ar), 105.5 (C(5''')H), 106.1 (C(3''')), 114.1 (d, $^2J_{C\text{-}F}$ 21.7, C(2'''')H), 115.1 (d, $^2J_{C\text{-}F}$ 21.2, C(4'''')H), 122.7 (d, $^4J_{C\text{-}F}$ 2.8, C(6'''')H), 127.5 (C(4')H), 127.5 (C(2''''',6''''')H), 127.7 (C(2',6')H and C(4'')H), 127.9 (C(3',5')H or C(3'',5'')H), 128.7 (C(3',5')H or C(3'',5'')H), 129.2 (C(3''''',5''''')H), 129.7 (C(6''')H), 130.6 (d, $^3J_{C\text{-}F}$ 8.3, C(5'''')H), 131.5 (C(2'',6'')H), 132.8 (C(4''''')H), 136.0 (C(1'')), 138.3 (C(1')), 138.4 (d, $^3J_{C\text{-}F}$ 6.9, C(1'''')), 140.8 (C(1''''')), 142.7 (C(2''')H), 163.0 (d, $^1J_{C\text{-}F}$ 247.4, C(3'''')F), 172.5 (C(1)=O); **HRMS** (ESI$^+$) $C_{33}H_{29}FN_2NaO_3S$ [M+Na]$^+$ found 575.1775, requires 575.1781 (-1.0 ppm).

(*R*)-*N*-Benzyl-2-((*R*)-1'''-benzyl-3'''-(phenylsulfonyl)-1''',4'''-dihydropyridin-4'''-yl)-2-(naphthalen-2''-yl)acetamide (**30**)

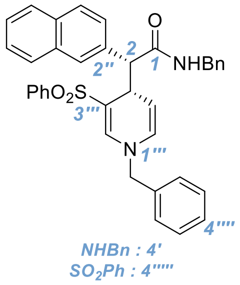

30

Following general procedure D, using 4-nitrophenyl 2-(naphthalen-2-yl)acetate **11** (92 mg, 0.30 mmol, 1.5 equiv), 1-benzyl-3-(phenylsulfonyl)pyridin-1-ium bromide **17** (78.0 mg, 0.20 mmol. 1.0 equiv), (*R*)-BTM (10.1 mg, 0.04 mmol, 20 mol%), DABCO (33.6 mg, 0.3 mmol, 1.5 equiv) and PhMe (1.3 mL, 0.15 M) for 24 h at 0 °C, then benzylamine (109 µL, 1.0 mmol, 5.0 equiv) for 24 h at 0 °C, gave crude product (71%, 95:5 dr). Purification by flash column chromatography (0 to 4.5% Et_2O in CH_2Cl_2; R_f 0.31 at 4% Et_2O in CH_2Cl_2) gave the title compound (82 mg, 70%, > 95:5 dr) as a pale yellow solid. **mp** 70-74 °C; $[\alpha]_D^{20}$ −457 (*c* 1.0, $CHCl_3$); **HPLC**: Chiralpak AS-H, (80:20 hexane: IPA, flow rate 1.0 mLmin^{-1}, 211 nm, 30 °C) t_R (minor): 22.4 min, t_R (major): 40.7 min, 90:10 er; ν_{max} (solid, cm^{-1}) 3329 (N-H), 3059, 1666, 1659, 1584, 1506, 1418, 1281, 1217, 1136 (SO_2), 1085, 721, 688; **^{1}H NMR** (500 MHz, $CDCl_3$) δ_H: 3.91 (2H, s, N(1)CH_2Ph), 4.25 (1H, dd, *J* 5.0, 2.9, C(4''')*H*), 4.33 – 4.50 (2H, m, NHCH^AH^BPh), 4.59 (1H, d, *J* 2.9, C(2)*H*), 5.20 (1H, dd, *J* 7.9, 5.1, C(5''')*H*), 5.63 (1H, dd, *J* 8.0, 1.3, C(6''')*H*), 5.70 (1H, app t, *J* 5.8, N*H*), 6.29 (2H, d, *J* 7.3, C(2'''',6'''')*H*), 6.74 (2H, t, *J* 7.8, C(3'''',5'''')*H*), 6.99 – 7.08 (2H, m, C(4'''')*H* and C(2''')*H*), 7.14 – 7.18 (2H, m, C(2',6')*H*), 7.20 – 7.29 (3H, m, C(3',5')*H* and C(4')*H*), 7.47 – 7.57 (5H, m, C(1'')*H*, C(3'')*H*, C(3''''',5''''')*H*

and 1 ArC*H*), 7.58 – 7.63 (1H, m, C(4''''')*H*), 7.73 (1H, d, *J* 8.5, C(4'')*H*), 7.81 – 7.86 (2H, m, 2 ArC*H*), 7.88 (1H, d, *J* 7.7, 1 ArC*H*), 7.91 – 7.95 (2H, m, C(2''''',6''''')*H*); **^{13}C{^{1}H} NMR** (126 MHz, $CDCl_3$) δ_C: 36.3 (*C*(4''')H), 43.6 (NHCH^{A}H^{B}Ph), 57.2 (*C*(2)H), 57.4 (N(1)*C*H_2Ph), 105.4 (*C*(5''')H), 105.5 (*C*(3''')H), 126.1 (2 ArCH), 126.6 (*C*(2'''',6'''')H), 127.2 (*C*(4'')H), 127.5 (1 ArCH), 127.5 (*C*(2''''',6''''')H), 127.7 (2 ArCH), 127.7 (1 ArCH), 127.8 (1 ArCH), 128.4 (1 ArCH), 128.6 (*C*(3'''',5'''')H), 128.7 (2 ArCH), 129.2 (3 ArCH), 129.9 (*C*(6''')H), 130.7 (1 ArCH), 132.8 (*C*(4''''')H), 132.8 (1 ArC), 133.2 (1 ArC), 133.9 (*C*(1'')), 135.5 (*C*(1'''')), 138.3 (*C*(1')), 140.9 (*C*(1''''')), 142.9 (*C*(2''')H), 172.6 (*C*(1)=O); **HRMS** (ESI$^+$) $C_{37}H_{32}N_2NaO_3S$ $[M+Na]^+$ found 607.2023, requires 607.2026 (−0.5 ppm).

(*R*)-*N*-Benzyl-2-((*R*)-1'''-benzyl-3'''-(phenylsulfonyl)-1''',4'''-dihydropyridin-4'''-yl)-2-(4''-tolyl)acetamide (**31**)

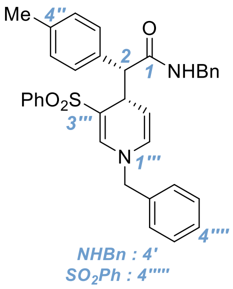

31

Following general procedure D, using 4-nitrophenyl 2-(*p*-tolyl)acetate **1** (81.4 mg, 0.30 mmol, 1.5 equiv), 1-benzyl-3-(phenylsulfonyl)pyridin-1-ium bromide **17** (78.0 mg, 0.20 mmol. 1.0 equiv), (*R*)-BTM (10.1 mg, 0.04 mmol, 20 mol%), DABCO (33.6 mg, 0.3 mmol, 1.5 equiv) and PhMe (1.3 mL, 0.15 M) for 24 h at 0 °C, then benzylamine (109 μL, 1.0 mmol, 5.0 equiv) for 24 h at 0 °C, gave crude product (65%, 80:20 dr). Purification by flash column chromatography (0 to 5.5% Et_2O in CH_2Cl_2; R_f 0.40 at 5% Et_2O in CH_2Cl_2) gave the title compound (59 mg, 54%, 95:5 dr) as a pale yellow solid. **mp** 160-162 °C; **$[\alpha]_D^{20}$** −449 (*c* 1.0, $CHCl_3$); **HPLC**: Chiralpak IB, (85:15 hexane: IPA, flow rate 1.0 mLmin^{-1}, 220 nm, 30 °C) t_R (major): 17.6 min, t_R (minor): 20.3 min, 98:2 er; **ν_{max}** (solid, cm^{-1}) 3346 (CON-H), 2999 (C-H), 1661 (C=O), 1584, 1543, 1419, 1271, 1134 (SO_2), 1084, 723, 590; **^{1}H NMR** (500 MHz, $CDCl_3$) δ_H: 2.40 (3H, s, C*H*$_3$), 4.04 (2H, s, NC*H*$_2$Ph), 4.16 (1H, dd, *J* 4.8, 3.2, C(4''')*H*), 4.34 – 4.43 (3H, m, C(2)H and CONHC*H*^{A}*H*^{B}Ph), 5.07 (1H, dd, *J* 7.9, 5.1, C(5''')*H*), 5.60 – 5.73 (2H, m, N*H* and C(6''')*H*), 6.68 (2H, d, *J* 6.6, C(2'''',6'''')*H*), 7.08 (1H, s, C(2''')*H*),

7.10 (2H, d, *J* 7.9, C(3'',5'')*H*), 7.17 (2H, d, *J* 7.1, C(2',6')*H*), 7.20 – 7.33 (8H, m, C(4')*H*, C(2'',6'')*H*, C(3'''',5'''')*H* C(4'''')*H* and C(3',5')*H*), 7.52 (2H, t, *J* 7.6, C(3''''',5''''')*H*), 7.58 (1H, t, *J* 7.4, C(4''''')*H*), 7.82 – 7.94 (2H, m, C(2''''',6''''')*H*); **^{13}C{^{1}H} NMR** (126 MHz, $CDCl_3$) δ_C: 21.5 (CH_3), 36.1 (C(4''')*H*), 43.6 (CONHCH^{A}H^{B}Ph), 56.8 (*C*(2)H), 57.4 (NCH_2Ph), 105.3 (*C*(5''')H), 105.7 (*C*(3''')), 127.1 (*C*(2'''',6'''')H), 127.4 (*C*(4')H), 127.5 (*C*(2''''',6''''')H), 127.7 (*C*(2',6')H), 127.9 (*C*(4'''')H), 128.7 (*C*(3'',5'')H and 2 ArCH), 128.8 (2 ArCH), 129.2 (*C*(3''''',5''''')H), 129.8 (*C*(6''')H), 131.4 (*C*(2'',6'')H), 132.7 (*C*(4''''')H), 133.2 (*C*(1'')), 135.9 (*C*(1'''')), 136.8 (*C*(4'')), 138.4 (*C*(1')), 141.0 (*C*(1''''')), 142.8 (*C*(2''')H), 172.8 (*C*=O); **HRMS** (ESI$^+$) $C_{34}H_{32}N_2NaO_3S$ $[M+Na]^+$ found 571.2019, requires 571.2026 (−1.2 ppm).

(*R*)-*N*-Benzyl-2-((*R*)-1'''-benzyl-3'''-(phenylsulfonyl)-1''',4'''-dihydropyridin-4'''-yl)-2-(4''-methoxyphenyl)acetamide (**32**)

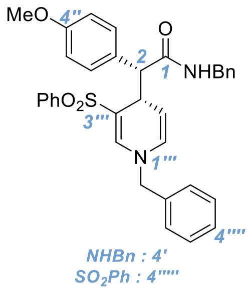

32

Following general procedure D, using 4-nitrophenyl 2-(4-methoxyphenyl)acetate **12** (86.2 mg, 0.30 mmol, 1.5 equiv), 1-benzyl-3-(phenylsulfonyl)pyridin-1-ium bromide **17** (78.0 mg, 0.20 mmol. 1.0 equiv), (*R*)-BTM (10.1 mg, 0.04 mmol, 20 mol%), DABCO (33.6 mg, 0.3 mmol, 1.5 equiv) and PhMe (1.3 mL, 0.15 M) for 24 h at 0 °C, then benzylamine (109 µL, 1.0 mmol, 5.0 equiv) for 24 h at 0 °C, gave crude product (73%, 85:15 dr). Purification by flash column chromatography (0 to 5.5% Et_2O in CH_2Cl_2; R_f 0.36 at 6% Et_2O in CH_2Cl_2) gave the title compound (33 mg, 29%, > 95:5 dr) as a beige foam. $[\alpha]_D^{20}$ −200 (*c* 0.5, $CHCl_3$); **HPLC**: Chiralpak IB, (90:10 hexane: IPA, flow rate 0.5 mLmin^{-1}, 211 nm, 30 °C) t_R (major): 79.4 min, t_R (minor): 87.9 min, 98:2 er; ν_{max} (film, cm^{-1}) 3348 (CON-H), 2959, 2932, 1668, 1653, 1584, 1508, 1452, 1418, 1283, 1246, 1176, 1136 (SO_2), 1086, 1028; **^{1}H NMR** (500 MHz, $CDCl_3$) δ_H: 3.84 (3H, s, OCH_3), 4.06 (2H, s, N(1''')CH_2), 4.13 – 4.17 (1H, m, C(4''')*H*), 4.37 (1H, d, *J* 3.1, C(2)*H*), 4.39 (2H, d, *J* 5.8, NCH_2Ph), 5.06 (1H, dd, *J* 7.9, 5.0, C(5''')*H*), 5.59 – 5.71 (2H, m, N*H* and C(6''')*H*), 6.60 – 6.68 (2H, m, C(2'''',6'''')*H*), 6.82

(2H, d, *J* 8.5, C(3'',5'')*H*), 7.09 (1H, s, C(2''')*H*), 7.17 (2H, d, *J* 7.4, C(2',6')*H*), 7.20 – 7.34 (8H, m, C(3',5')*H*, C(4')*H*, C(2'',6'')*H*, C(3'''',5'''')*H* and C(4'''')*H*), 7.52 (2H, t, *J* 7.6, C(3''''',5''''')*H*), 7.59 (1H, t, *J* 7.4, C(4''''')*H*), 7.90 (2H, d, *J* 7.7, C(2''''',6''''') *H*); **^{13}C{^{1}H} NMR** (126 MHz, $CDCl_3$) δ_C: 36.2 (*C*(4''')H), 43.6 (NCH_2Ph), 55.3 (OCH_3), 56.3 (*C*(2)H), 57.6 (N(1''')CH_2Ph), 105.3 (*C*(5''')H), 105.6 (*C*(3''')), 113.5 (*C*(3'',5'')H), 127.0 (*C*(2'''',6'''')H), 127.5 (*C*(4')H), 127.5 (2 ArCH), 127.7 (2 ArCH), 128.0 (*C*(4'''')H), 128.2 (*C*(1'')), 128.7 (2 ArCH), 128.8 (2 ArCH), 129.2 (*C*(3''''',5''''')H), 129.8 (*C*(6''')H), 132.6 (*C*(2'',6'')H), 132.8 (*C*(4''''')H), 136.0 (*C*(1'''')), 138.4 (*C*(1')), 141.0 (*C*(1''''')), 142.9 (*C*(2''')H), 159.0 (*C*(4'')), 172.9 (*C*(1)=O); **HRMS** (ESI^+) $C_{34}H_{32}N_2NaO_4S$ $[M+Na]^+$ found 587.1965, requires 587.1975 (−1.7 ppm).

(*R*)-*N*-Benzyl-2-((*R*)-1'''-benzyl-3'''-(phenylsulfonyl)-1''',4'''-dihydropyridin-4'''-yl)-2-(3'',4''-dimethoxyphenyl)acetamide (**33**)

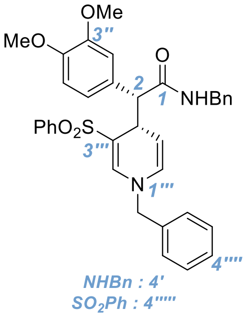

33

Following general procedure D, using 4-nitrophenyl 2-(3,4-dimethoxyphenyl)acetate **13** (95 mg, 0.30 mmol, 1.5 equiv), 1-benzyl-3-(phenylsulfonyl)pyridin-1-ium bromide **17** (78.0 mg, 0.20 mmol. 1.0 equiv), (*R*)-BTM (10.1 mg, 0.04 mmol, 20 mol%), DABCO (33.6 mg, 0.3 mmol, 1.5 equiv) and PhMe (1.3 mL, 0.15 M) for 24 h at 0 °C, then benzylamine (109 μL, 1.0 mmol, 5.0 equiv) for 24 h at 0 °C, gave crude product (65%, 90:10 dr). Purification by flash column chromatography (0 to 8.5% Et_2O in CH_2Cl_2; R_f 0.33 at 8% Et_2O in CH_2Cl_2) gave the title compound (65 mg, 55%, > 95:5 dr) as a white solid. **mp** 122-124 °C; $[\alpha]_D^{20}$ −470 (*c* 1.0, $CHCl_3$); **HPLC**: Chiralpak IA, (80:20 hexane: IPA, flow rate 1.0 mLmin^{-1}, 211 nm, 30 °C) t_R (major): 31.2 min, t_R (minor): 44.3 min, 96:4 er; ν_{max} (solid, cm^{-1}) 3372 (C(O)N-H), 3063, 2924 (C-H), 1666 (C=O), 1582, 1516, 1454, 1422, 1273, 1260, 1234, 1155, 1132, 1086, 1022, 772; **^{1}H NMR** (500 MHz, $CDCl_3$) δ_H: 3.68 (3H, s, C(3'')OC*H*$_3$), 3.90 (3H, s, C(4'')OC*H*$_3$), 4.08 (2H, s, N(1''')C*H*$_2$Ph), 4.15 (1H, dd, *J* 4.9, 3.2,

C(4''')*H*), 4.32 (1H, dd, *J* 14.9, 5.6, NC*H*^A^H^B^Ph), 4.38 (1H, d, *J* 3.1, C(2)*H*), 4.46 (1H, dd, *J* 14.9, 6.2, NCH^A^*H*^B^Ph), 5.10 (1H, dd, *J* 7.9, 5.0, C(5''')*H*), 5.67 (1H, dd, *J* 8.0, 1.2, C(6''')*H*), 5.77 (1H, app t, *J* 5.7, N*H*), 6.66 (2H, dd, *J* 6.5, 2.8, C(2'''',6'''')*H*), 6.78 (1H, d, *J* 8.2, C(5'')*H*), 6.87 (1H, d, *J* 2.0, C(2'')*H*), 6.95 (1H, dd, *J* 8.2, 2.0, C(6'')*H*), 7.10 (1H, d, *J* 1.4, C(2''')*H*), 7.13 – 7.20 (2H, m, C(2',6')*H*), 7.21 – 7.32 (6H, m, C(3',5')*H*, C(4')*H*, C(3'''',5'''')*H* and C(4'''')*H*), 7.52 (2H, t, *J* 7.5, C(3''''',5''''')*H*), 7.56 – 7.62 (1H, m, C(4''''')*H*), 7.80 – 7.93 (2H, m, C(2''''',6''''')*H*); **^{13}C{^{1}H} NMR** (126 MHz, $CDCl_3$) δ_C: 36.2 (*C*(4''')H), 43.5 (NCH^AH^BPh), 55.8 (OCH_3), 55.8 (OCH_3), 56.7 (*C*(2)H), 57.6 (N(1''')CH_2Ph), 105.5 (*C*(5''')H), 105.7 (*C*(3''')), 110.5 (*C*(5'')H), 114.2 (*C*(2'')H), 124.1 (*C*(6'')H), 126.8 (*C*(2'''',6'''')H), 127.4 (*C*(2''''',6''''')H), 127.5 (*C*(4')H), 127.7 (*C*(2',6')H), 128.0 (*C*(4'''')H), 128.6 (*C*(1'')), 128.7 (*C*(3',5')H or *C*(3'''',5'''')H), 128.9 (*C*(3',5')H or *C*(3'''',5'''')H), 129.2 (*C*(3''''',5''''')H), 129.7 (*C*(6''')H), 132.7 (*C*(4''''')H), 135.9 (*C*(1'''')), 138.5 (*C*(1')), 140.9 (*C*(1''''')), 142.9 (*C*(2''')H), 148.1 (*C*(3'')OCH_3), 148.4 (*C*(4'')OCH_3), 172.7 (*C*(1)=O); **HRMS** (ESI$^+$) $C_{35}H_{34}N_2NaO_5S$ $[M+Na]^+$ found 617.2071, requires 617.2081 (−1.6 ppm).

(*R*)-*N*-Benzyl-2-((*R*)-1'''-benzyl-3'''-(phenylsulfonyl)-1''',4'''-dihydropyridin-4'''-yl)-2-(thiophen-3''-yl)acetamide (**34**)

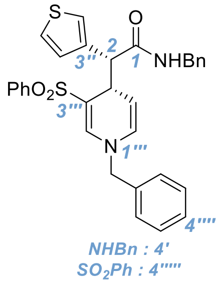

34

Following general procedure D, using 4-nitrophenyl 2-(thiophen-3-yl)acetate **14** (79 mg, 0.30 mmol, 1.5 equiv), 1-benzyl-3-(phenylsulfonyl)pyridin-1-ium bromide **17** (78.0 mg, 0.20 mmol. 1.0 equiv), (*R*)-BTM (10.1 mg, 0.04 mmol, 20 mol%), DABCO (33.6 mg, 0.3 mmol, 1.5 equiv) and PhMe (1.3 mL, 0.15 M) for 24 h at 0 °C, then benzylamine (109 µL, 1.0 mmol, 5.0 equiv) for 24 h at 0 °C, gave crude product (57%, 90:10 dr). Purification by flash column chromatography (0 to 5% Et_2O in CH_2Cl_2; R_f 0.35 at 5% Et_2O in CH_2Cl_2) gave the title compound (54 mg, 50%, 93:7 dr) as a white foam. **$[\alpha]_D^{20}$** −399 (*c* 1.0, $CHCl_3$); **HPLC**: Chiralpak IB, (80:20 hexane: IPA, flow rate 1.0 mLmin^{-1}, 211 nm, 30 °C) t_R (major):

15.4 min, t_R (minor): 19.4 min, 89:11 er; ν_{max} (film, cm^{-1}) 3362 (N-H), 1666 (C=O), 1585, 1526 (ArC=C), 1450, 1418, 1283, 1136 (SO_2); **^{1}H NMR** (500 MHz, $CDCl_3$) δ_H: 3.93 – 4.21 (3H, m, N(1''')CH_2Ph and C(4''')*H*), 4.40 (2H, dd, *J* 5.8, 2.2, NHCH^AH^BPh), 4.53 (1H, d, *J* 3.3, C(2)*H*), 5.01 (1H, dd, *J* 7.9, 5.0, C(5''')*H*), 5.72 (1H, dd, *J* 8.0, 1.3, C(6''')*H*), 5.83 (1H, app t, *J* 5.6, N*H*), 6.84 (2H, dd, *J* 7.1, 2.2, C(2'''',6'''')*H*), 7.07 (1H, dd, *J* 4.9, 1.2, C(4'')*H*), 7.12 (1H, d, *J* 1.4, C(2''')*H*), 7.15 – 7.21 (3H, m, C(2',6')*H* and C(5'')*H*), 7.22 – 7.25 (1H, m, C(4')*H*), 7.27 – 7.35 (6H, m, C(3',5')*H*, C(2'')*H*, C(3'''',5'''')*H* and C(4'''')*H*), 7.52 (2H, t, *J* 7.6, C(3''''',5''''')*H*), 7.55 – 7.62 (1H, m, C(4''''')*H*), 7.85 – 7.91 (2H, m, C(2''''',6''''')*H*); **^{13}C{^{1}H} NMR** (126 MHz, $CDCl_3$) δ_C: 36.1 (*C*(4''')H), 43.6 (NHCH^{A}H^{B}Ph), 53.0 (*C*(2)H), 57.6 (N(1)CH$_2$Ph), 105.1 (*C*(5''')H), 106.0 (*C*(3''')), 124.7 (*C*(5'')H), 125.8 (*C*(2'')H), 127.2 (*C*(2'''',6'''')H), 127.5 (*C*(2''''',6''''')H), 127.6 (*C*(2',6')H), 128.1 (*C*(4')H), 128.8 (2 ArCH), 129.0 (2 ArCH), 129.2 (2 ArCH), 129.8 (*C*(4'')H or *C*(6''')H), 129.8 (*C*(4'')H or *C*(6''')H), 132.8 (*C*(4''''')H), 136.0 (*C*(1'''')), 136.4 (*C*(3'')), 138.4 (*C*(1')), 140.9 (*C*(1''''')), 142.6 (*C*(2''')H), 171.9 (*C*(1)=O); **HRMS** (ESI$^+$) $C_{31}H_{28}N_2NaO_3S_2$ [M+Na]$^+$ found 563.1425, requires 563.1434 (−1.6 ppm).

(*R*)-*N*-Benzyl-2-((*R*)-1'''-benzyl-3'''-(phenylsulfonyl)-1''',4'''-dihydropyridin-4'''-yl)-2-(4''-(trifluoromethyl)phenyl)acetamide (**35**)

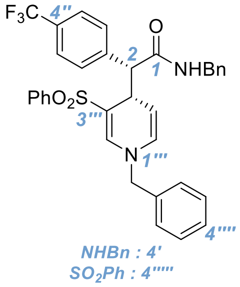

35

Following general procedure D, using 4-nitrophenyl 2-(4-(trifluoromethyl)phenyl)acetate **15** (98 mg, 0.30 mmol, 1.5 equiv), 1-benzyl-3-(phenylsulfonyl)pyridin-1-ium bromide **17** (78.0 mg, 0.20 mmol. 1.0 equiv), (*R*)-BTM (10.1 mg, 0.04 mmol, 20 mol%), DABCO (33.6 mg, 0.3 mmol, 1.5 equiv) and PhMe (1.3 mL, 0.15 M) for 24 h at 0 °C, then benzylamine (109 μL, 1.0 mmol, 5.0 equiv) for 24 h at 0 °C, gave crude product (68%, 90:10 dr). Purification by flash column chromatography (0 to 3.5% Et_2O in CH_2Cl_2; R_f 0.32 at 3% Et_2O in CH_2Cl_2) gave the title compound (59 mg, 49%, > 95:5 dr) as a pale yellow solid. **mp** 62-64 (decomp) then 160-162 °C; **HPLC**: Chiralpak AS-H, (85:15 hexane: IPA,

flow rate 1.0 mLmin^{-1}, 211 nm, 30 °C) t_R (minor): 13.1 min, t_R (major): 30.0 min, 52:48 er; ν_{max} (solid, cm^{-1}) 3343 (N-H), 1666 (C=O), 1582, 1531, 1418, 1323 (SO_2), 1136 (SO_2), 1111 (C-F), 1086, 1067, 723, 688, 590; **^{1}H NMR** (500 MHz, $CDCl_3$) δ_H: 4.00 (2H, s, N(1)CH_2Ph), 4.13 (1H, dd, *J* 5.0, 3.0, C(4''')*H*), 4.31 – 4.44 (2H, m, NHCH^AH^BPh), 4.45 (1H, d, *J* 3.0, C(2)*H*), 5.11 (1H, dd, *J* 7.9, 5.1, C(5''')*H*), 5.55 – 5.77 (2H, m, N*H* and C(6''')*H*), 6.69 (2H, dd, *J* 7.8, 1.5, C(2'''',6'''')*H*), 7.09 (1H, d, *J* 1.3, C(2''')*H*), 7.16 – 7.20 (2H, m, C(2',6')*H*), 7.22 – 7.34 (6H, m, C(3',5')*H*, C(4')*H*, C(3'''',5'''')*H* and C(4'''')*H*), 7.47 – 7.57 (6H, m, C(2'',6'')*H*, C(3'',5'')*H* and C(3''''',5''''')*H*), 7.58 – 7.64 (1H, m, C(4''''')*H*), 7.88 (2H, dd, *J* 8.3, 1.2, C(2''''',6''''')*H*); **^{19}F{^{1}H} NMR** (471 MHz, $CDCl_3$) δ_F: -62.1; **^{13}C{^{1}H} NMR** (126 MHz, $CDCl_3$) δ_C: 36.7 (*C*(4''')H), 43.7 (NHCH^{A}H^{B}Ph), 56.9 (*C*(2)H), 57.5 (N(1)CH_2Ph), 104.8 (*C*(5''')H), 105.3 (*C*(3''')), 124.4 (q, $^1J_{C\text{-}F}$ 272.1, CF_3), 124.7 (app d, $^3J_{C\text{-}F}$ 3.6, *C*(3'',5'')H), 127.2 (*C*(2'''',6'''')H), 127.5 (*C*(2''''',6''''')H), 127.6 (*C*(4')H), 127.7 (*C*(2',6')H), 128.3 (*C*(4'''')H), 128.8 (*C*(3',5')H or *C*(3'''',5'''')H), 129.0 (*C*(3',5')H or *C*(3'''',5'''')H), 129.3 (*C*(3''''',5''''')H), 129.5 (app d, $^2J_{C\text{-}F}$ 32.3, *C*(4'')), 129.9 (*C*(6''')H), 131.7 (*C*(2'',6'')H), 132.9 (*C*(4''''')H), 135.4 (*C*(1'''')), 138.1 (*C*(1')), 140.4 (*C*(1'')), 140.7 (*C*(1''''')), 142.8 (*C*(2''')H), 171.7 (*C*(1)=O); **HRMS** (ESI$^-$) $C_{34}H_{28}F_3N_2O_3S$ [M−H]$^-$ found 601.1787, requires 601.1778 (+1.4 ppm).

(*R*)-2-((*S*)-1'''-Benzyl-3'''-(phenylsulfonyl)-1''',4'''-dihydropyridin-4'''-yl)-2-phenyl-1-(pyrrolidin-1'-yl)ethan-1-one (**36**)

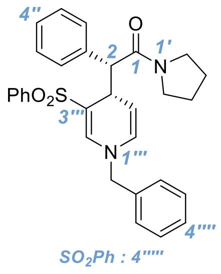

36

Following general procedure D, using 4-nitrophenyl 2-phenylacetate **10** (77 mg, 0.30 mmol, 1.5 equiv), 1-benzyl-3-(phenylsulfonyl)pyridin-1-ium bromide **17** (78.0 mg, 0.20 mmol. 1.0 equiv), (*R*)-BTM (10.1 mg, 0.04 mmol, 20 mol%), DABCO (33.6 mg, 0.3 mmol, 1.5 equiv) and PhMe (1.3 mL, 0.15 M) for 24 h at 0 °C, then pyrrolidine (84 µL, 1.0 mmol, 5.0 equiv) for 24 h at 0 °C, gave crude product (67%, 90:10 dr). Purification by flash column chromatography (0 to 15% Et_2O in CH_2Cl_2; R_f 0.27 at 10% Et_2O in CH_2Cl_2)

gave the title compound (62 mg, 62%, 95:5 dr) as a white foam. **$[\alpha]^{20}_{D}$** −614 (*c* 1.0, $CHCl_3$); **HPLC**: Chiralpak AS-H, (80:20 hexane: IPA, flow rate 1.0 mLmin^{-1}, 211 nm, 30 °C) t_R (minor): 11.1 min, t_R (major): 18.2 min, 93:7 er; **ν_{max}** (film, cm^{-1}) 2872 (C-H), 1668, 1627, 1579, 1418, 1288, 1136 (SO_2), 1086, 1022, 876; **^{1}H NMR** (500 MHz, $CDCl_3$) δ_H: 1.59 – 1.73 (2H, m, C(3')H^AH^B and C(4')H^AH^B), 1.75 – 1.90 (2H, m, C(3')H^AH^B and C(4')H^AH^B), 2.72 – 2.80 (1H, m, C(2')H^AH^B), 3.31 – 3.44 (2H, m, C(2')H^AH^B and C(5')H^AH^B), 3.47 – 3.55 (1H, m, C(5')H^AH^B), 3.92 – 4.06 (3H, m, N(1''')CH_2Ph and C(4''')*H*), 4.45 (1H, d, *J* 2.2, C(2)*H*), 5.35 (1H, dd, *J* 7.9, 5.2, C(5''')*H*), 5.54 (1H, dd, *J* 8.0, 1.3, C(6''')*H*), 6.50 – 6.66 (2H, m, C(2'''',6'''')*H*), 7.14 (1H, d, *J* 1.1, C(2''')*H*), 7.18 – 7.25 (3H, m, C(3'''',5'''')*H* and C(4'''')*H*), 7.25 – 7.33 (5H, m, C(2'',6'')*H*, C(3'',5'')*H* and C(4'')*H*), 7.51 (2H, t, *J* 7.5, C(3''''',5''''')*H*), 7.54 – 7.63 (1H, m, C(4''''')*H*), 7.81 – 7.94 (2H, m, C(2''''',6''''')*H*); **^{13}C{^{1}H} NMR** (126 MHz, $CDCl_3$) δ_C: 24.2 (*C*(3')H^AH^B or *C*(4')H^AH^B), 26.1 (*C*(3')H^AH^B or *C*(4')H^AH^B), 36.6 (*C*(4''')H), 45.9 (*C*(5')H^AH^B), 46.7 (*C*(2')H^AH^B), 56.2 (*C*(2)H), 57.4 (N(1''')CH_2Ph), 105.0 (*C*(3''')), 106.9 (*C*(5''')H), 126.9 (*C*(4'')H), 127.0 (*C*(2'''',6'''')H), 127.3 (*C*(2''''',6''''')H), 127.8 (*C*(3'',5'')H or *C*(3'''',5'''')H), 127.8 (*C*(4'''')H), 128.9 (*C*(3'',5'')H or *C*(3'''',5'''')H), 129.1 (*C*(6''')H), 129.2 (*C*(3''''',5''''')H), 130.9 (*C*(2'',6'')H), 132.6 (*C*(4''''')H), 135.9 (*C*(1'')), 136.0 (*C*(1'''')), 141.5 (*C*(1''''')), 143.3 (*C*(2)H), 171.8 (*C*(1)=O); **HRMS** (ESI$^+$) $C_{30}H_{30}N_2NaO_3S$ $[M+Na]^+$ found 521.1864, requires 521.1869 (−1.0 ppm).

(*S*)-2-(1'''-Benzyl-5'''-(phenylsulfonyl)-1''',2'''-dihydropyridin-2'''-yl)-2-phenyl-1-(pyrrolidin-1'-yl)ethan-1-one (**S2**)

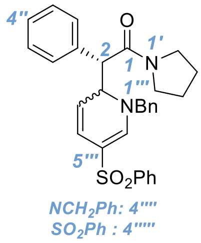

S2

The reaction for also gave product **S2** (5%). Purification by flash column chromatography (0 to 15% Et_2O in CH_2Cl_2; R_f 0.27 at 10% Et_2O in CH_2Cl_2) gave the title compound (4 mg, 4%) as a pale yellow gum. **ν_{max}** (film, cm^{-1}) 2957, 2918, 2851, 1628, 1556, 1431, 1298, 1180, 1134, 1090, 1063, 1028, 800, 752; **^{1}H NMR** (500 MHz, $CDCl_3$) δ_H: 1.69 – 1.80 (2H, m,

C(3')H^AH^B and C(4')H^AH^B), 1.80 – 1.94 (2H, m, C(3')H^AH^B and C(4')H^AH^B), 2.91 – 3.02 (1H, m, C(2')H^AH^B), 3.07 – 3.17 (1H, m, C(2')H^AH^B), 3.30 – 3.43 (1H, m, C(5')H^AH^B), 3.43 – 3.53 (1H, m, C(5')H^AH^B), 3.67 (1H, d, *J* 9.4, C(2)*H*), 4.38 – 4.52 (2H, m, C(3''')*H* and N(1''')CH^AH^BPh), 4.73 (1H, ddd, *J* 9.4, 5.9, 1.5, C(2''')*H*), 4.98 (1H, d, *J* 15.3, CH^AH^BPh), 6.23 (1H, dd, *J* 9.2, 1.1, C(4''')*H*), 7.07 – 7.13 (2H, m, C(2'',6'')*H*), 7.17 (2H, d, *J* 6.4, C(2'''',6'''')*H*), 7.20 – 7.24 (3H, m, C(3'',5'')*H* and C(4'')*H*), 7.27 – 7.32 (3H, m, C(3'''',5'''')*H* and C(4'''')*H*), 7.35 (1H, s, C(6''')*H*), 7.43 – 7.57 (3H, m, C(3''''',5''''')*H* and C(4''''')*H*), 7.78 – 7.90 (2H, m, C(2''''',6''''')*H*); **^{13}C{^{1}H} NMR** (126 MHz, $CDCl_3$) δ_C: 24.3 (*C*(3')H_2 or *C*(4')H_2), 26.1 (*C*(3')H_2 or *C*(4')H_2), 46.4 (*C*(2')H_2 or *C*(5')H_2), 46.4 (*C*(2')H_2 or *C*(5')H_2), 53.1 (*C*(2)H), 58.9 (*C*(2''')H), 59.7 (N(1''')CH_2Ph), 110.4 (*C*(5''')), 111.4 (*C*(3''')H), 120.3 (*C*(4''')H), 126.5 (*C*(2''''',6''''')H), 127.3 (*C*(2'''',6'''')H), 127.7 (*C*(4'')H or *C*(4'''')H), 128.0 (*C*(4'')H or *C*(4'''')H), 128.6 (2 ArCH), 129.0 (2 ArCH), 129.1 (2 ArCH), 129.7 (*C*(2'',6'')H), 132.0 (*C*(4''''')H), 133.8 (*C*(1'')), 137.6 (*C*(1'''')), 143.2 (*C*(6''')H), 144.3 (*C*(1''''')), 169.9 (*C*(1)=O); **HRMS** (ESI$^+$) $C_{30}H_{30}N_2NaO_3S$ $[M+Na]^+$ found 521.1867, requires 521.1869 (−0.4 ppm).

(*R*)-2-((*S*)-1'''-Benzyl-3'''-(phenylsulfonyl)-1''',4'''-dihydropyridin-4'''-yl)-1-morpho-lino-2-phenylethan-1-one (**37**)

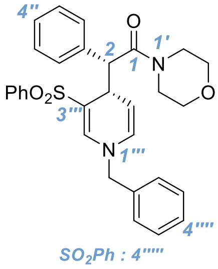

37

Following general procedure D, using 4-nitrophenyl 2-phenylacetate **10** (77 mg, 0.30 mmol, 1.5 equiv), 1-benzyl-3-(phenylsulfonyl)pyridin-1-ium bromide **17** (78.0 mg, 0.20 mmol. 1.0 equiv), (*R*)-BTM (10.1 mg, 0.04 mmol, 20 mol%), DABCO (33.6 mg, 0.3 mmol, 1.5 equiv) and PhMe (1.3 mL, 0.15 M) for 24 h at 0 °C, then morpholine (88 µL, 1.0 mmol, 5.0 equiv) for 24 h at 0 °C, gave crude product (62%, 95:5 dr). Purification by flash column chromatography (6 to 12% Et_2O in CH_2Cl_2; R_f 0.28 at 10% Et_2O in CH_2Cl_2 then 30 to 55% EtOAc in petrol; R_f 0.34 at 60% EtOAc in petrol) gave the title compound (58 mg, 56%, > 95:5 dr) as a pale yellow foam. **$[\alpha]_D^{20}$** −346 (*c* 0.5, $CHCl_3$); **HPLC**: Chiralpak

IB, (80:20 hexane: IPA, flow rate 1.0 mLmin^{-1}, 211 nm, 30 °C) t_R (major): 14.0 min, t_R (minor): 19.2 min, 94:6 er; ν_{max} (film, cm^{-1}) 1633, 1581, 1418, 1275, 1215, 1136, 1113, 1086; **^{1}H NMR** (500 MHz, $CDCl_3$) δ_H: 2.90 – 3.00 (1H, m, C(3')H^AH^B), 3.08 – 3.17 (1H, m, C(2')H^AH^B), 3.22 – 3.31 (1H, m, C(2')H^AH^B), 3.37 – 3.53 (3H, m, C(3')H^AH^B, C(5')H^AH^B and C(6')H^AH^B), 3.62 – 3.69 (1H, m, C(5')H^AH^B), 3.69 – 3.77 (1H, m, C(6')H^AH^B), 3.94 – 4.05 (3H, m, NC*H*2Ph and C(4''')*H*), 4.54 (1H, d, *J* 2.3, C(2)*H*), 5.29 (1H, dd, *J* 8.0, 5.2, C(5''')*H*), 5.53 (1H, dd, *J* 8.0, 1.5, C(6''')*H*), 6.59 (2H, dd, *J* 7.7, 1.7, C(2'''',6'''')*H*), 7.17 (1H, d, *J* 1.1, C(2''')*H*), 7.19 – 7.25 (5H, m, C(3'''',5'''')*H*, C(4'''')*H* and C(2'',6'')*H*), 7.27 – 7.34 (3H, m, C(3'',5'')*H* and C(4'')*H*), 7.46 – 7.54 (2H, m, C(3''''',5''''')*H*), 7.56 – 7.61 (1H, m, C(4''''')*H*), 7.85 – 7.93 (2H, m, C(2''''',6''''')*H*); **^{13}C{^{1}H} NMR** (126 MHz, $CDCl_3$) δ_C: 36.7 (*C*(4''')H), 42.2 (*C*(6')H_2), 46.5 (*C*(2')H_2), 54.3 (*C*(2)H), 57.4 (NCH_2Ph), 66.2 (*C*(3')H_2), 66.8 (*C*(5')H_2), 104.8 (*C*(3''')), 106.8 (*C*(5''')H), 127.0 (*C*(2'''',6'''')H), 127.2 (ArCH), 127.4 (*C*(2''''',6''''')H), 127.9 (ArCH), 128.0 (2 ArCH), 128.9 (2 ArCH), 129.2 (*C*(3''''',5''''')H), 129.3 (*C*(6''')H), 130.6 (*C*(2'',6'')H), 132.7 (*C*(4''''')H), 135.9 (*C*(1'''')), 136.1 (*C*(1'')), 141.3 (*C*(1''''')), 143.3 (*C*(2''')H), 171.8 (*C*(1)=O); **HRMS** (ESI$^+$) $C_{30}H_{30}N_2NaO_4S$ [M+Na]$^+$ found 537.1806, requires 537.1818 (−2.2 ppm).

tert-Butyl-4-((*R*)-2'-((*S*)-1'''-benzyl-3'''-(phenylsulfonyl)-1''',4'''-dihydropyridin-4'''-yl)-2'-phenylacetyl)piperazine-1-carboxylate (**38**)

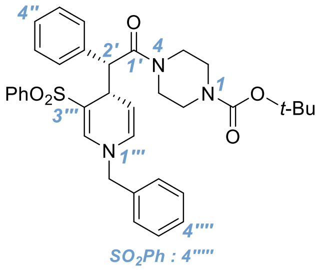

38

Following general procedure D, using 4-nitrophenyl 2-phenylacetate **10** (77 mg, 0.30 mmol, 1.5 equiv), 1-benzyl-3-(phenylsulfonyl)pyridin-1-ium bromide **17** (78.0 mg, 0.20 mmol. 1.0 equiv), (*R*)-BTM (10.1 mg, 0.04 mmol, 20 mol%), DABCO (33.6 mg, 0.3 mmol, 1.5 equiv) and PhMe (1.3 mL, 0.15 M) for 24 h at 0 °C, then 1-Boc-piperazine (186 mg, 1.0 mmol, 5.0 equiv) for 24 h at 0 °C, gave crude product (67%, 90:10 dr). Purification by flash column chromatography (25 to 50% EtOAc in petrol; R_f 0.40 at 50% EtOAc in petrol then 6 to 10% Et_2O in CH_2Cl_2; R_f 0.29 at 10% Et_2O in CH_2Cl_2) gave the title

compound (64 mg, 52%, > 95:5 dr) as a pale yellow foam. $[\alpha]_D^{20}$ −437 (*c* 1.0, $CHCl_3$); **HPLC**: Chiralpak IB, (80:20 hexane: IPA, flow rate 1.0 mLmin^{-1}, 220 nm, 30 °C) t_R (major): 12.1 min, t_R (minor): 17.7 min, 93:7 er; ν_{max} (film, cm^{-1}) 2970, 1691, 1668, 1638, 1581, 1416, 1367, 1284, 1187, 1136, 1086; **^{1}H NMR** (500 MHz, CD_3CN) δ_H: 1.38 (9H, s, C(C*H*$_3$)$_3$), 2.42 – 2.55 (1H, broad-m, C(2)H^AH^B), 2.96 – 3.10 (2H, m, C(6)H^AH^B and C(3)H^AH^B), 3.10 – 3.16 (1H, m, C(3)H^AH^B), 3.16 – 3.22 (1H, m, C(2)H^AH^B), 3.24 – 3.34 (1H, m, C(5)H^AH^B), 3.37 – 3.46 (1H, m, C(6)H^AH^B), 3.58 – 3.69 (1H, m, C(5)H^AH^B), 3.88 (1H, dd, *J* 5.1, 2.3, C(4''')*H*), 4.09 (1H, d, *J* 15.7, NCH^AH^BPh), 4.14 (1H, d, *J* 15.7, NCH^AH^BPh), 4.31 (1H, d, *J* 2.3, C(2')*H*), 5.12 (1H, dd, *J* 8.0, 5.1, C(5''')*H*), 5.61 (1H, dd, *J* 8.0, 1.3, C(6''')*H*), 6.67 (1H, dd, *J* 6.6, 2.9, C(2'''',6'''')*H*), 7.10 (2H, dd, *J* 8.1, 1.2, C(2'',6'')*H*), 7.20 (1H, d, *J* 1.1, C(2''')*H*), 7.22 – 7.30 (5H, m, C(3'',5'')*H*, C(3'''',5'''')*H* and C(4'''')*H*), 7.30 – 7.35 (1H, m, C(4'')*H*), 7.61 (2H, tt, *J* 6.8, 1.6, C(3''''',5''''')*H*), 7.63 – 7.69 (1H, m, C(4''''')*H*), 7.85 – 7.94 (2H, m, C(2''''',6''''')*H*); **^{13}C{^{1}H} NMR** (126 MHz, CD_3CN) δ_C: 28.4 (C(CH_3)$_3$), 37.4 (*C*(4''')H), 42.2 (*C*(5)H_2), 43.3 (*C*(2)H_2 or *C*(5)H_2), 44.4 (*C*(2)H_2 or *C*(5)H_2), 46.2 (*C*(3)H_2), 54.9 (*C*(2')H), 57.5 (NCH_2Ph), 80.2 (*C*(CH_3)$_3$), 105.8 (*C*(3''')), 106.7 (*C*(5''')H), 127.9 (*C*(2'''',6'''')H), 128.0 (*C*(4'')H), 128.0 (*C*(2''''',6''''')H), 128.4 (*C*(4'''')H), 128.8 (2 ArCH), 129.6 (2 ArCH), 130.3 (*C*(3''''',5''''')H), 130.4 (*C*(6''')H), 131.2 (*C*(2'',6'')H), 133.7 (*C*(4''''')H), 137.1 (*C*(1'')), 137.8 (*C*(1'''')), 142.9 (*C*(1''''')), 144.1 (*C*(2''')H), 155.2 (*C*=$O_{carbamate}$), 171.8 (*C*(1')=O); **HRMS** (ESI$^+$) $C_{35}H_{39}N_3NaO_5S$ [M+Na]$^+$ found 636.2491, requires 636.2503 (−1.9 ppm).

6. Control experiments

6.1. Control reaction of catalyst and pyridinium salt

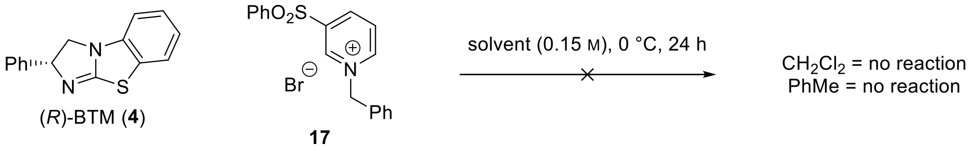

6.2. Reaction of DABCO and pyridinium salt in PhMe

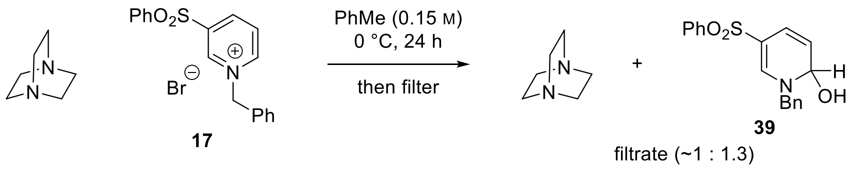

1-Benzyl-3-(phenylsulfonyl)pyridin-1-ium bromide **17** (78 mg, 0.2 mmol, 1.0 equiv) and DABCO (22.4 mg, 0.2 mmol, 1.5 equiv) were weighed into a 20 mL test tube. The test tube was sealed, purged, lowered into a cryostat bath at 0 °C. PhMe (1.3 mL, 0.15 M) was added and the reaction was stirred at 0 °C for 24 h. Upon completion, the reaction mixture was filtered and washed with PhMe (10 mL). The filtrate was concentrated under reduced pressure to give a mixture of DABCO : 1-benzyl-5-(phenylsulfonyl)-1,2-dihydropyridin-2-ol **39** (~1:1.3) as an orange solid (5 mg).

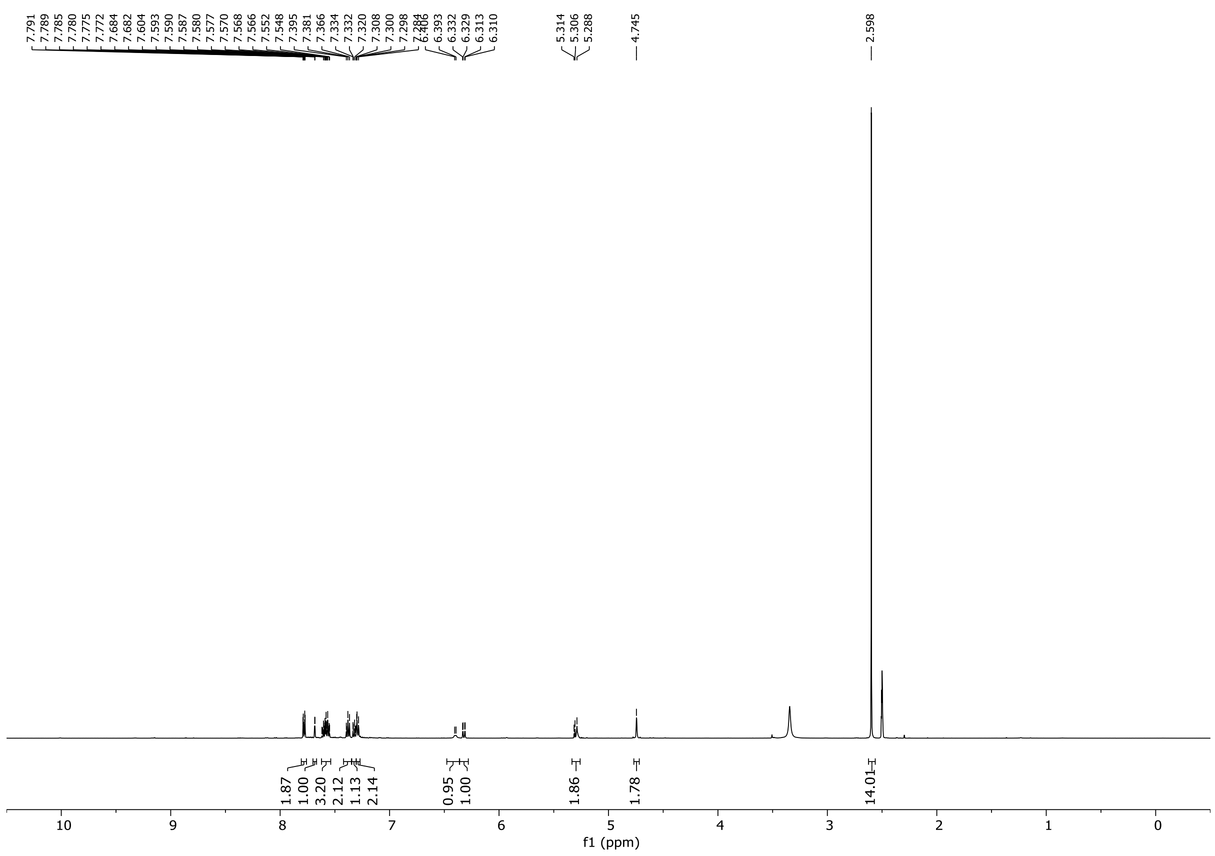

(DABCO = 2.60 ppm)

1-Benzyl-5-(phenylsulfonyl)-1,2-dihydropyridin-2-ol (**39**) characterised as mixture with DABCO (~1.3:1)

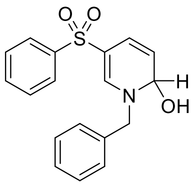

39

ν$_{max}$ (film, cm^{-1}) 3049, 2941, 2872, 2620, 1628, 1572, 1447, 1354, 1298, 1178, 1167, 1092, 1059, 1011, 997, 851, 777; **^{1}H NMR** (500 MHz, DMSO) δ$_H$: 4.74 (2H, s, N(1)C*H*$_2$), 5.26 – 5.34 (2H, m, C(2)*H* and C(3)*H*), 6.32 (1H, dd, *J* 9.5, 1.8, C(4)*H*), 6.40 (1H, broad d, *J* 6.3, O*H*), 7.24 – 7.31 (2H, m, C(2',6')*H*), 7.31 – 7.34 (1H, m, C(4')*H*), 7.38 (2H, app t, *J* 7.2, C(3',5')*H*), 7.53 – 7.64 (3H, m, C(3'',5'')*H* and C(4'')*H*), 7.65 – 7.70 (1H, m, C(6)*H*), 7.74 – 7.81 (2H, m, C(2'',6'')*H*); **^{13}C{^{1}H} NMR** (126 MHz, DMSO) δ$_C$: 55.0 (N(1)CH$_2$), 75.3 (*C*(2)OH), 105.7 (*C*(5)), 113.5 (*C*(3)H), 119.8 (*C*(4)H), 126.0 (*C*(2'',6'')H), 127.9 (*C*(4')H), 128.0 (*C*(2',6')H), 128.7

(C(3',5')H), 129.3 (C(3'',5'')H), 132.2 (C(4'')H), 136.6 (C(1')), 142.5 (C(6)H), 143.9 (C(1''));
HRMS (ESI+) $C_{18}H_{16}NO_2S$ [M]+ found 327.0908, requires 327.0924 (−4.9 ppm).

The presence of the alcohol peak (6.40 ppm) was confirmed by carrying out a D_2O shake, upon which it was not observable.

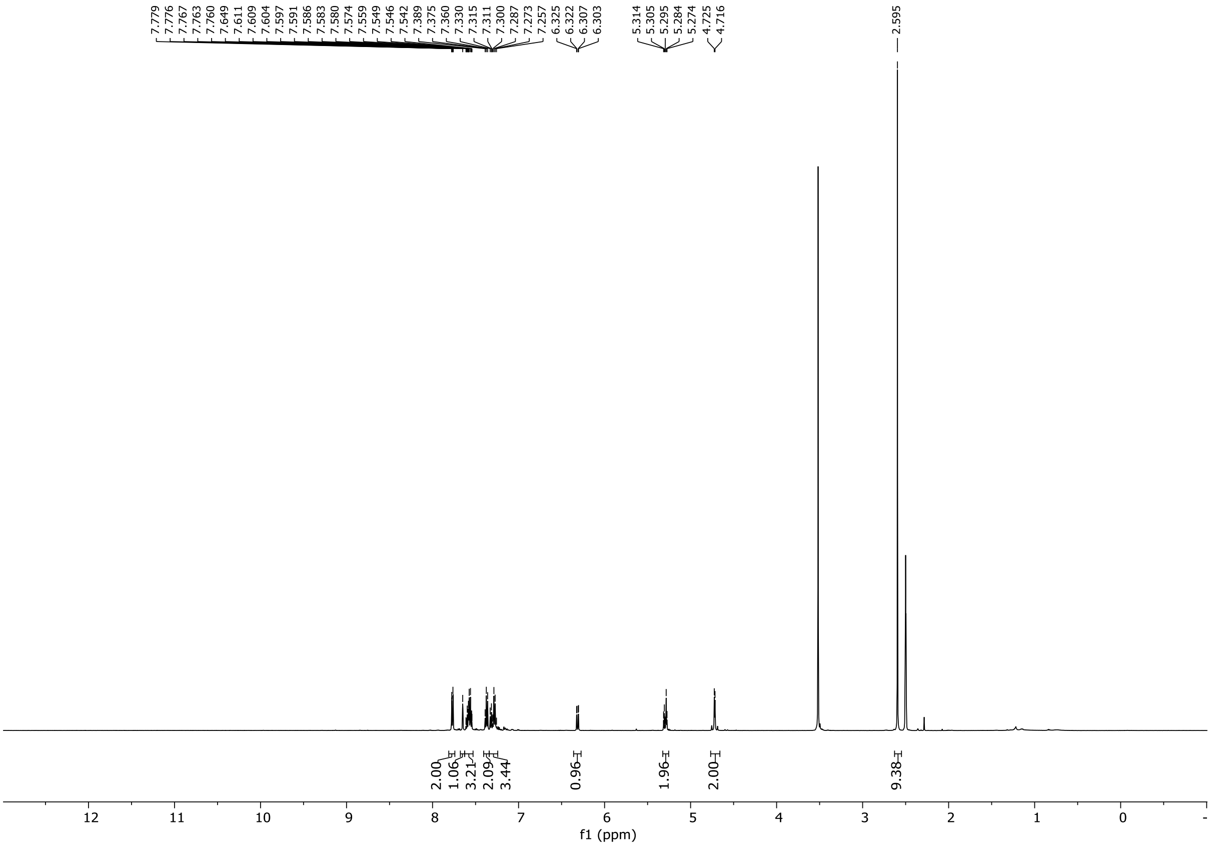

6.3. Subjecting product 39 to the catalysis conditions

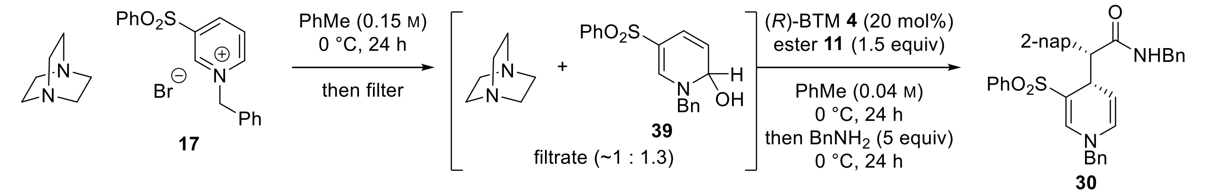

1-Benzyl-3-(phenylsulfonyl)pyridin-1-ium bromide **17** (78 mg, 0.2 mmol, 1.0 equiv) and DABCO (22.4 mg, 0.2 mmol, 1.5 equiv) were weighed into a 20 mL test tube. The test tube was sealed, purged, lowered into a cryostat bath at 0 °C. PhMe (1.3 mL, 0.15 M) was added and the reaction was stirred at 0 °C for 24 h. Upon completion, the reaction mixture was filtered and washed with PhMe (10 mL). The previous carried out as a duplicate. The filtrates of the two reactions were concentrated almost to dryness, combined in a flame

dried 10 mL test tube and dissolved in anhydrous toluene (0.6 mL, 0.04 M). Based on the amount of hydroxydihydropyridine determined in experiment A, 4-Nitrophenyl 2-(naphthalen-2-yl)acetate (**11**) (12 mg, 0.04 mmol, 1.5 equiv) and (*R*)-BTM (1.3 mg, 5 μmol, 20 mol%) were added, the tube sealed, purged and lowered into a cryostat bath stirred at 0 °C for 24 h. Benzylamine (13 μL, 0.12 mmol, 5.0 equiv) and the reaction was stirred at 0 °C for a further 24 h. The reaction mixture was quenched with 1 M NaOH and extracted with CH_2Cl_2 (3 ×). The organic layer was washed successively with 1 M NaOH (2 ×) and brine (1 ×), dried over $MgSO_4$, filtered, and concentrated under reduced pressure. The crude product was purified by column chromatography (4% Et_2O in CH_2Cl_2) to give (*R*)-*N*-Benzyl-2-((*R*)-1-benzyl-3-(phenylsulfonyl)-1,4-dihydropyridin-4-yl)-2-(naphthalen-2-yl)acetamide (**30**) as pale yellow glass (6 mg, 40% yield from filtrate, 6% yield from pyridinium salt).

6.4. Reaction of DABCO and pyridinium salt in CH_2Cl_2

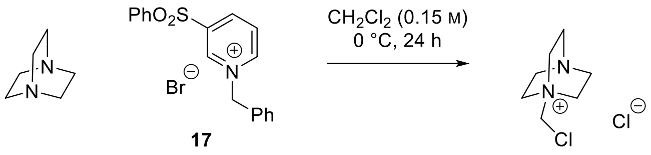

1-Benzyl-3-(phenylsulfonyl)pyridin-1-ium bromide **17** (78 mg, 0.2 mmol, 1.0 equiv) and DABCO (22.4 mg, 0.2 mmol, 1.5 equiv) were weighed into a 20 mL test tube. The test tube was sealed, purged, lowered into a cryostat bath at 0 °C. CH_2Cl_2 (1.3 mL, 0.15 M) was added and the reaction was stirred at 0 °C for 24 h. Upon completion, the reaction mixture was concentrated under reduced pressure. Quaternised DABCO with CH2Cl2 was observed with starting materials. Attempts to purify the mixture were unsuccessful.

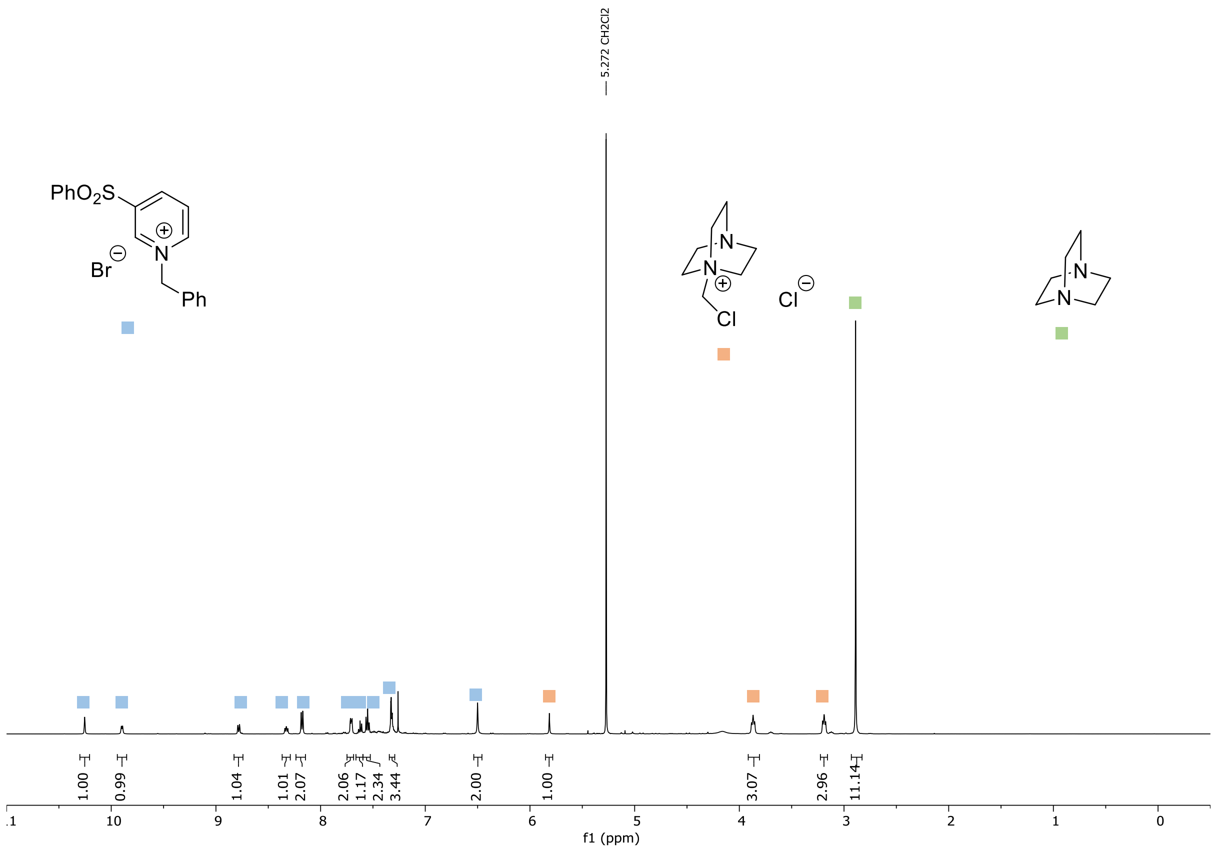

5.272 CH2Cl2
PhO2S
Br
N
Ph
N
N
Cl
Cl
N
N
1.00
0.99
1.04
1.01
2.07
2.06
1.17
2.34
3.44
2.00
1.00
3.07
2.96
11.14
10
9
8
7
6
5
4
3
2
1
0
f1 (ppm)

7. Additional optimisation studies

Catalyst screen in toluene:

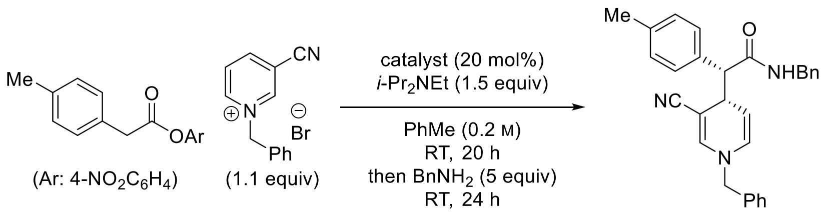

Entry	Catalyst	Yield[a] (%)	dr[b]	er[c]
1	(*R*)-BTM	74	85:15	65:35
2	(*S*)-TM·HCl	65	80:20	48:52
3	(2*S*,3*R*)-HyperBTM	78	80:20	52:48

[a] Combined yield of diastereoisomers. Determined by ^{1}H NMR analysis of the crude reaction mixture using 1,3,5-trimethoxybenzene internal standard. [b] Determined by ^{1}H NMR analysis of the crude reaction mixture. [c] Determined by chiral HPLC analysis.

Background reaction observed for trifluoromethylated ester:

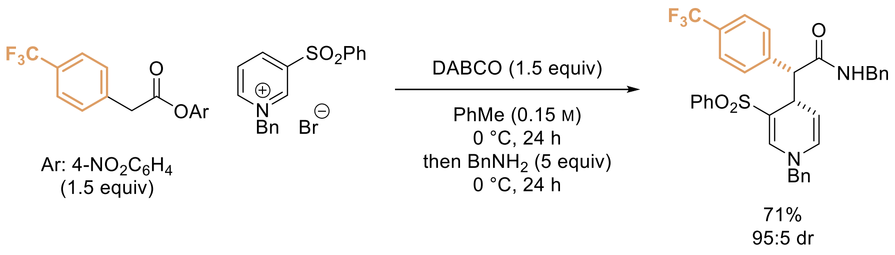

8. X-Ray structure

31

**31** (±)	
**empirical formula**	$C_{35}H_{34}Cl_2N_2O_3S$
**formula weight**	633.63
**crystal description**	colourless, prism
**space group**	C2/c (#15)
a **[Å]**	27.4341(8)
b **[Å]**	14.0679(4)
c **[Å]**	16.1319(5)
**vol [Å]^3**	6191.0(3)
Z	8
**reflns collected**	35889
**Ind. reflns (*R*$_{int}$)**	6726 (0.0627)
**R_1 [I > 2σ(I)]**	0.0372
**wR_2 (all data)**	0.1065

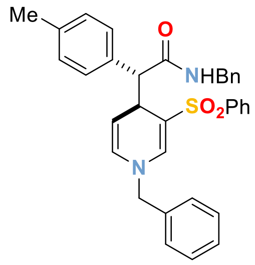

31

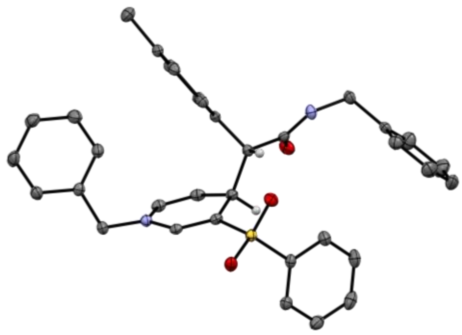

9. References

[1] L. C. Morrill, J. Douglas, T. Lebl, A. M. Z. Slawin, D. J. Fox, A. D. Smith, *Chem. Sci.* **2013**, *4*, 4146–4155.

[2] D. S. B. Daniels, S. R. Smith, T. Lebl, P. Shapland, A. D. Smith, *Synthesis* **2015**, *47*, 34–41.

[3] L. Hao, Y. Du, H. Lv, X. Chen, H. Jiang, Y. Shao, Y. R. Chi, *Org. Lett.* **2012**, *14*, 2154–2157.

[4] J. N. Arokianathar, A. B. Frost, A. M. Z. Slawin, D. Stead, A. D. Smith, *ACS Catal.* **2018**, *8*, 1153–1160.

[5] D. Dev, N. B. Palakurthy, K. Thalluri, J. Chandra, B. Mandal, *J. Org. Chem.* **2014**, *79*, 5420–5431.

[6] C. McLaughlin, A. M. Z. Slawin, A. D. Smith, *Angew. Chem. Int. Ed.* **2019**, *58*, 15111–15119.

[7] F. S. Roodsari, D. Wu, G. S. Pum, J. Hajdu, *J. Org. Chem.* **1999**, *64*, 7727–7737.

[8] G. S. Cockerill, P. C. Levett, D. A. Whiting, *J. Chem. Soc., Perkin Trans. 1* **1995**, 1103–1113.

[9] C. E. Paul, S. Gargiulo, D. J. Opperman, I. Lavandera, V. Gotor-Fernández, V. Gotor, A. Taglieber, I. W. C. E. Arends, F. Hollmann, *Org. Lett.* **2013**, *15*, 180–183.

[10] C. Baumert, M. Günthel, S. Krawczyk, M. Hemmer, T. Wersig, A. Langner, J. Molnár, H. Lage, A. Hilgeroth, *Bioorg. Med. Chem.* **2013**, *21*, 166–177.

[11] N.-W. Liu, K. Hofman, A. Herbert, G. Manolikakes, *Org. Lett.* **2018**, *20*, 760–763.

[12] D. Passarella, R. Favia, A. Giardini, G. Lesma, M. Martinelli, A. Silvani, B. Danieli, S. M. N. Efange, D. C. Mash, *Bioorg. Med. Chem.* **2003**, *11*, 1007–1014.

[13] S. Kulchat, J.-M. Lehn, *Chem. Asian J.* **2015**, *10*, 2484–2496.

[14] M. J. van Haren, J. S. Toraño, D. Sartini, M. Emanuelli, R. B. Parsons, N. I. Martin, *Biochemistry* **2016**, *55*.

10. Appendix I : ^{1}H and $^{13}C\{^{1}H\}$ NMR spectra of novel compounds

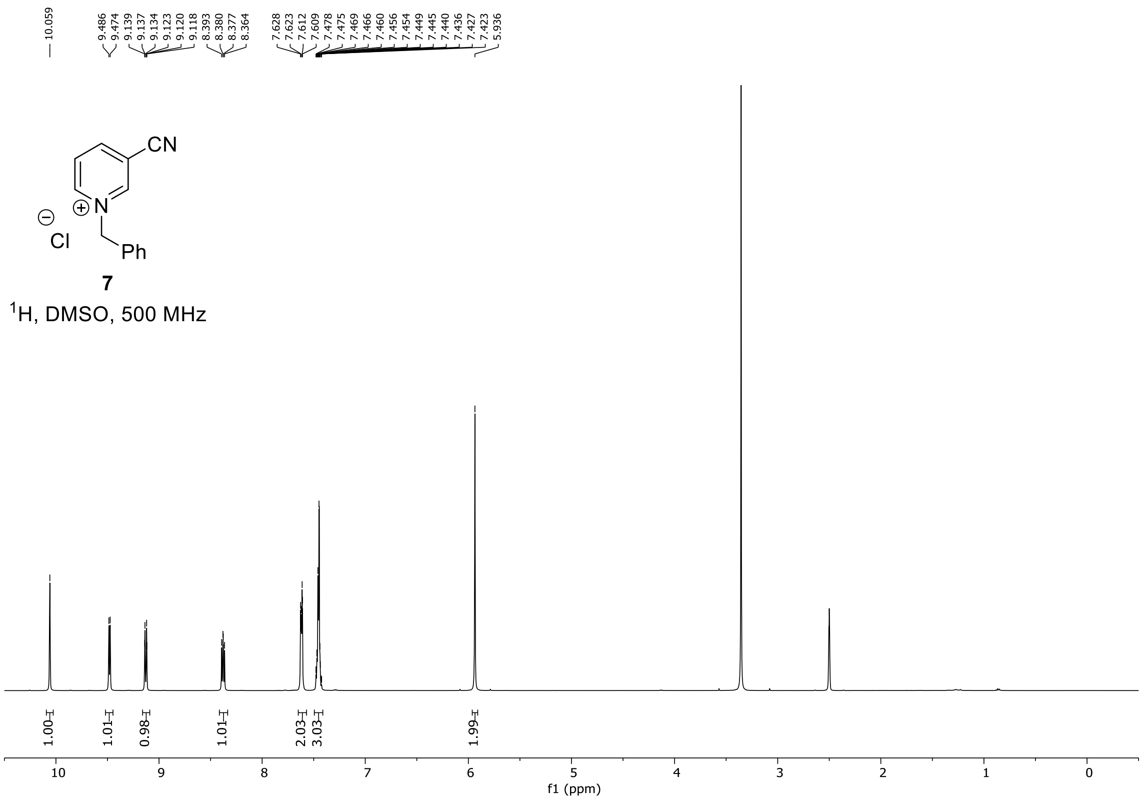
10.059
9.486
9.474
9.139
9.137
9.134
9.123
9.120
9.118
8.393
8.380
8.377
8.364
7.628
7.623
7.612
7.609
7.478
7.475
7.469
7.466
7.460
7.456
7.454
7.449
7.445
7.440
7.436
7.427
7.423
5.936
CN
N
Cl
Ph
7
1H, DMSO, 500 MHz
1.00
1.01
0.98
1.01
2.03
3.03
1.99
10
9
8
7
6
5
4
3
2
1
0
f1 (ppm)

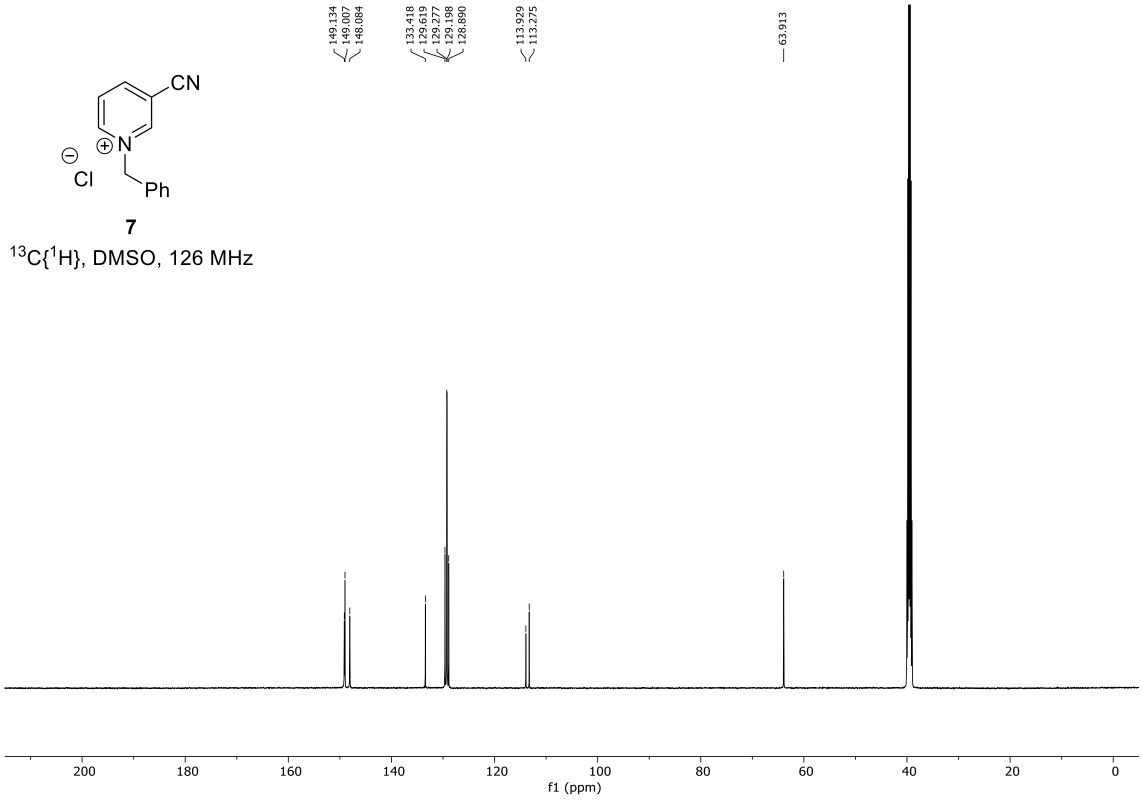
149.134
149.007
148.084
133.418
129.619
129.277
129.198
128.890
113.929
113.275
63.913
CN
N
Cl
Ph
7
13C{1H}, DMSO, 126 MHz
200
180
160
140
120
100
80
60
40
20
0
f1 (ppm)

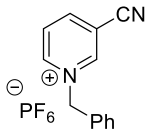

8

^1H, DMSO, 500 MHz

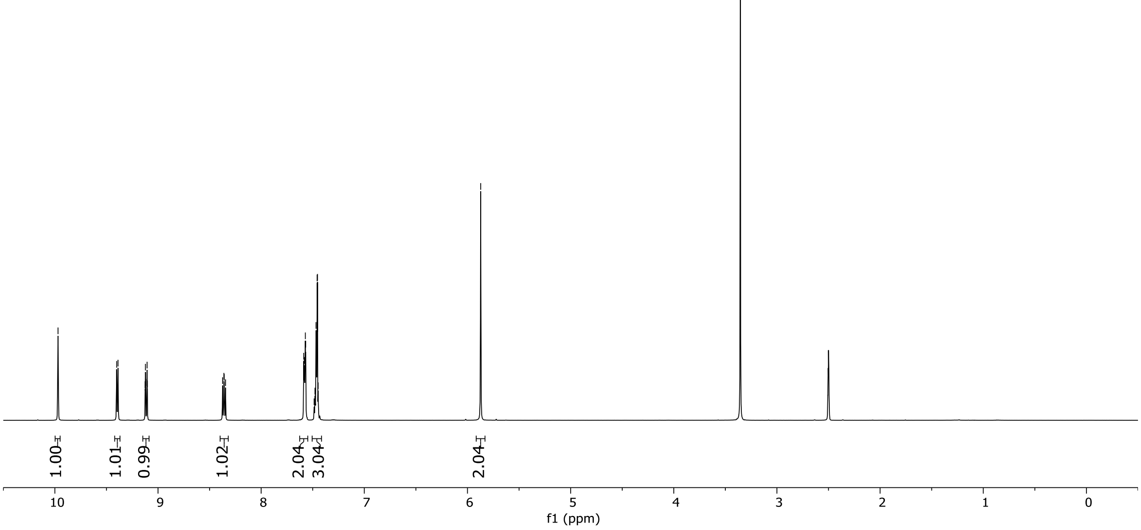

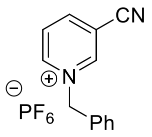

8

$^{13}C\{^1H\}$, DMSO, 126 MHz

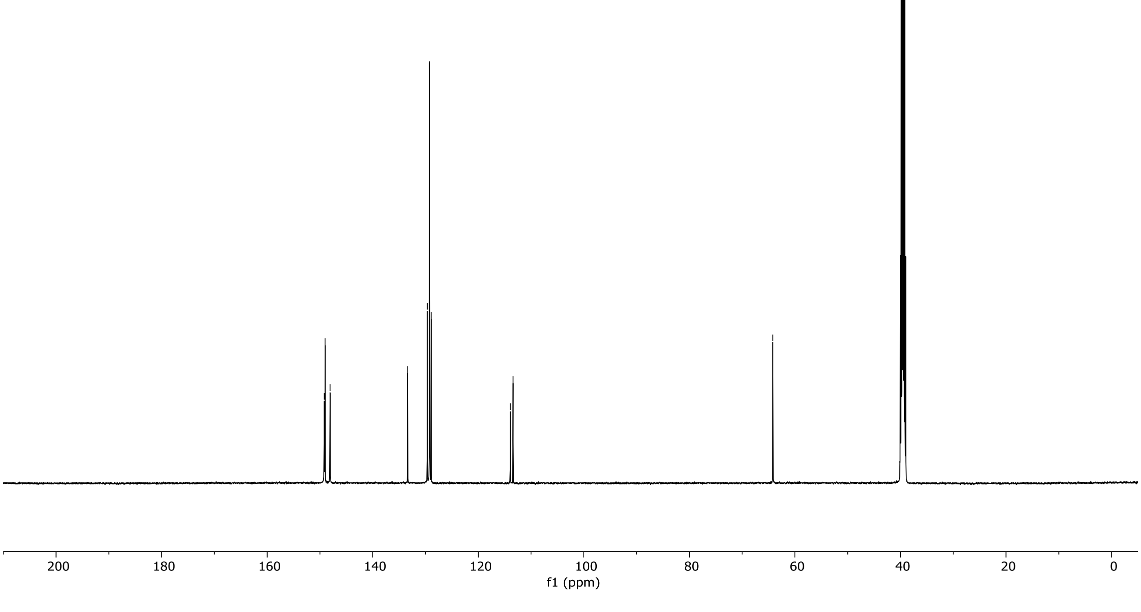

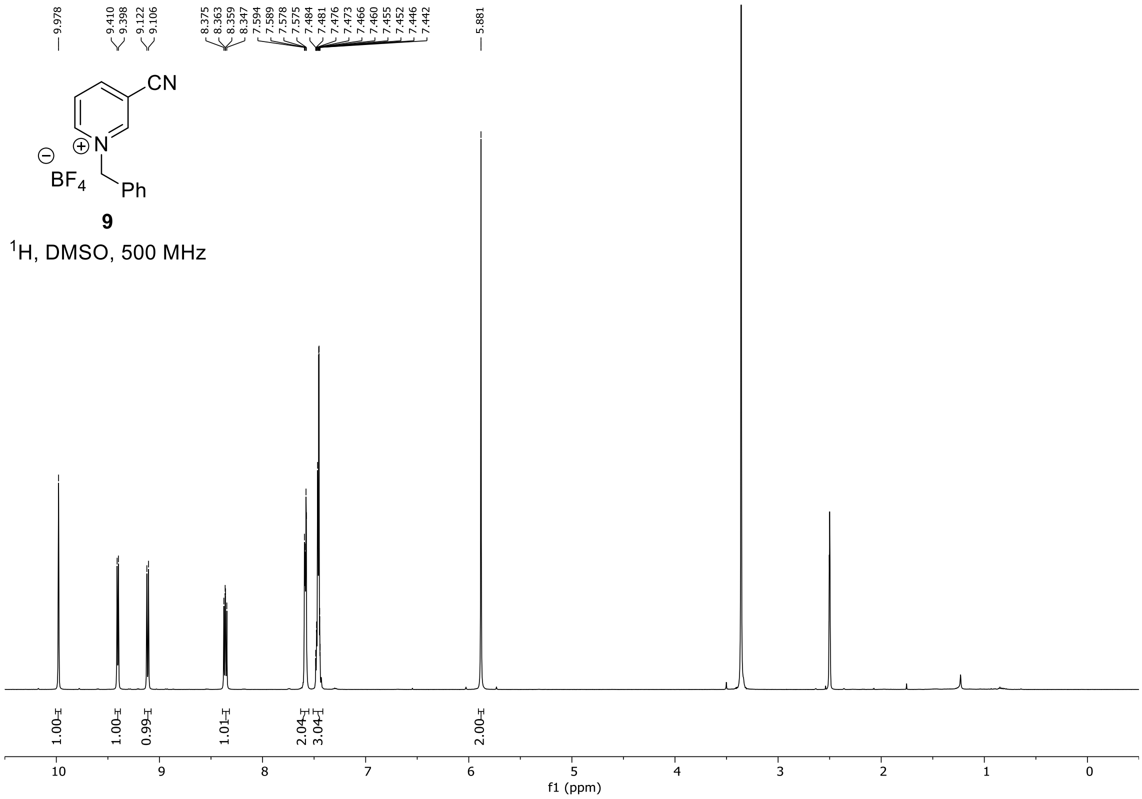
9.978
9.410
9.398
9.122
9.106
8.375
8.363
8.359
8.347
7.594
7.589
7.578
7.575
7.484
7.481
7.476
7.473
7.466
7.460
7.455
7.452
7.446
7.442
5.881
CN
BF4
Ph
9
1H, DMSO, 500 MHz
1.00
1.00
0.99
1.01
2.04
3.04
2.00
f1 (ppm)

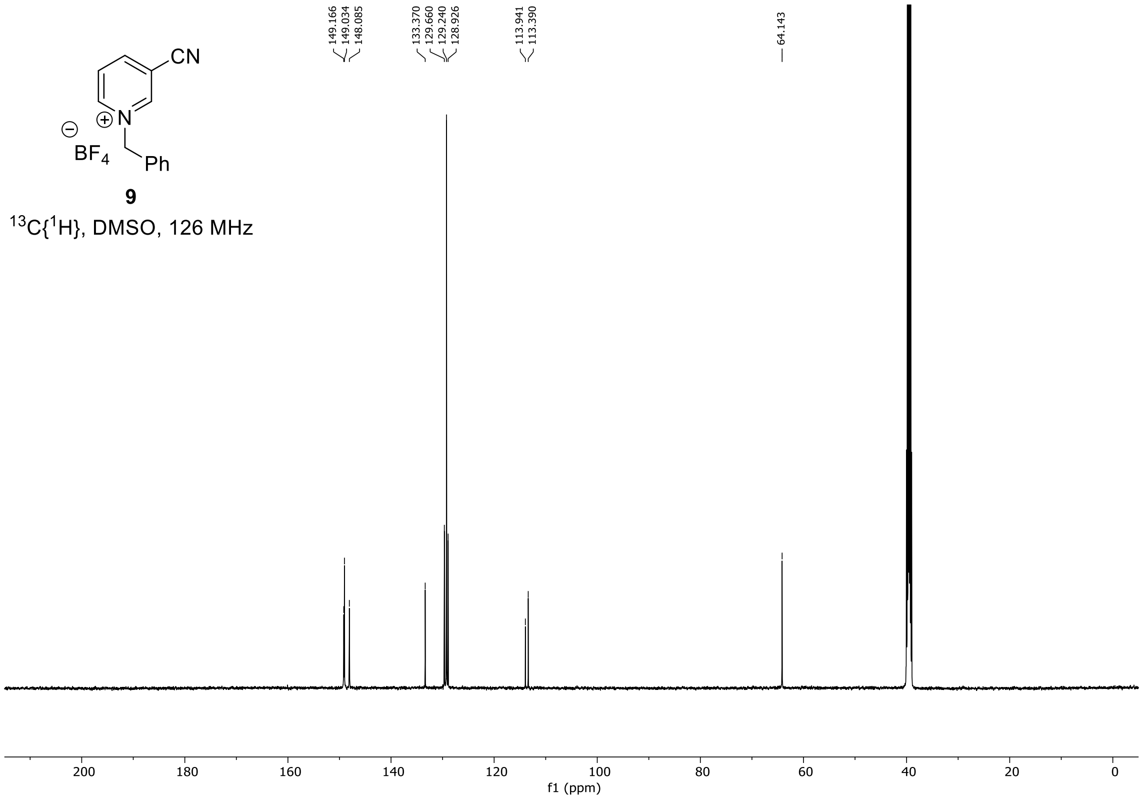
149.166
149.034
148.085
133.370
129.660
129.240
128.926
113.941
113.390
64.143
CN
BF4
Ph
9
13C{1H}, DMSO, 126 MHz
f1 (ppm)

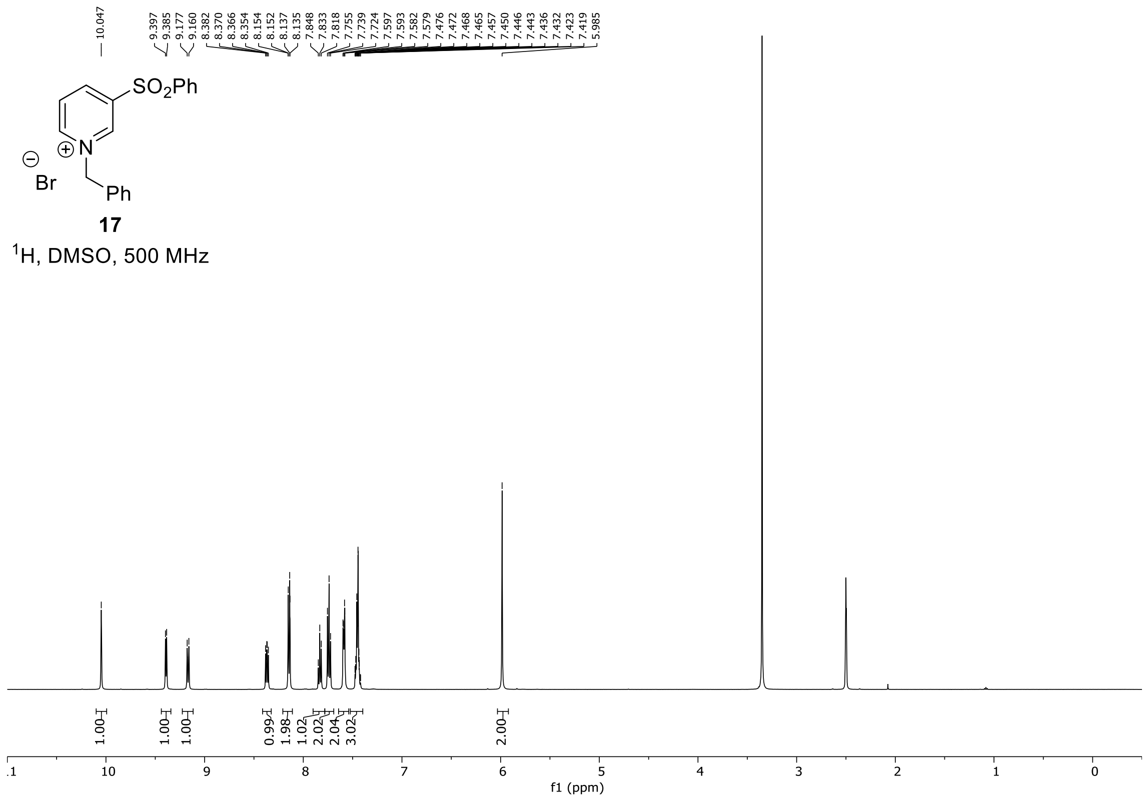

17

^{1}H, DMSO, 500 MHz

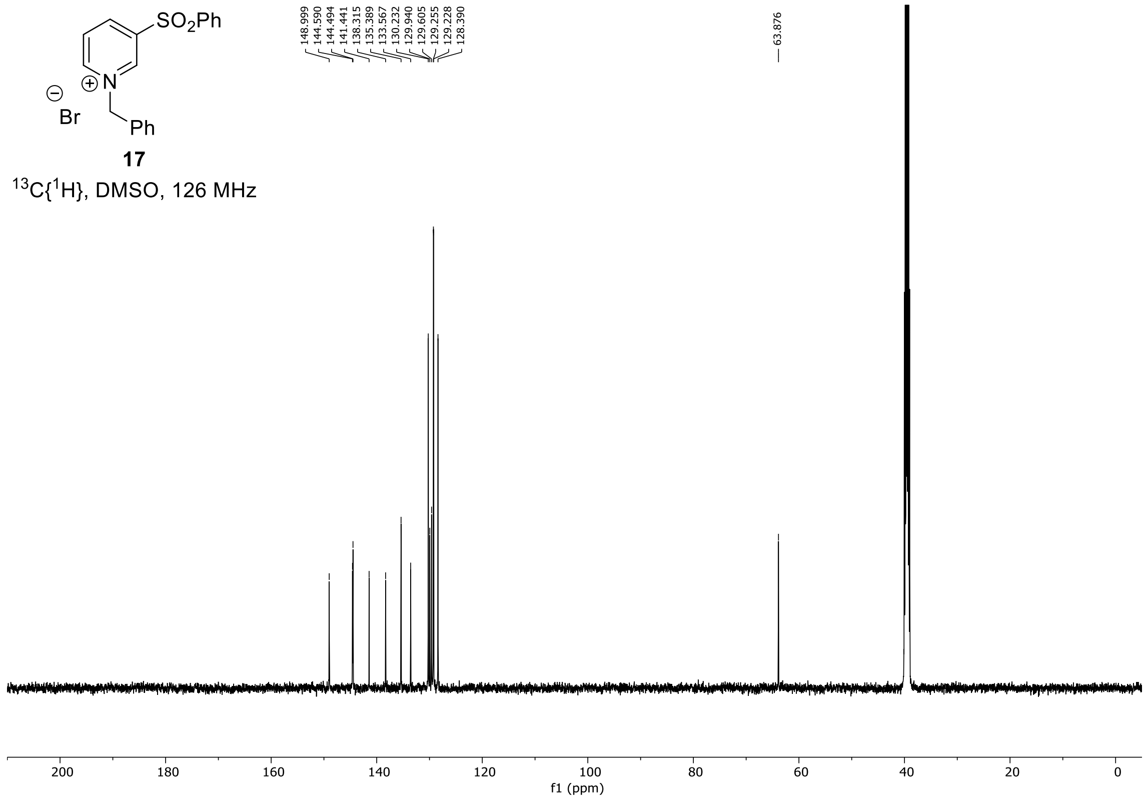

17

^{13}C{^{1}H}, DMSO, 126 MHz

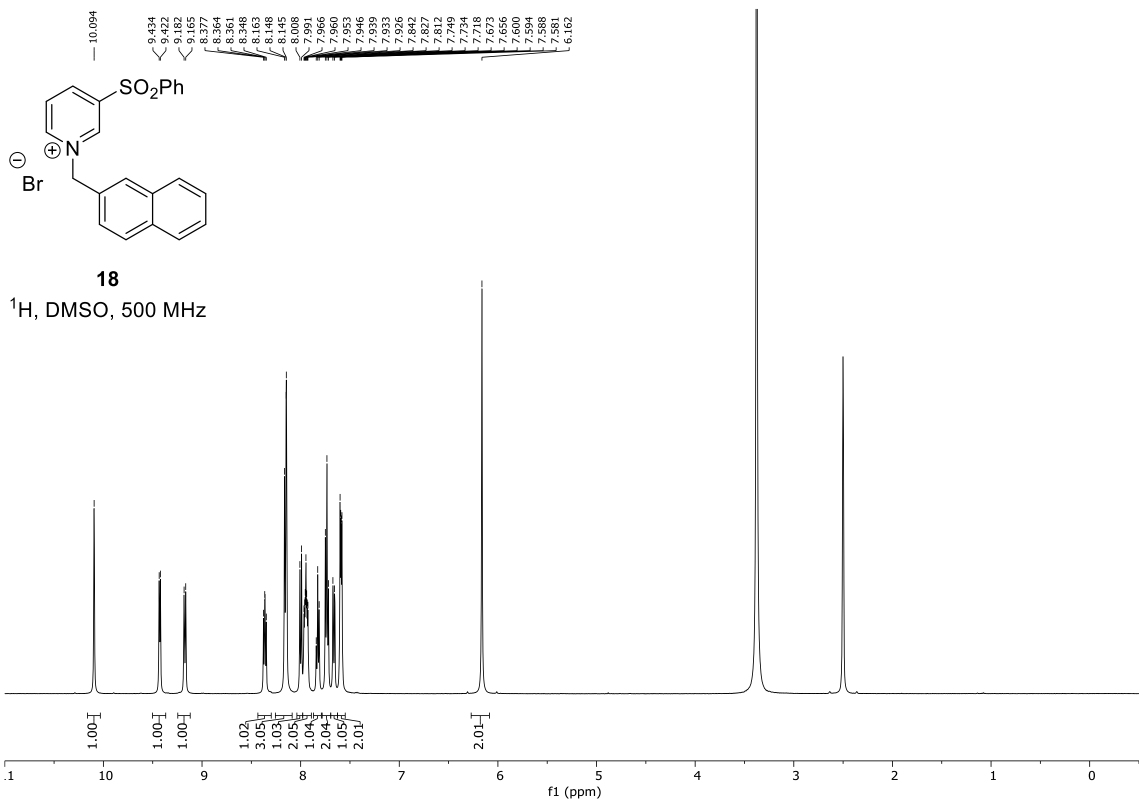

18
^{1}H, DMSO, 500 MHz
f1 (ppm)

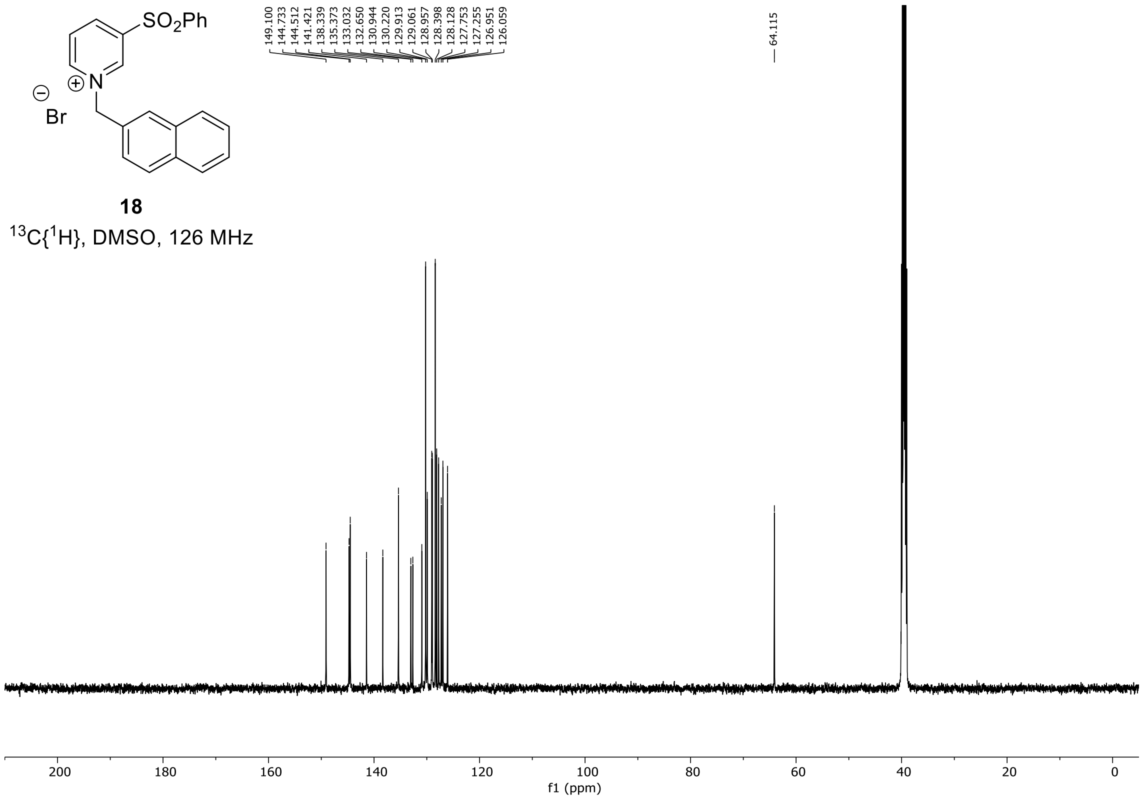

18
^{13}C{^{1}H}, DMSO, 126 MHz
f1 (ppm)

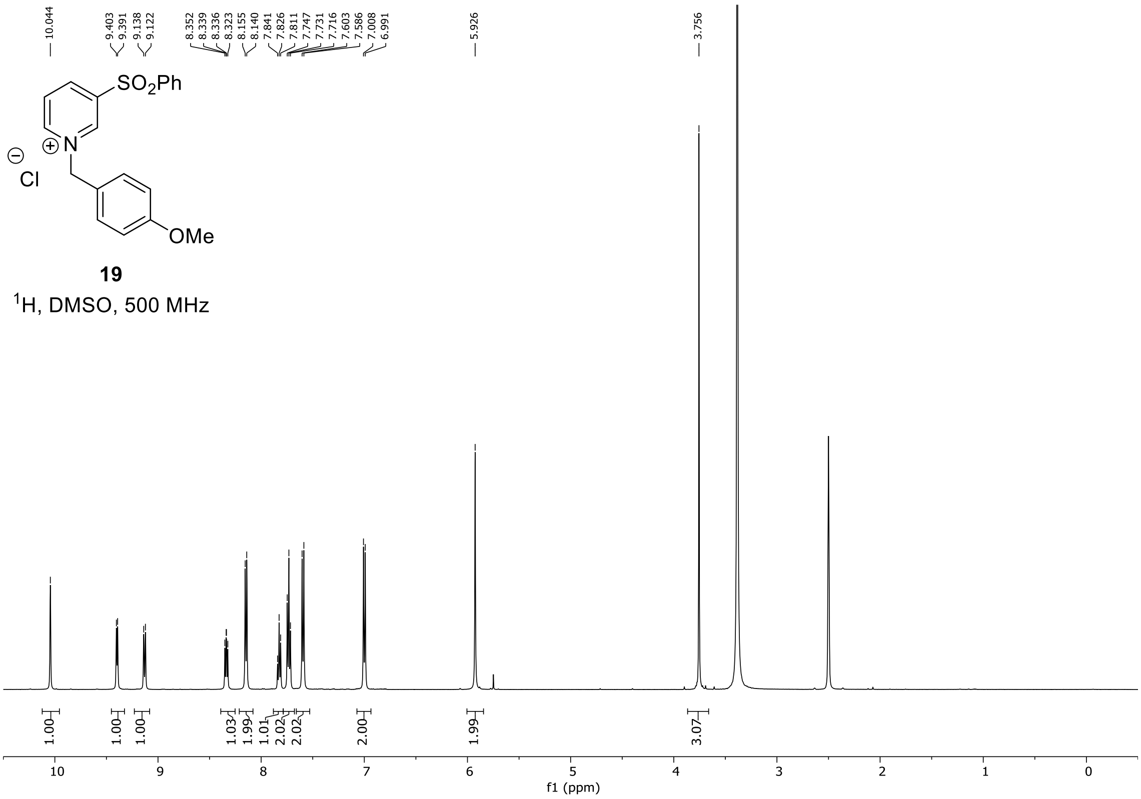

19

^{1}H, DMSO, 500 MHz

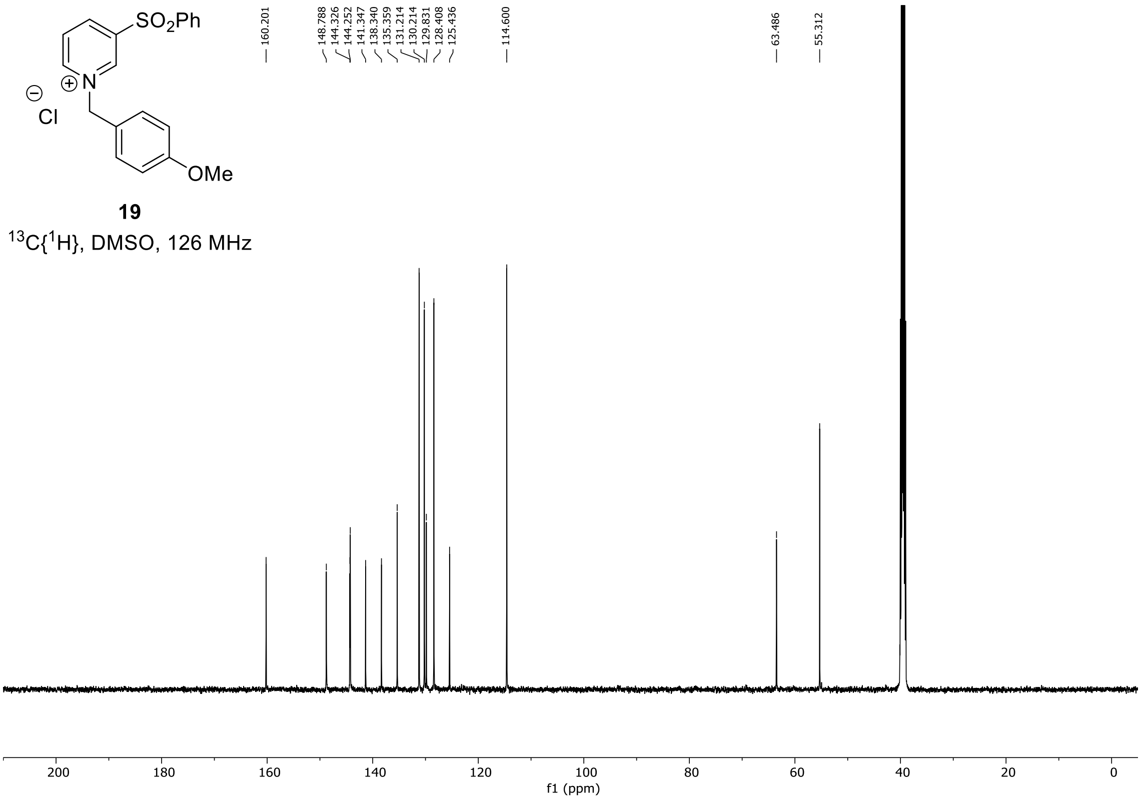

19

$^{13}C\{^{1}H\}$, DMSO, 126 MHz

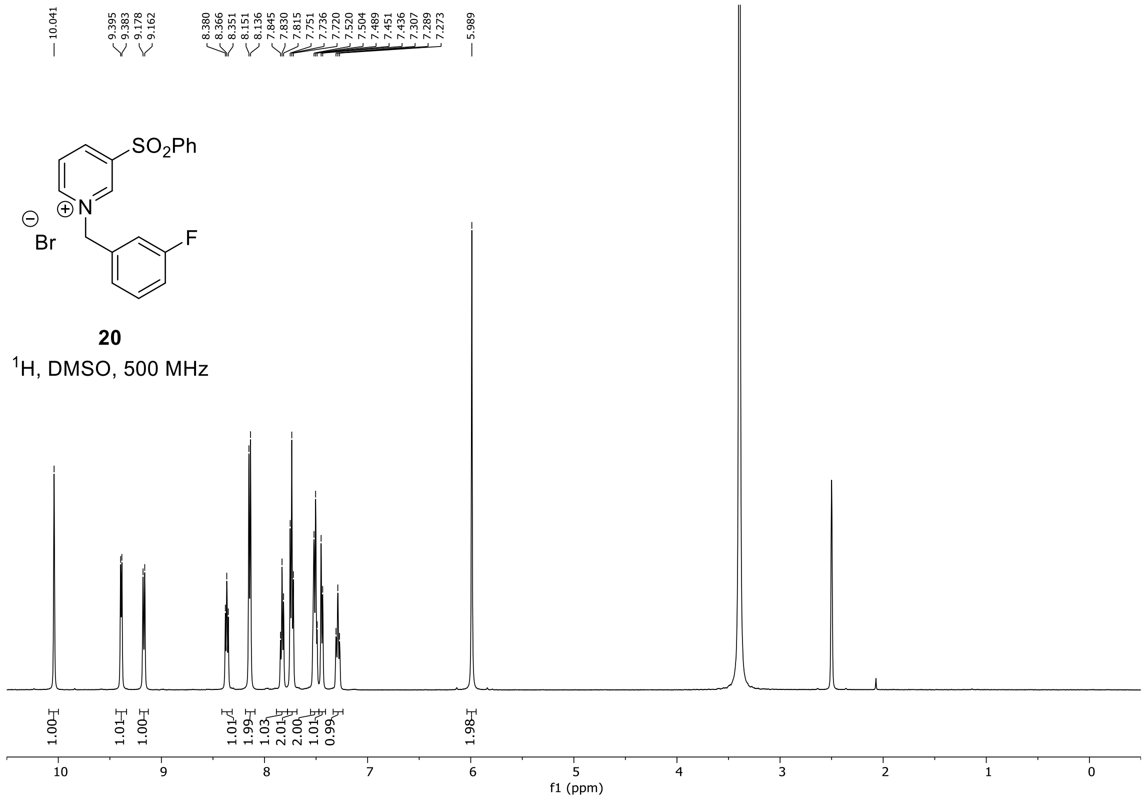

20

^{1}H, DMSO, 500 MHz

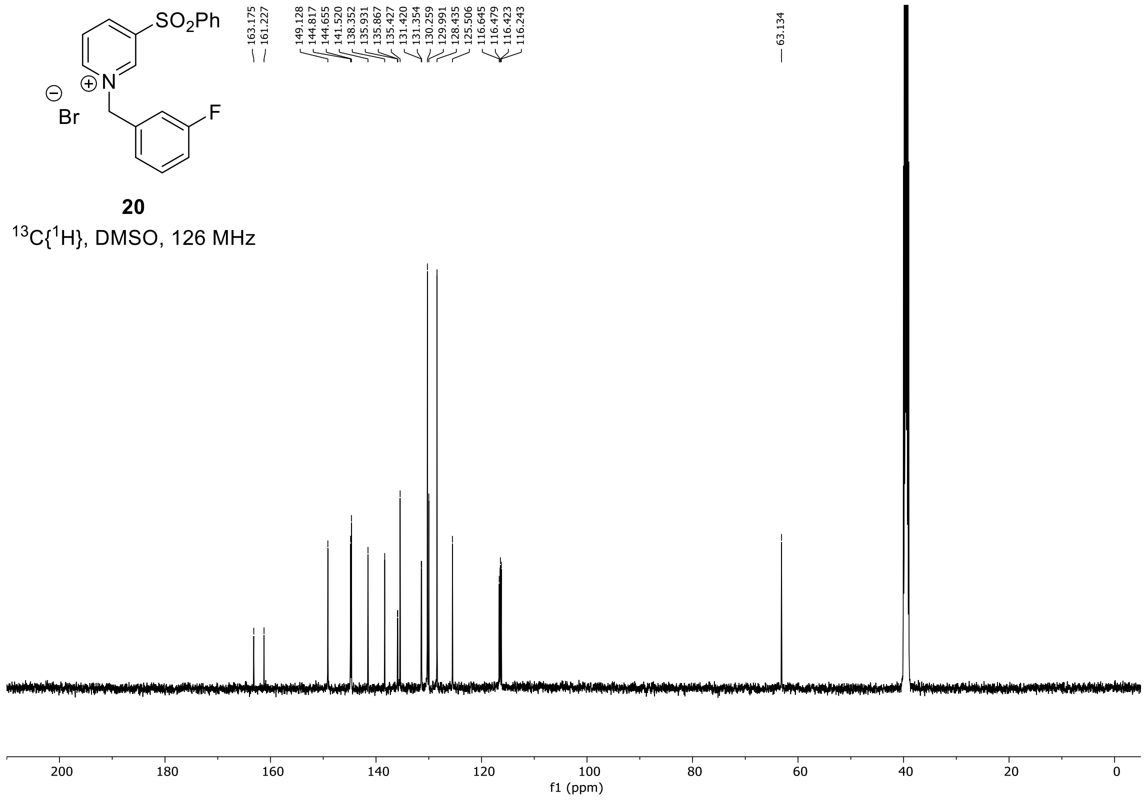

20

$^{13}C\{^{1}H\}$, DMSO, 126 MHz

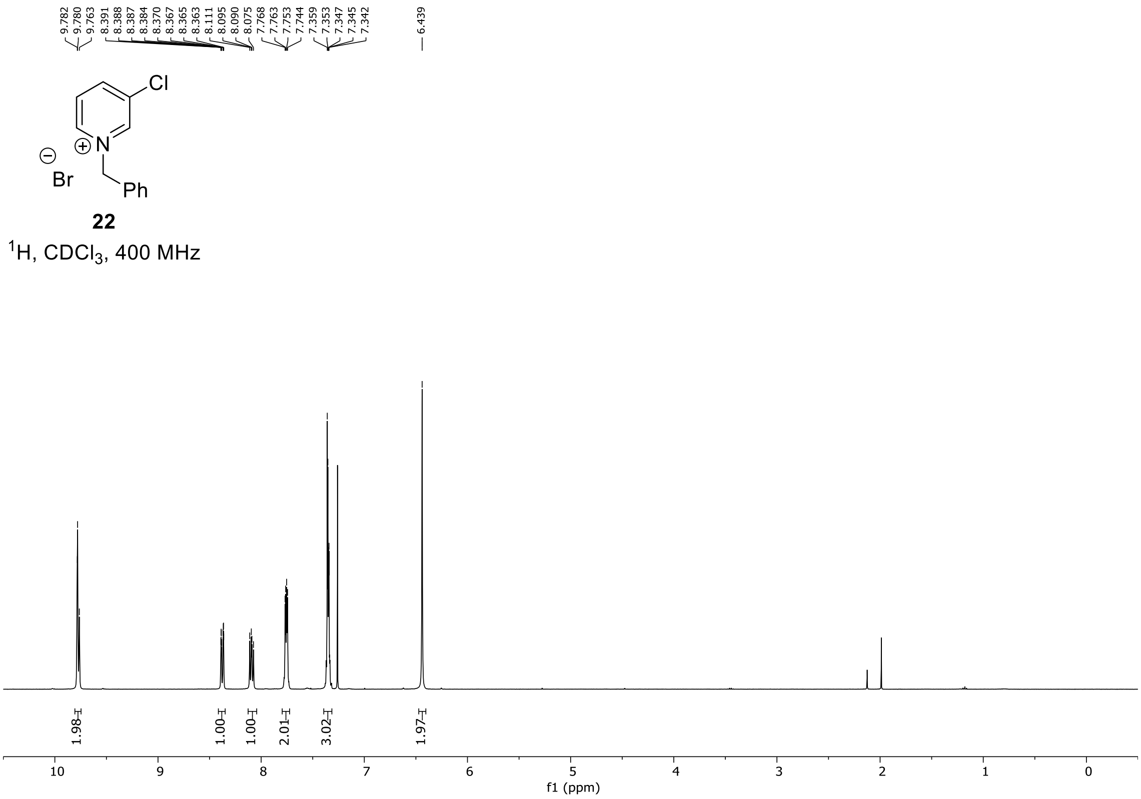

22

^{1}H, $CDCl_3$, 400 MHz

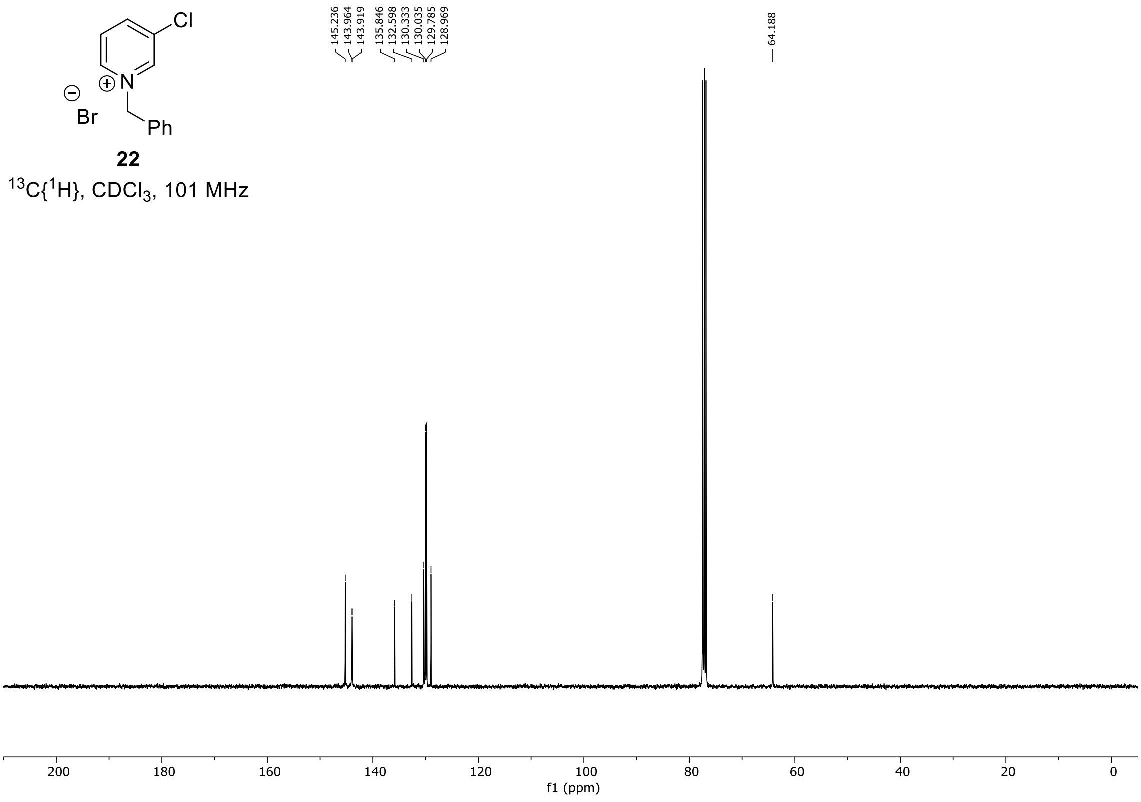

22

$^{13}C\{^{1}H\}$, $CDCl_3$, 101 MHz

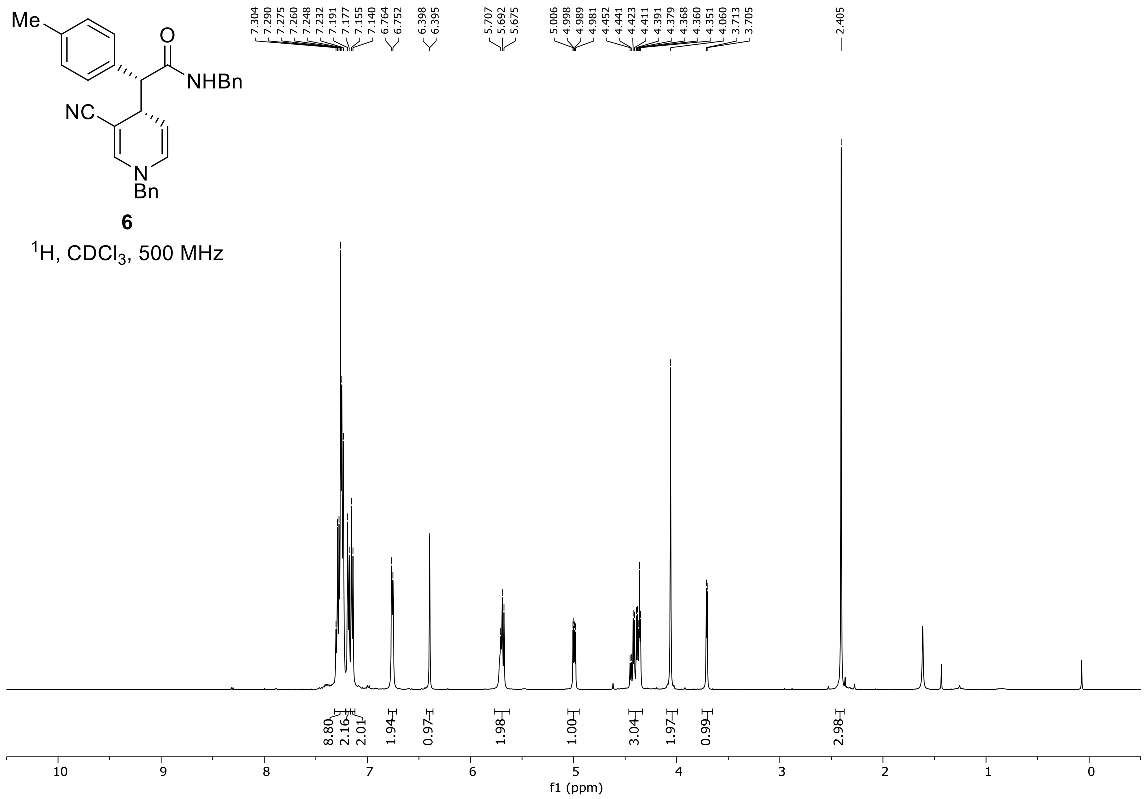

Me
O
NHBn
NC
N
Bn
6
^{1}H, CDCl$_3$, 500 MHz
7.304 7.290 7.275 7.260 7.248 7.232 7.191 7.177 7.155 7.140 6.764 6.752 6.398 6.395 5.707 5.692 5.675 5.006 4.998 4.989 4.981 4.452 4.441 4.423 4.411 4.391 4.379 4.368 4.360 4.351 4.060 3.713 3.705 2.405
8.80 2.16 2.01 1.94 0.97 1.98 1.00 3.04 1.97 0.99 2.98
f1 (ppm)

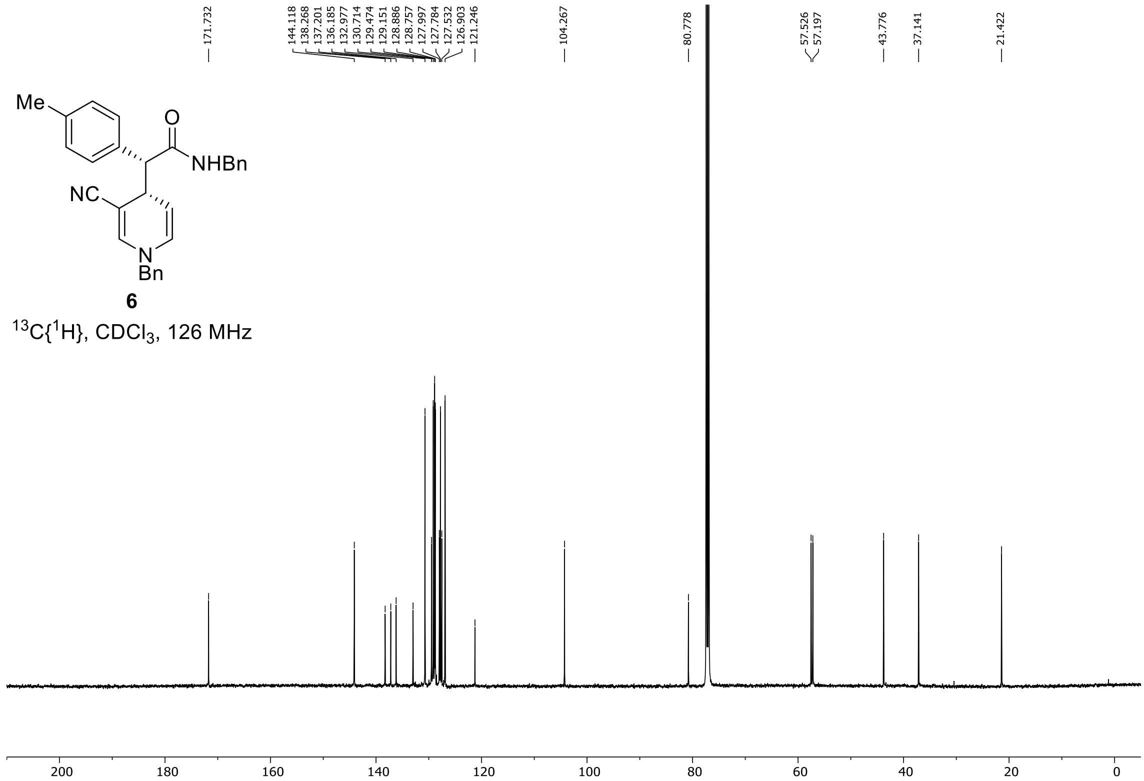

Me
O
NHBn
NC
N
Bn
6
^{13}C{^{1}H}, CDCl$_3$, 126 MHz
171.732 144.118 138.268 137.201 136.185 132.977 130.714 129.474 129.151 128.886 128.757 127.997 127.784 127.532 126.903 121.246 104.267 80.778 57.526 57.197 43.776 37.141 21.422
f1 (ppm)

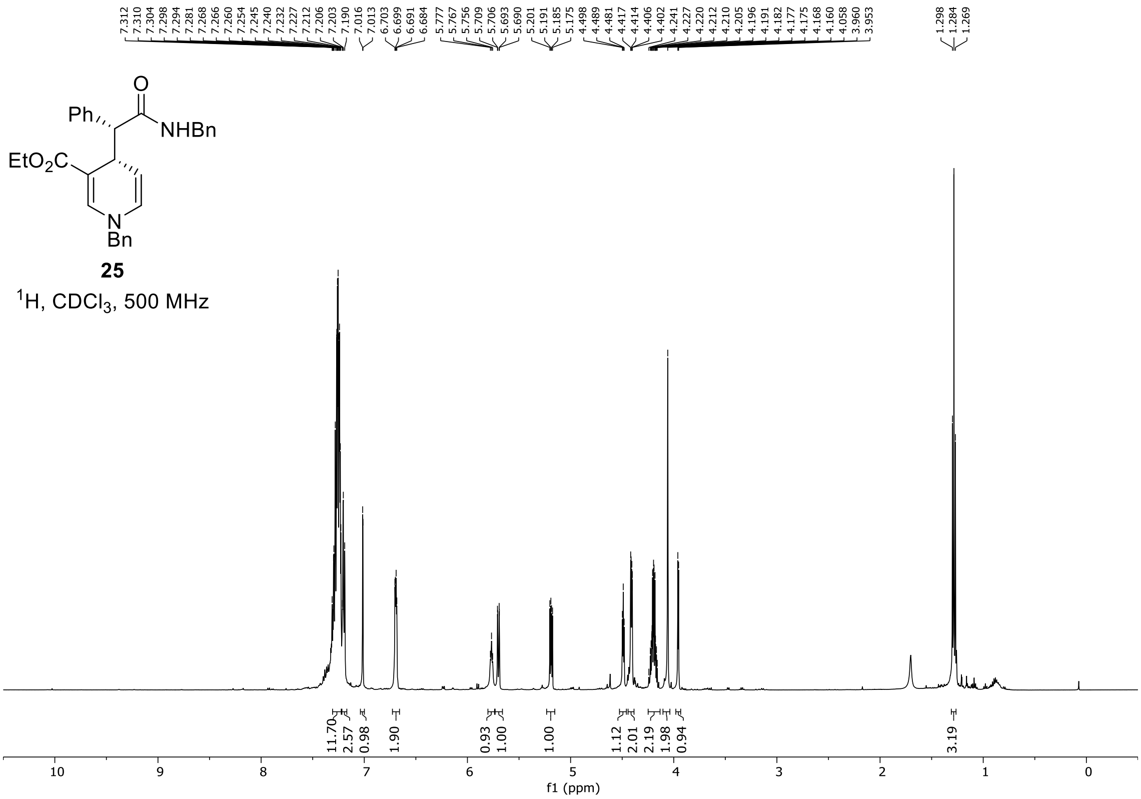

25

^{1}H, $CDCl_3$, 500 MHz

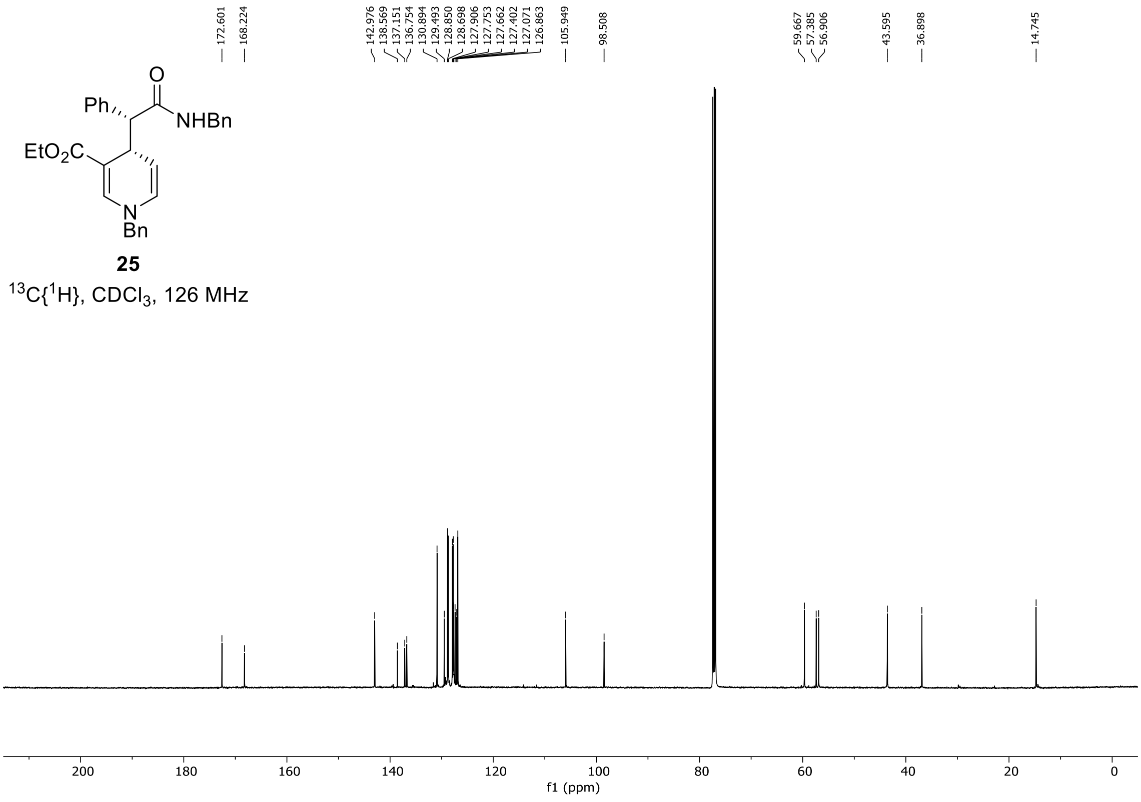

25

$^{13}C\{^{1}H\}$, $CDCl_3$, 126 MHz

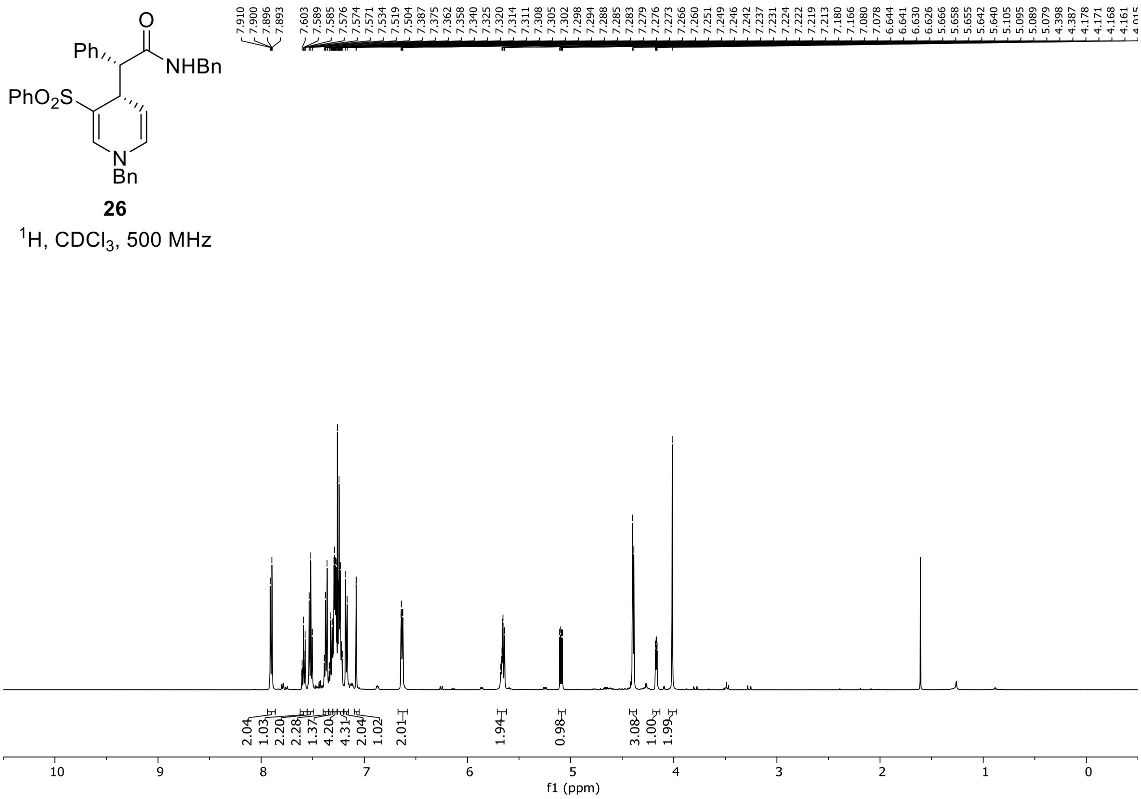

26
^1H, $CDCl_3$, 500 MHz

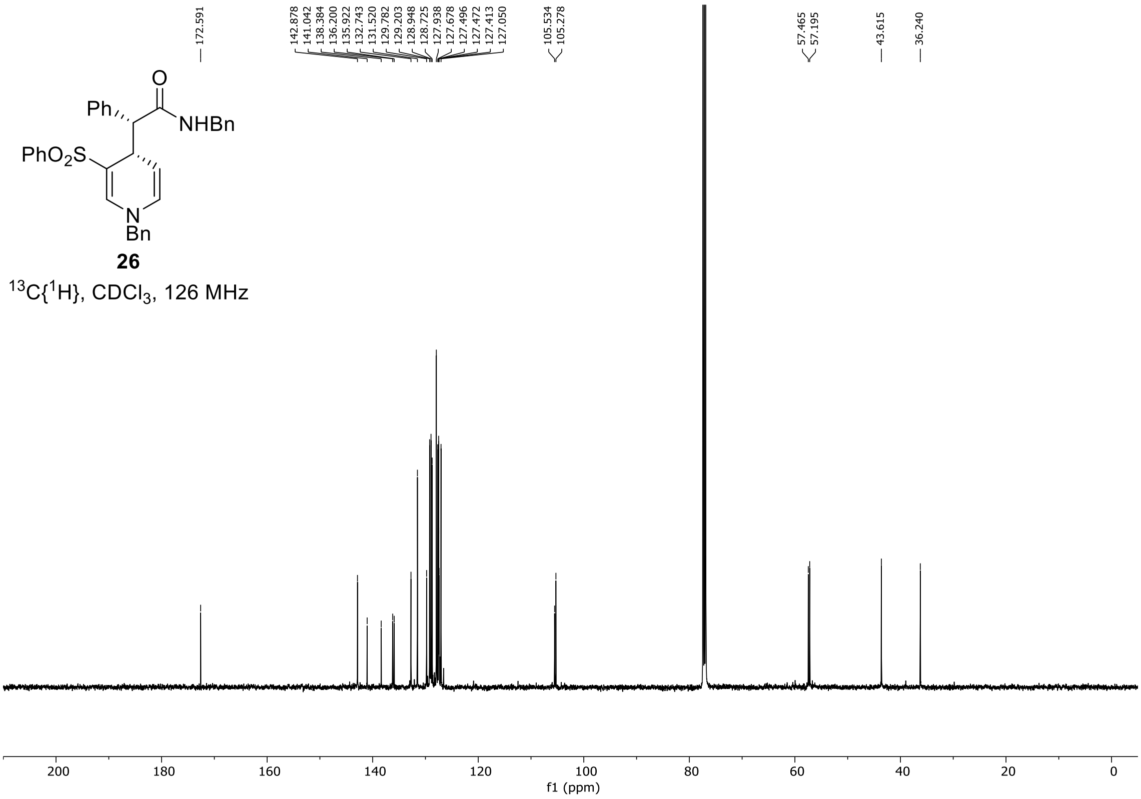

26
$^{13}C\{^1H\}$, $CDCl_3$, 126 MHz

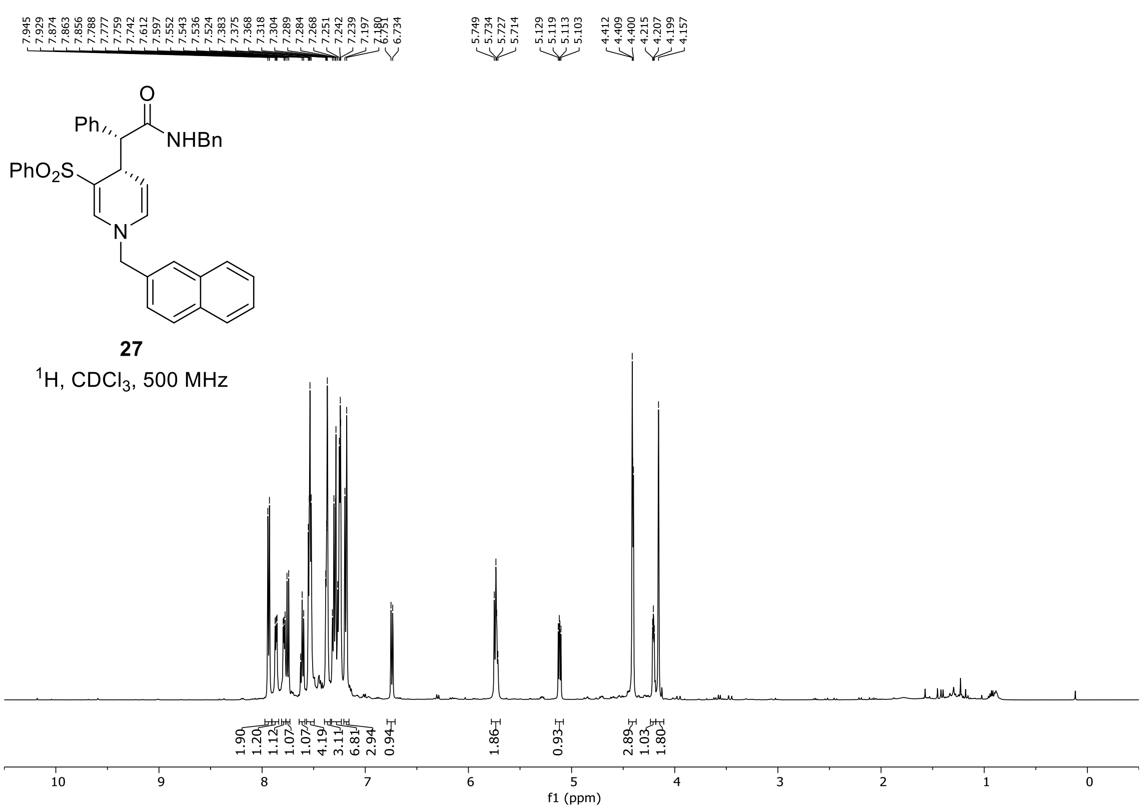
7.945 7.929 7.874 7.863 7.856 7.788 7.777 7.759 7.742 7.612 7.597 7.552 7.543 7.536 7.524 7.383 7.375 7.368 7.318 7.304 7.289 7.284 7.268 7.251 7.242 7.239 7.197 7.180 6.751 6.734
5.749 5.734 5.727 5.714
5.129 5.119 5.113 5.103
4.412 4.409 4.400 4.215 4.207 4.199 4.157
Ph
O
NHBn
PhO2S
N
27
1H, CDCl3, 500 MHz
1.90 1.20 1.12 1.07 1.07 4.19 3.11 6.81 2.94 0.94
1.86
0.93
2.89 1.03 1.80
f1 (ppm)

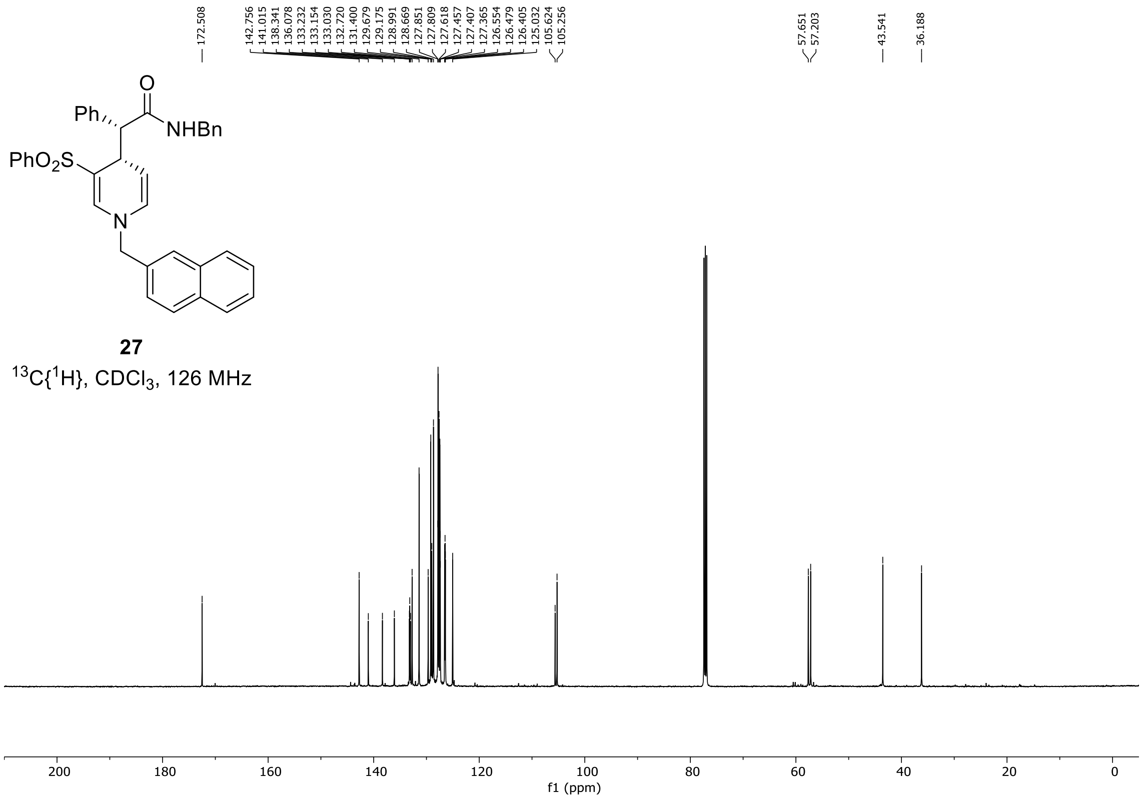
172.508
142.756 141.015 138.341 136.078 133.232 133.154 133.030 132.720 131.400 129.679 129.175 128.991 128.669 127.851 127.809 127.618 127.457 127.407 127.365 126.554 126.479 126.405 125.032 105.624 105.256
57.651 57.203
43.541
36.188
Ph
O
NHBn
PhO2S
N
27
13C{1H}, CDCl3, 126 MHz
f1 (ppm)

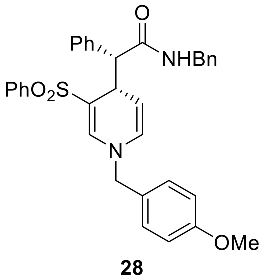

28

^{1}H, $CDCl_3$, 400 MHz

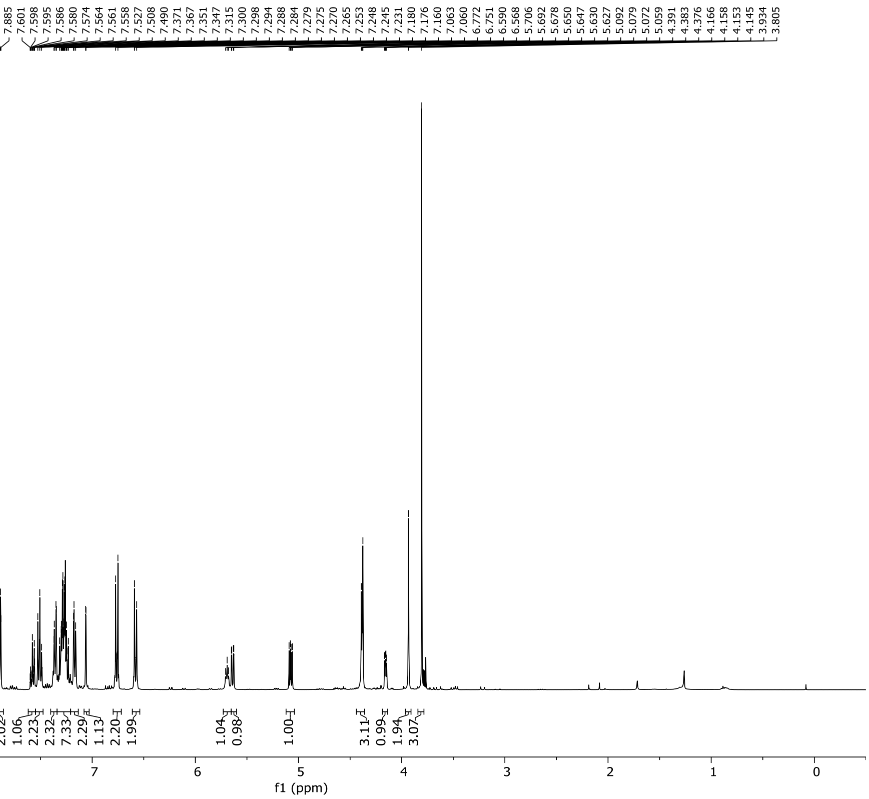

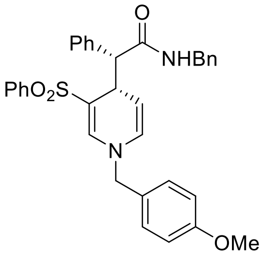

28

$^{13}C\{^{1}H\}$, $CDCl_3$, 101 MHz

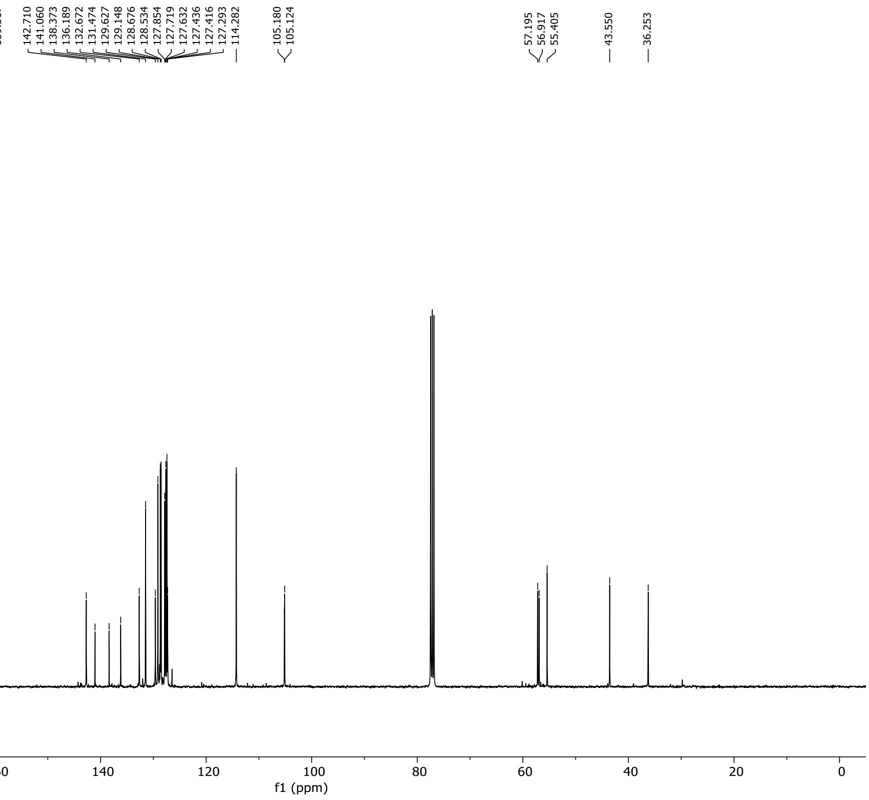

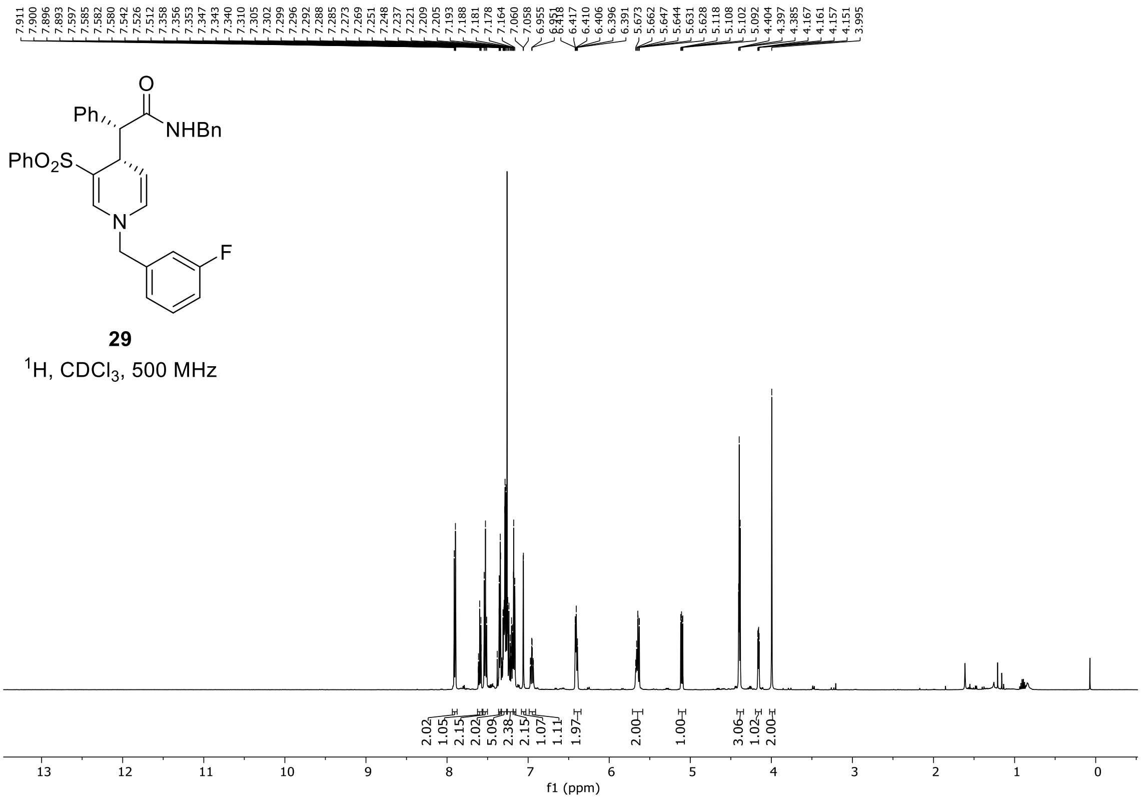

29

^{1}H, $CDCl_3$, 500 MHz

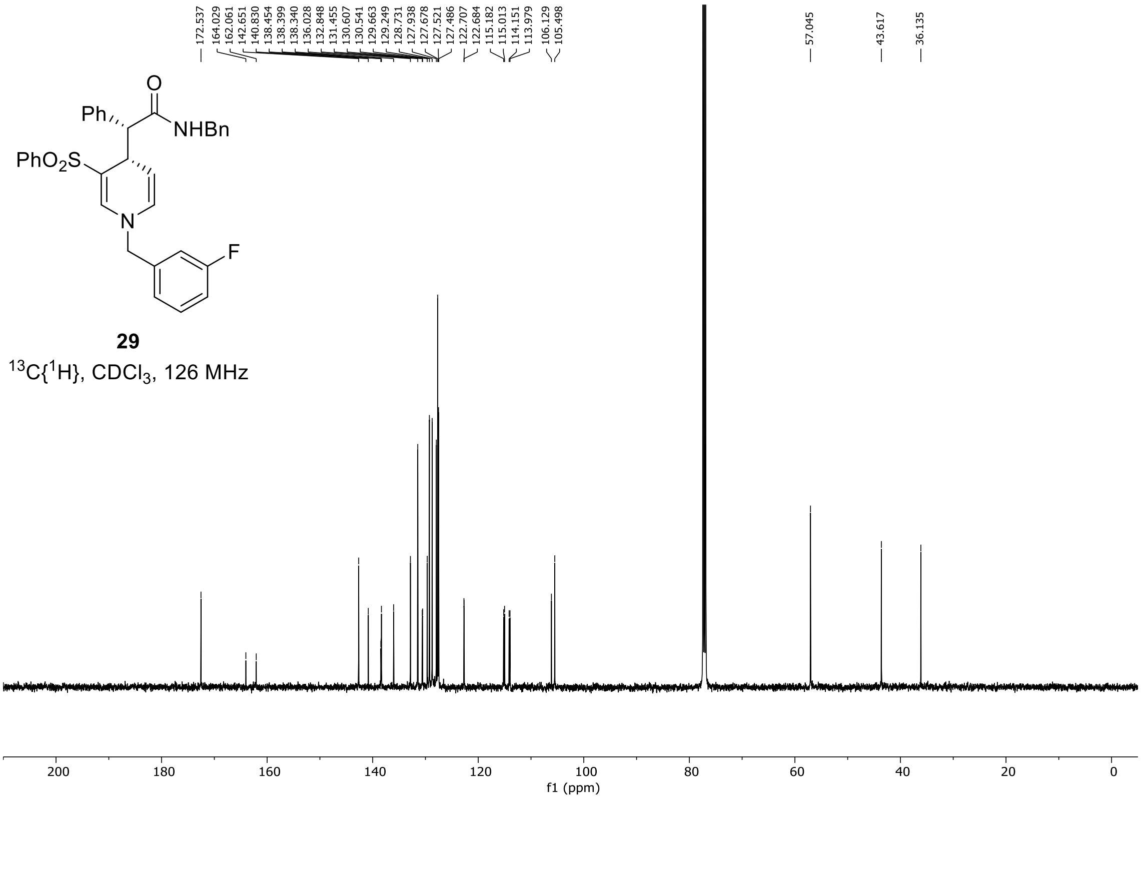

29

$^{13}C\{^1H\}$, $CDCl_3$, 126 MHz

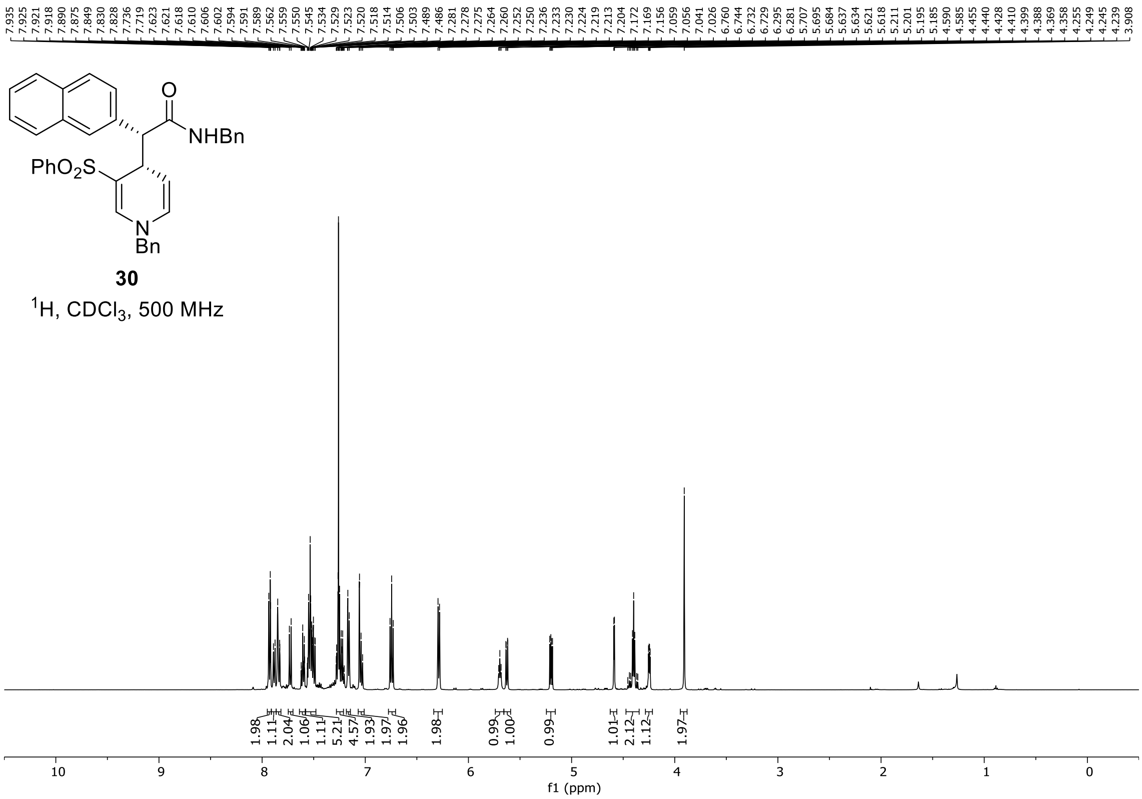

30

^1H, $CDCl_3$, 500 MHz

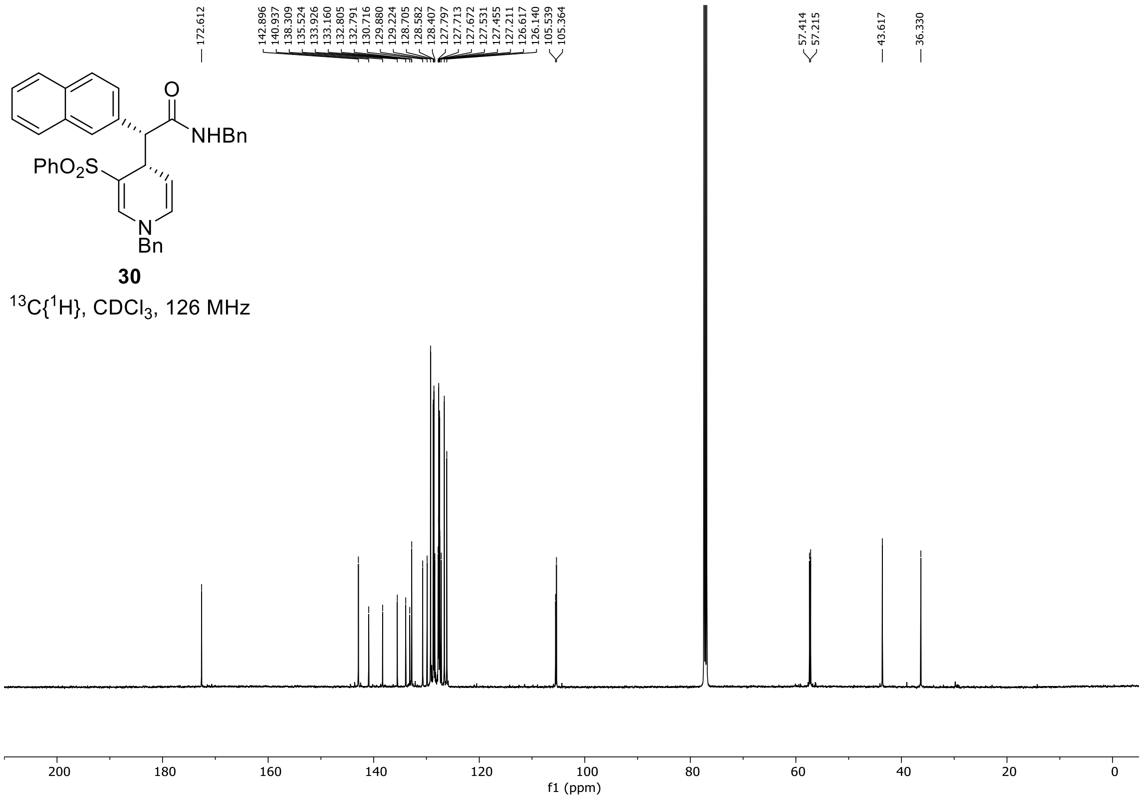

30

$^{13}C\{^1H\}$, $CDCl_3$, 126 MHz

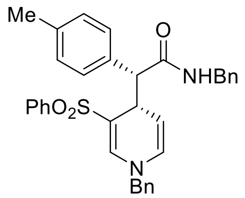

31

^{1}H, $CDCl_3$, 500 MHz

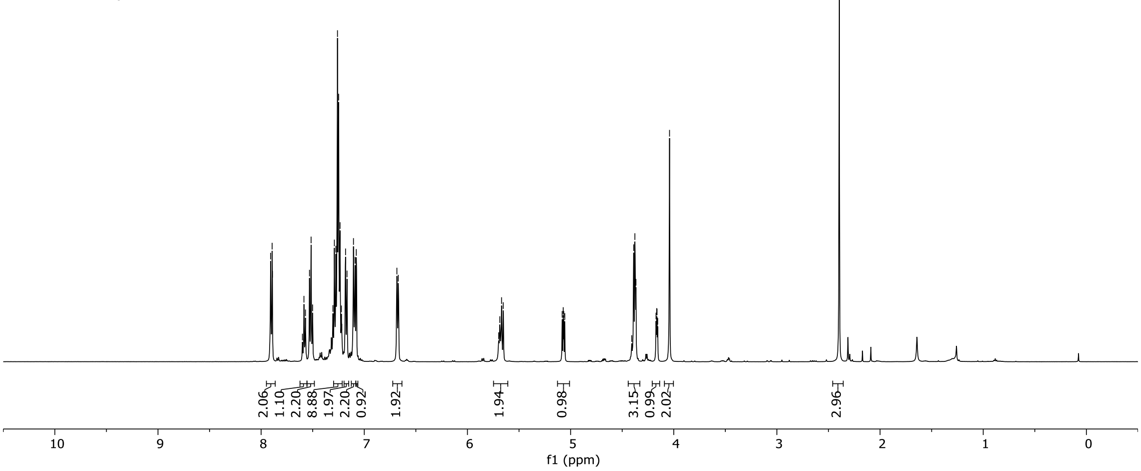

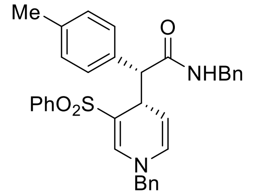

31

^{13}C{^{1}H}, $CDCl_3$, 126 MHz

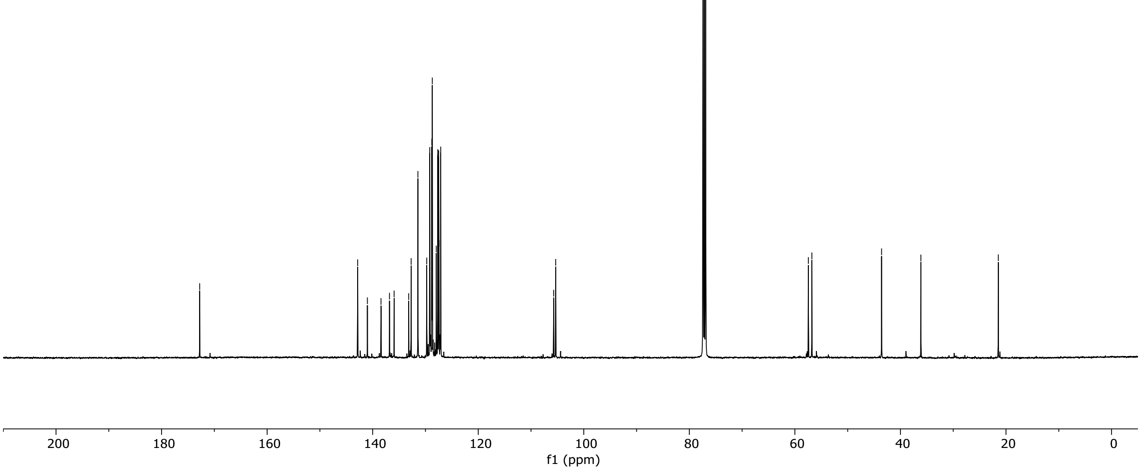

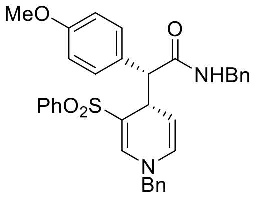

32

^{1}H, $CDCl_3$, 500 MHz

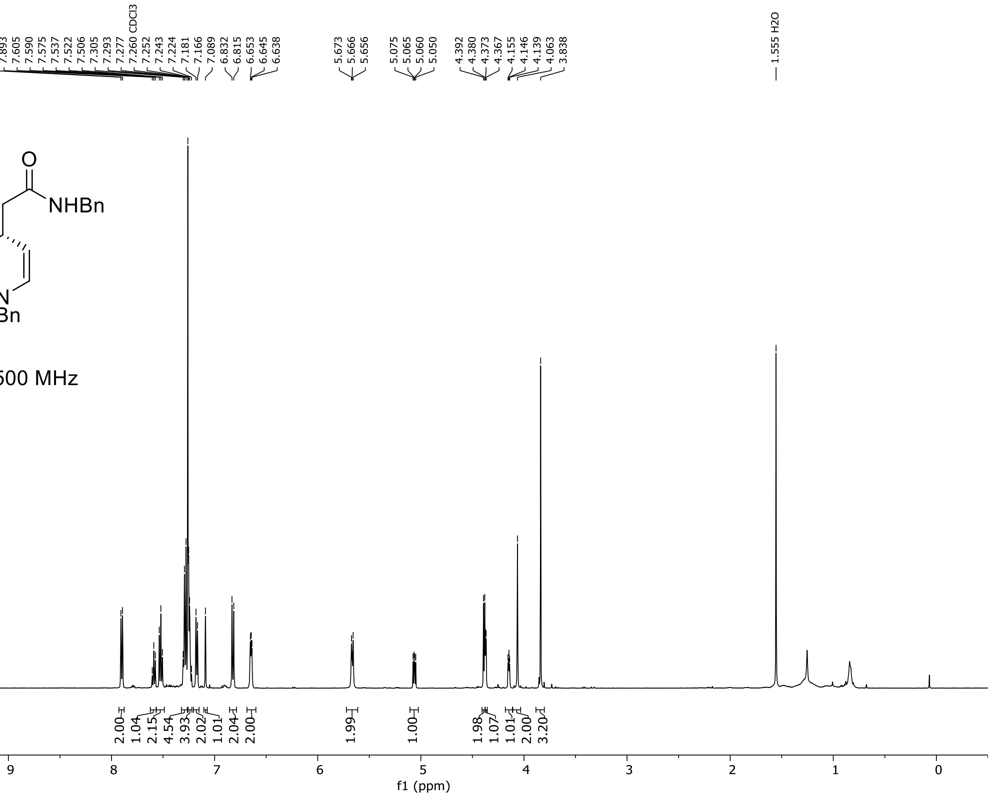

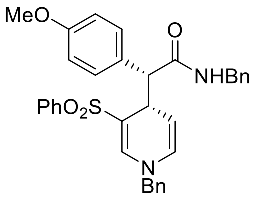

32

$^{13}C\{^1H\}$, $CDCl_3$, 126 MHz

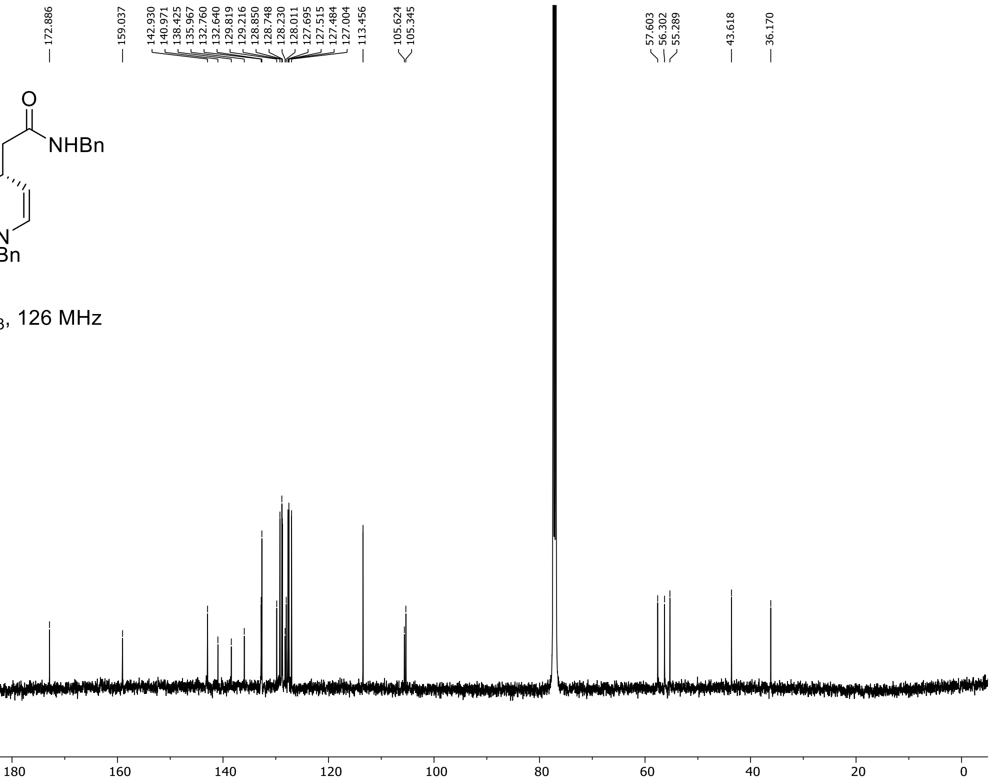

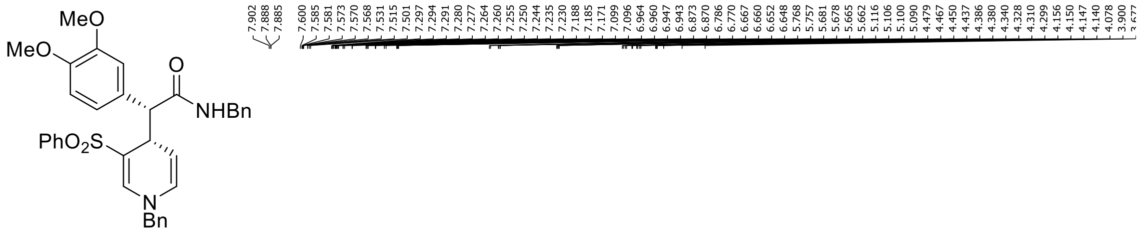

33

^1H, $CDCl_3$, 500 MHz

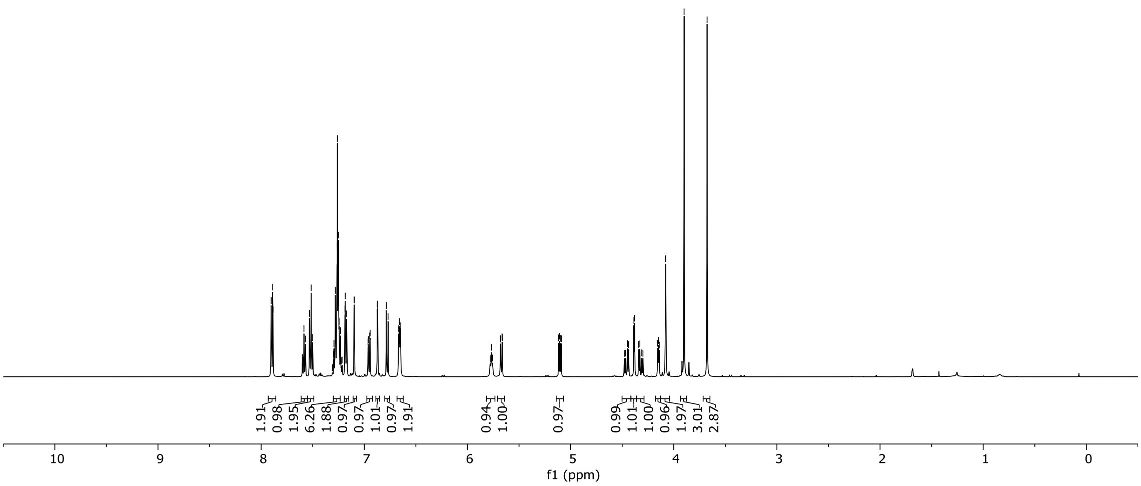

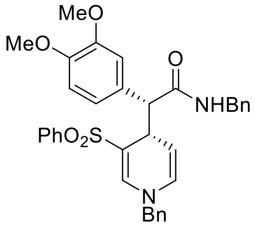

33

$^{13}C\{^1H\}$, $CDCl_3$, 126 MHz

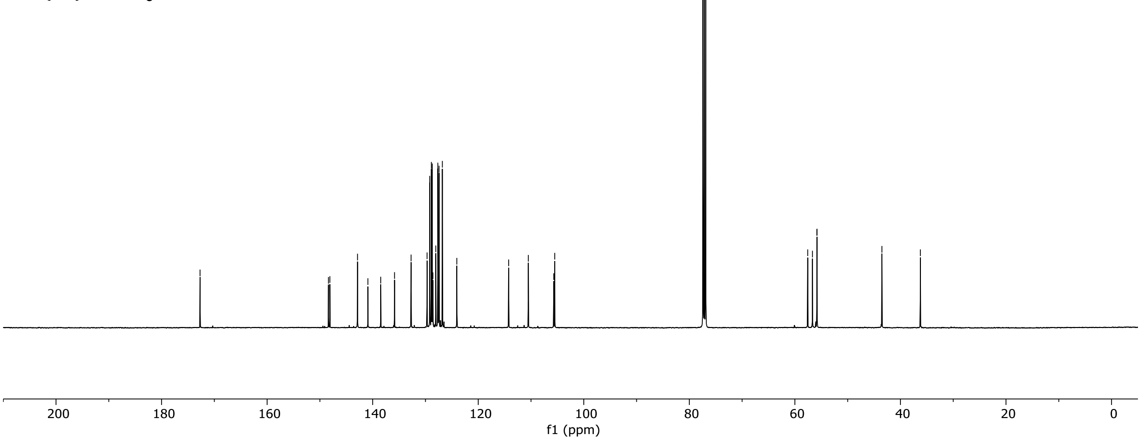

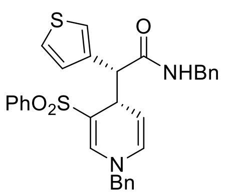

34

^1H, $CDCl_3$, 500 MHz

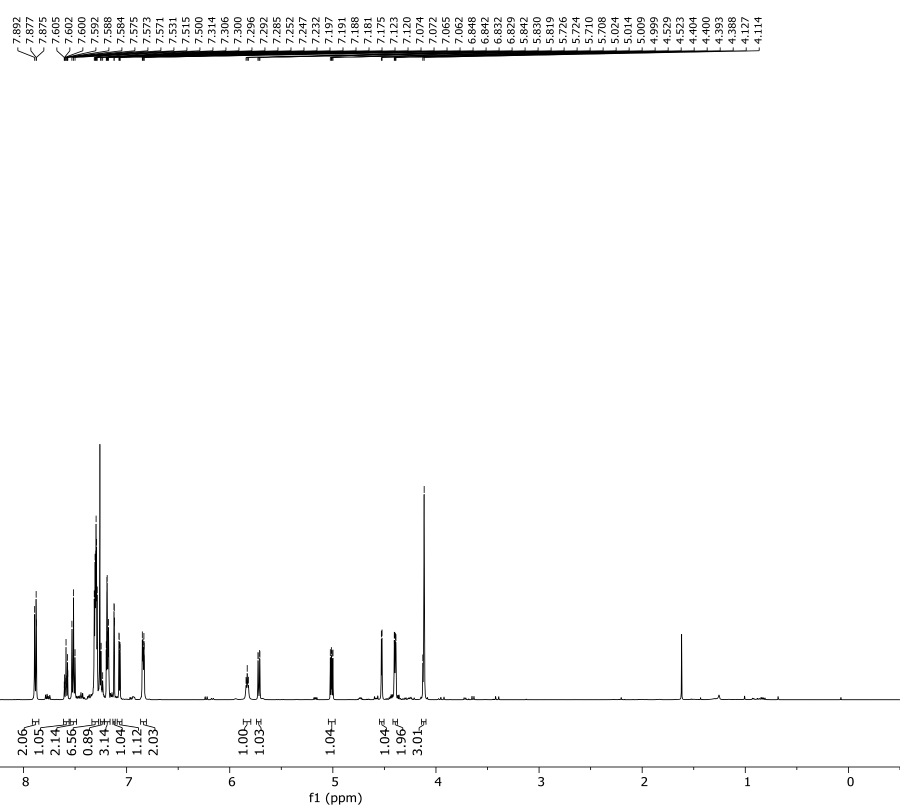

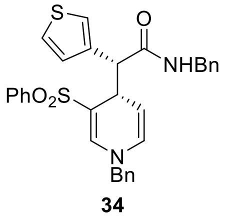

34

$^{13}C\{^1H\}$, $CDCl_3$, 126 MHz

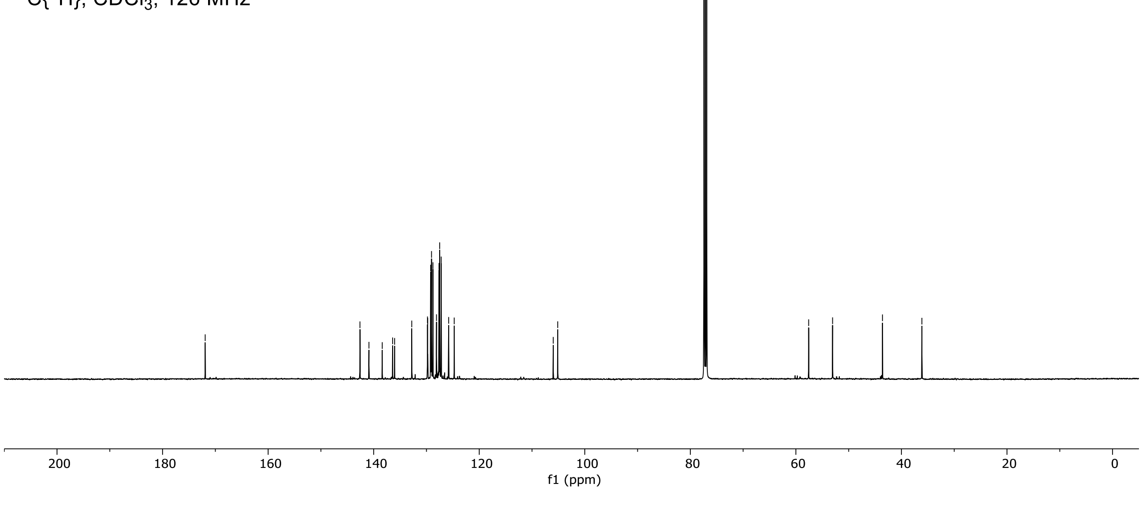

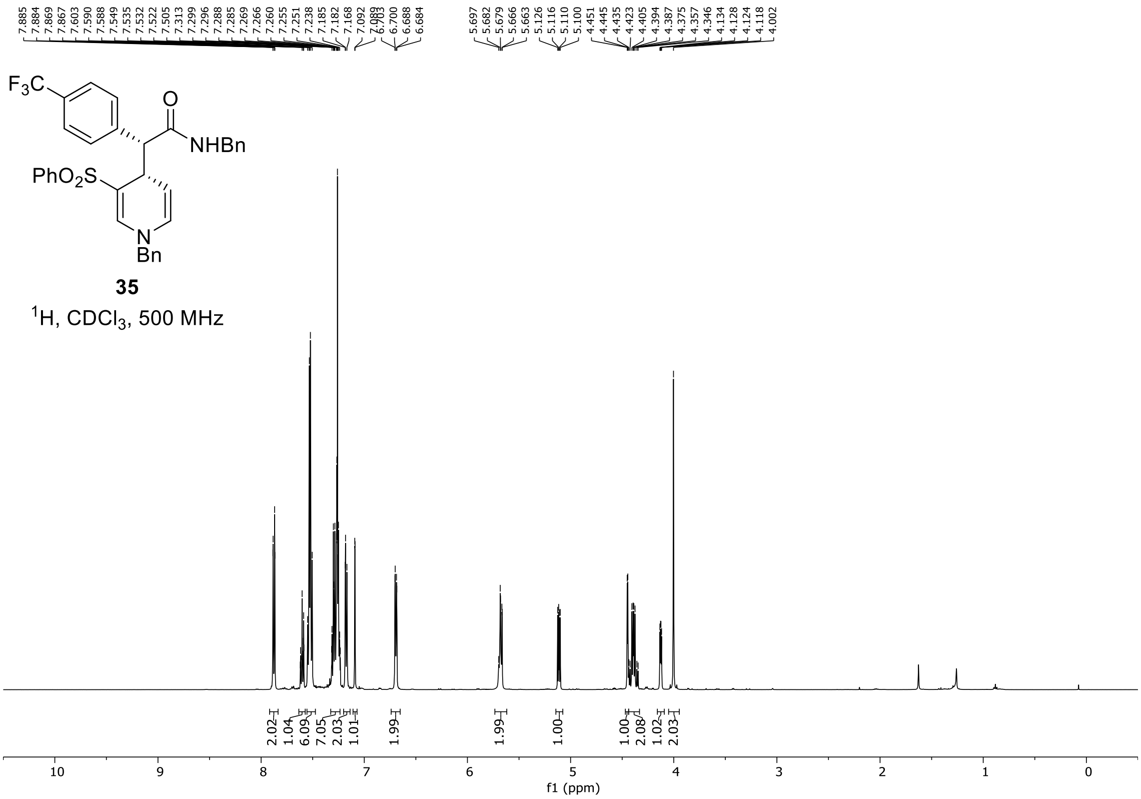

35

^{1}H, $CDCl_3$, 500 MHz

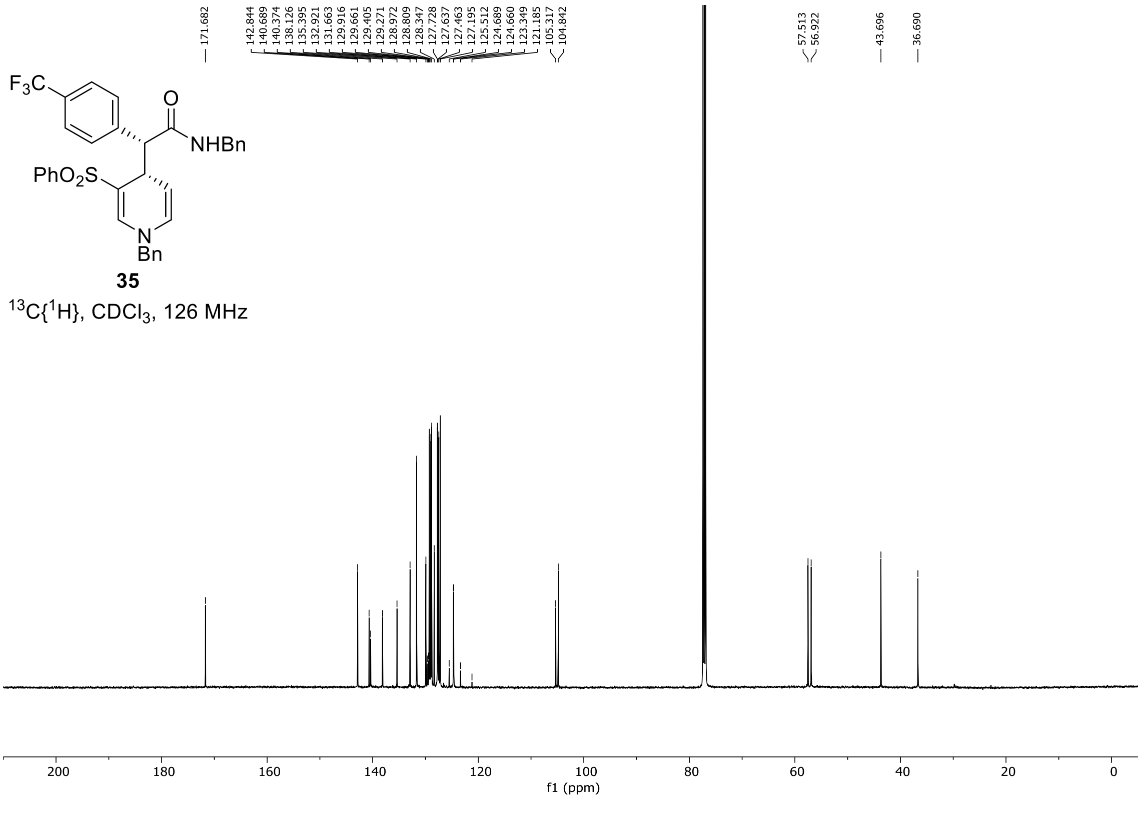

35

$^{13}C\{^{1}H\}$, $CDCl_3$, 126 MHz

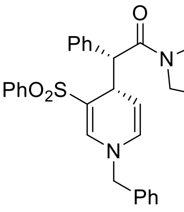

36

^{1}H, $CDCl_3$, 500 MHz

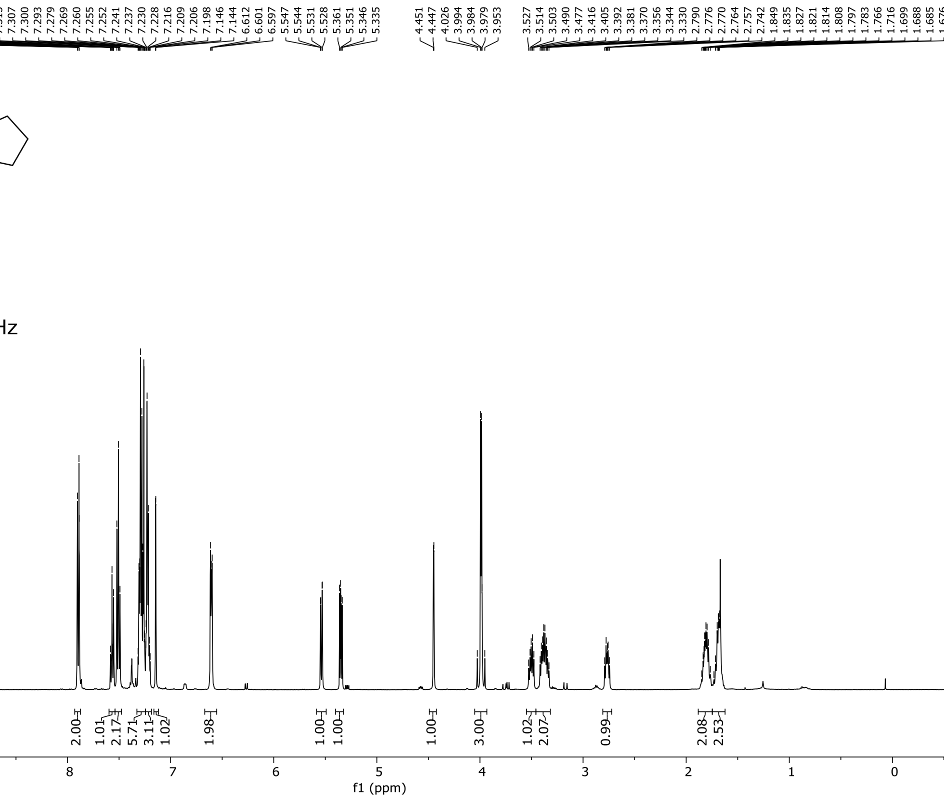

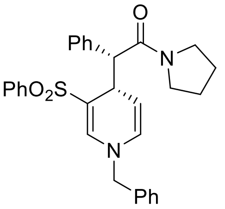

36

$^{13}C\{^{1}H\}$, $CDCl_3$, 126 MHz

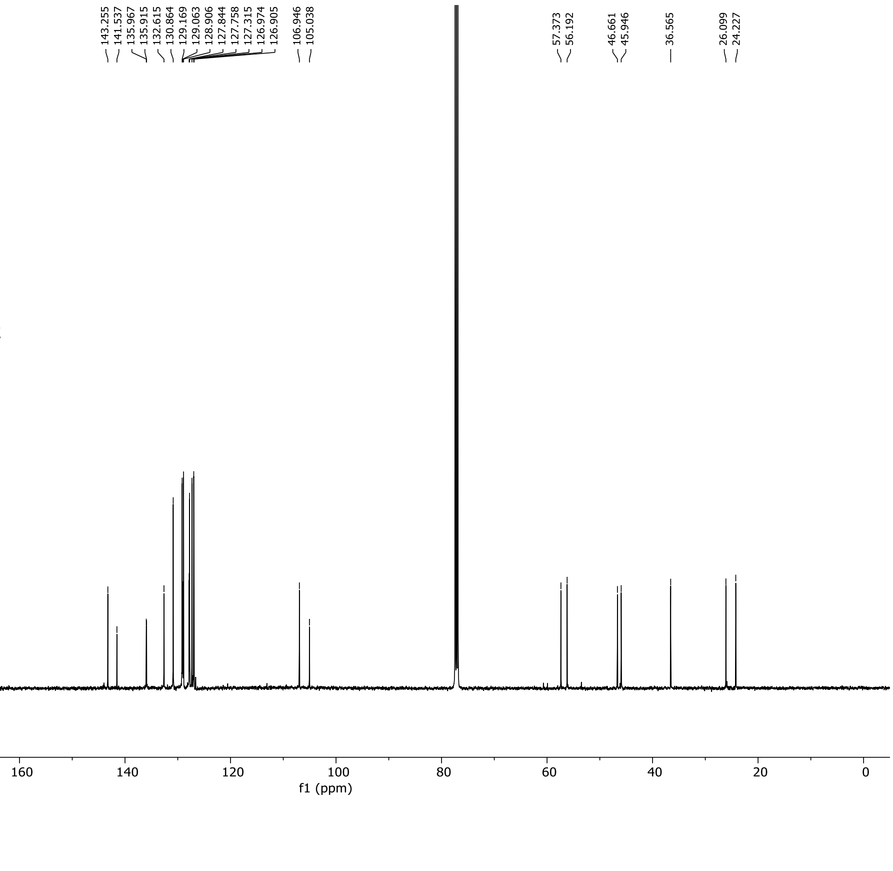

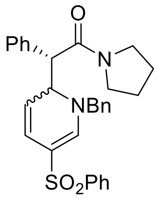

S2

^{1}H, $CDCl_3$, 500 MHz

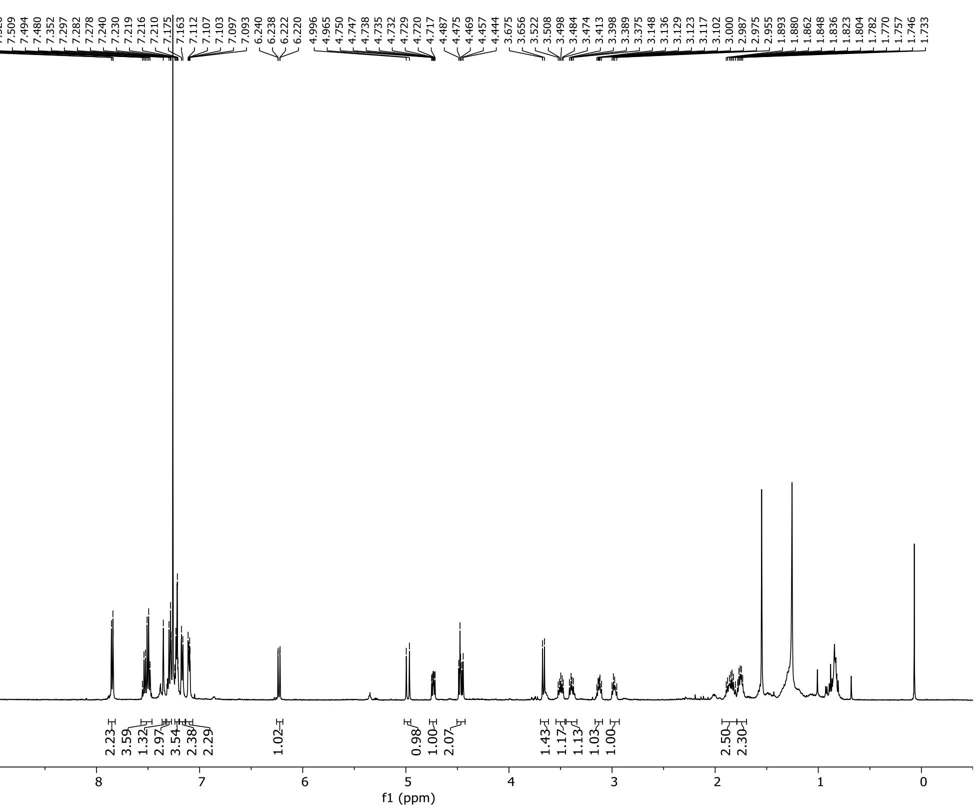

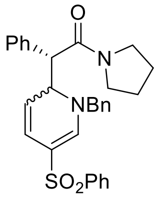

S2

$^{13}C\{^{1}H\}$, $CDCl_3$, 126 MHz

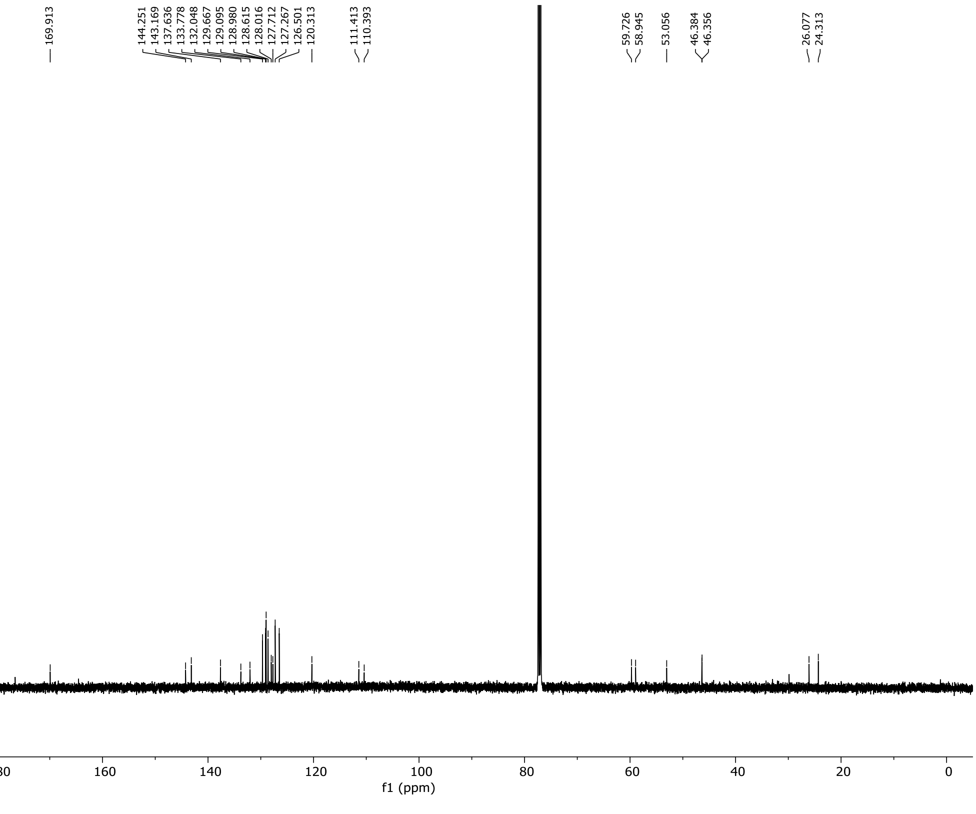

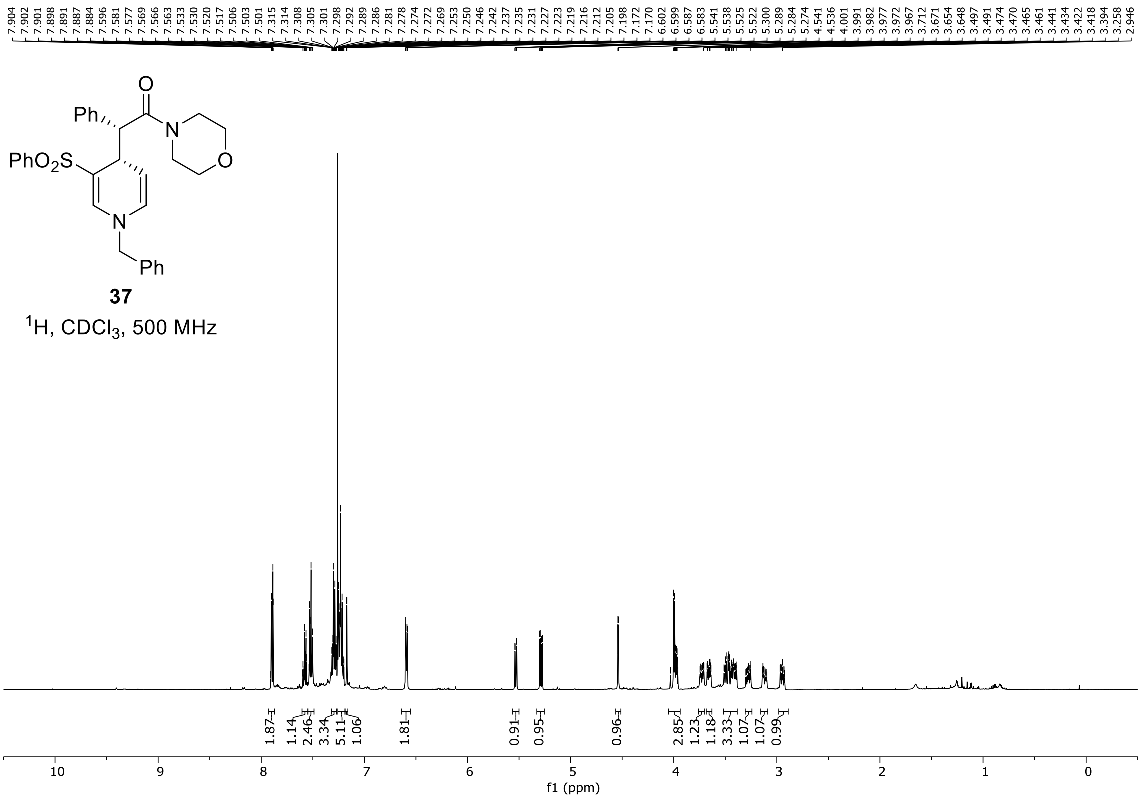

37

^{1}H, $CDCl_3$, 500 MHz

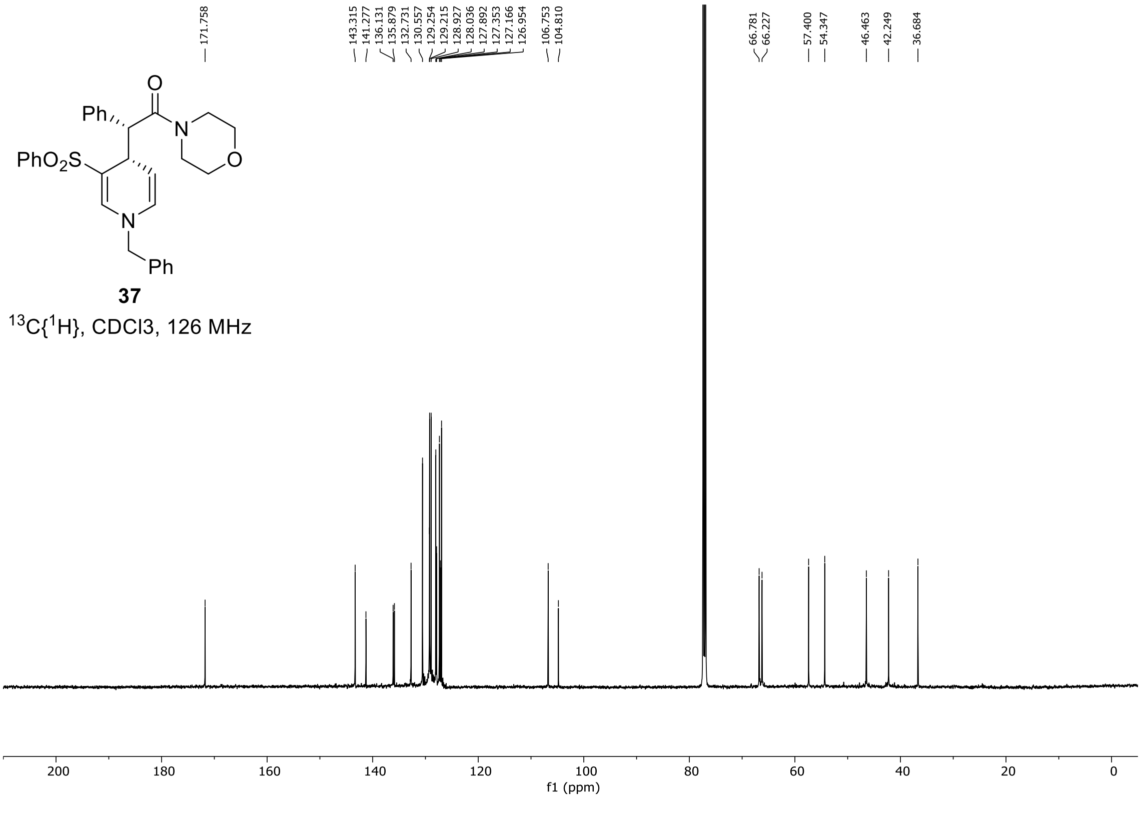

37

$^{13}C\{^{1}H\}$, CDCl3, 126 MHz

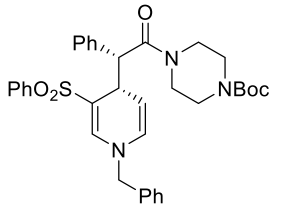

38

^{1}H, CD_3CN, 500 MHz

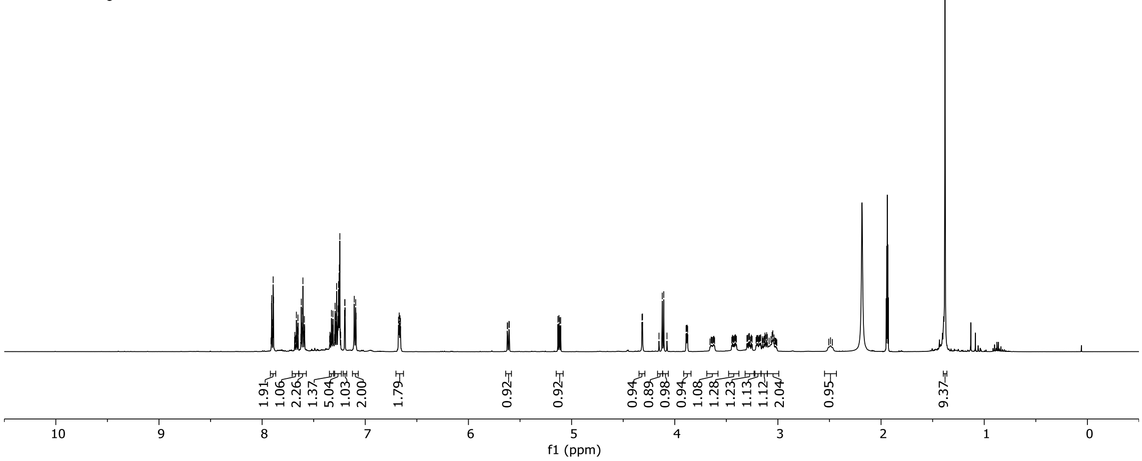

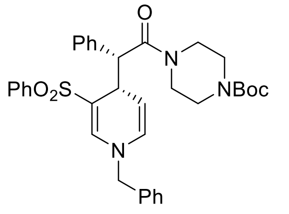

38

$^{13}C\{^{1}H\}$, CD_3CN, 126 MHz

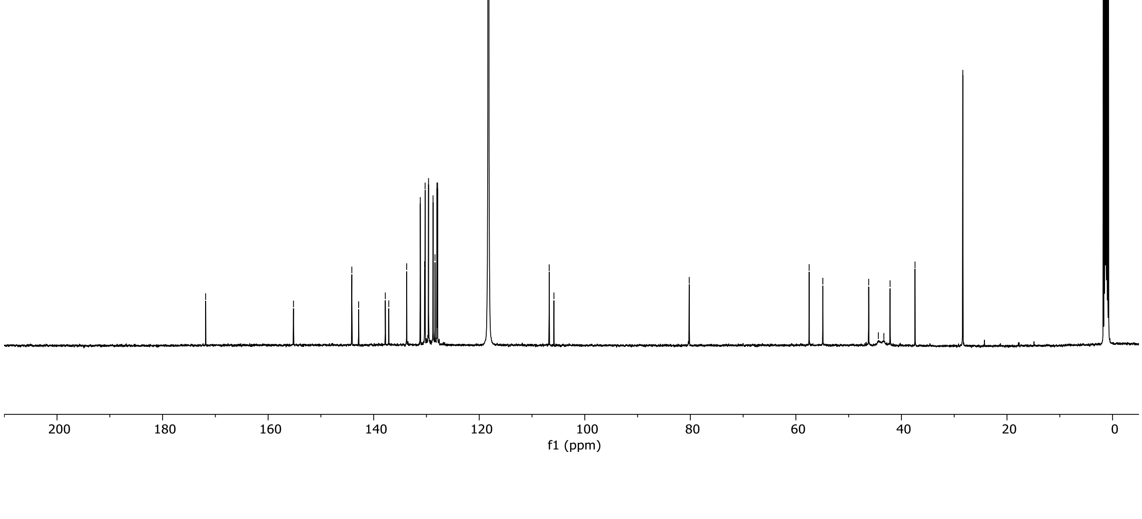

11. Appendix II. HPLC traces

HPLC Data for **6**: Chiralpak AD-H, (85:15 hexane: IPA, flow rate 1.0 mLmin^{-1}, 211 nm, 30 °C) t_R (minor): 25.5 min, t_R (major): 31.7 min, 91:9 er

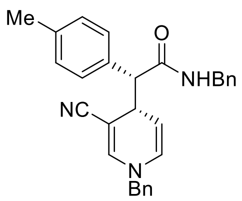

6

<Chromatogram>

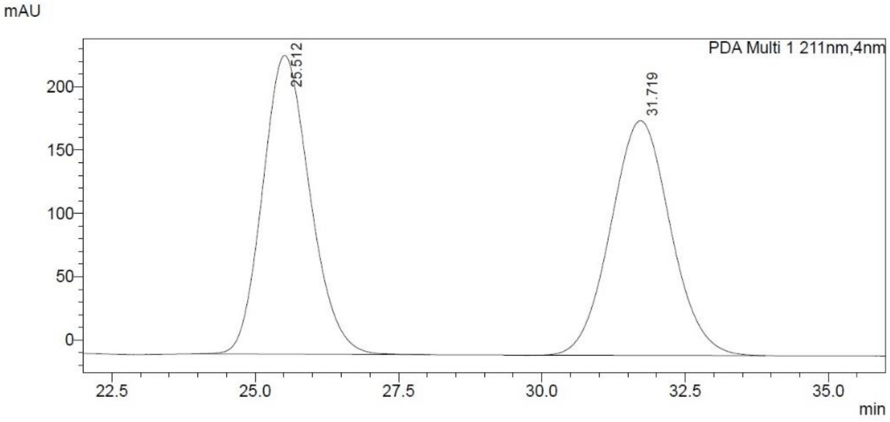

<Peak Table>

PDA Ch1 211nm

Peak#	Ret. Time	Area%
1	25.512	50.137
2	31.719	49.863
Total		100.000

<Chromatogram>

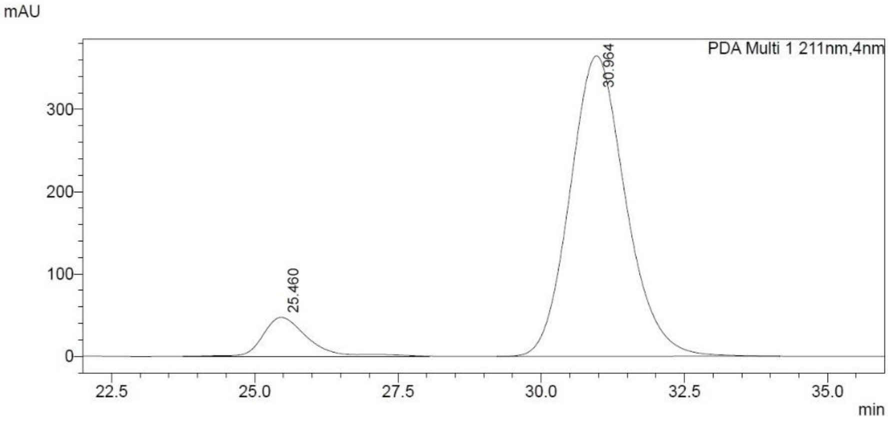

<Peak Table>

PDA Ch1 211nm

Peak#	Ret. Time	Area%
1	25.460	9.327
2	30.964	90.673
Total		100.000

HPLC Data for **6** starting from chloride salt **7**: Chiralpak AD-H, (85:15 hexane: IPA, flow rate 1.0 $mLmin^{-1}$, 211 nm, 30 °C) t_R (minor): 25.5 min, t_R (major): 31.7 min, 91:9 er

<Chromatogram>

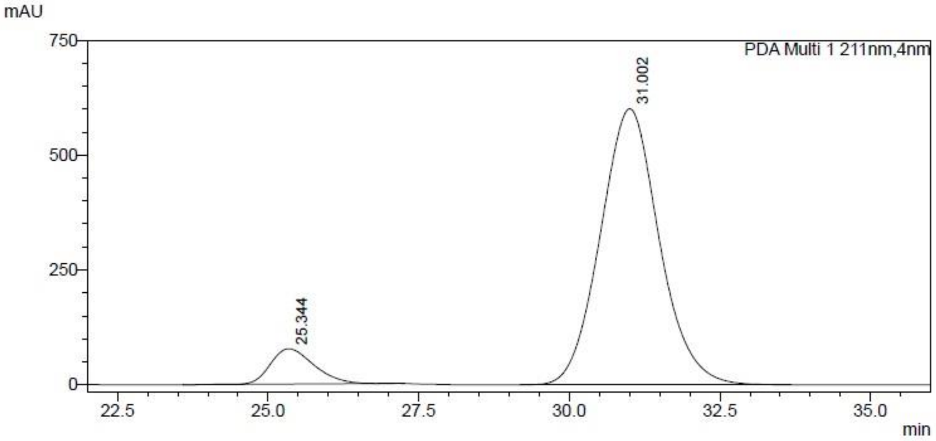

<Peak Table>

PDA Ch1 211nm

Peak#	Ret. Time	Area%
1	25.344	8.723
2	31.002	91.277
Total		100.000

HPLC Data for **6** starting from PF_6 salt **8**: Chiralpak AD-H, (85:15 hexane: IPA, flow rate 1.0 $mLmin^{-1}$, 211 nm, 30 °C) t_R (minor): 25.5 min, t_R (major): 31.7 min, 83:17 er

<Chromatogram>

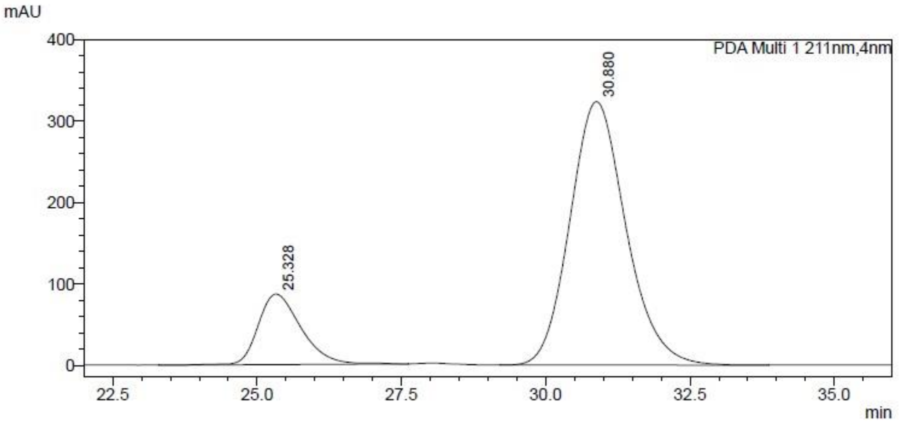

<Peak Table>

PDA Ch1 211nm

Peak#	Ret. Time	Area%
1	25.328	17.073
2	30.880	82.927
Total		100.000

HPLC Data for **6** starting from BF_4 salt **9**: Chiralpak AD-H, (85:15 hexane: IPA, flow rate 1.0 mLmin^{-1}, 211 nm, 30 °C) t_R (minor): 25.5 min, t_R (major): 31.7 min, 84:16 er

<Chromatogram>

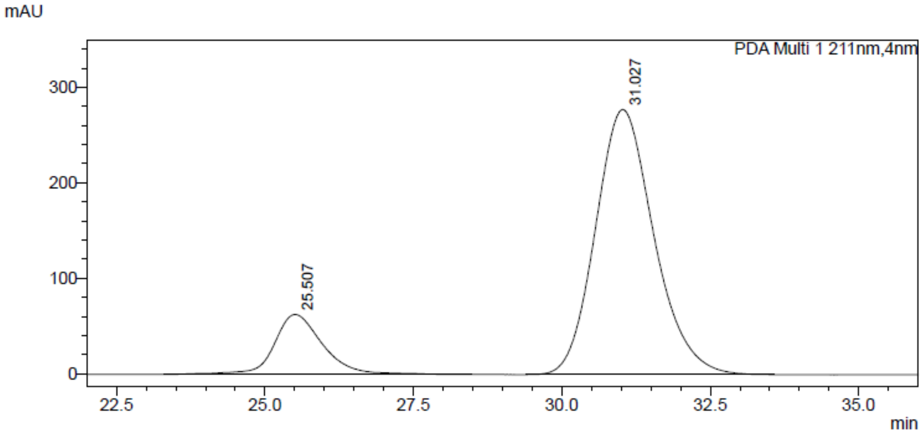

<Peak Table>

PDA Ch1 211nm

Peak#	Ret. Time	Area%
1	25.507	15.949
2	31.027	84.051
Total		100.000

HPLC Data for **25**: Chiralcel OD-H, (80:20 hexane: IPA, flow rate 1.0 mLmin^{-1}, 211 nm, 30 °C) t$_R$: 10.2 min and 18.0 min, 51:49 er

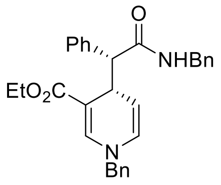

25

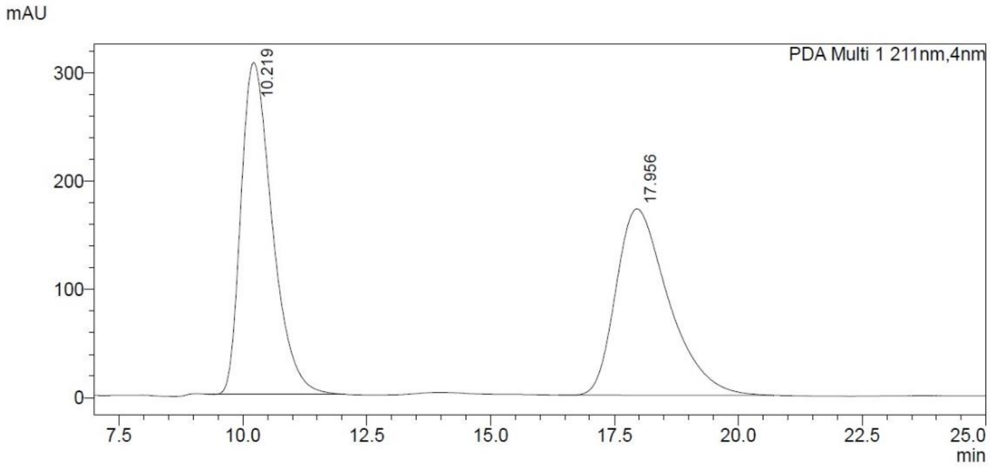

<Peak Table>

PDA Ch1 211nm

Peak#	Ret. Time	Area%
1	10.219	51.353
2	17.956	48.647
Total		100.000

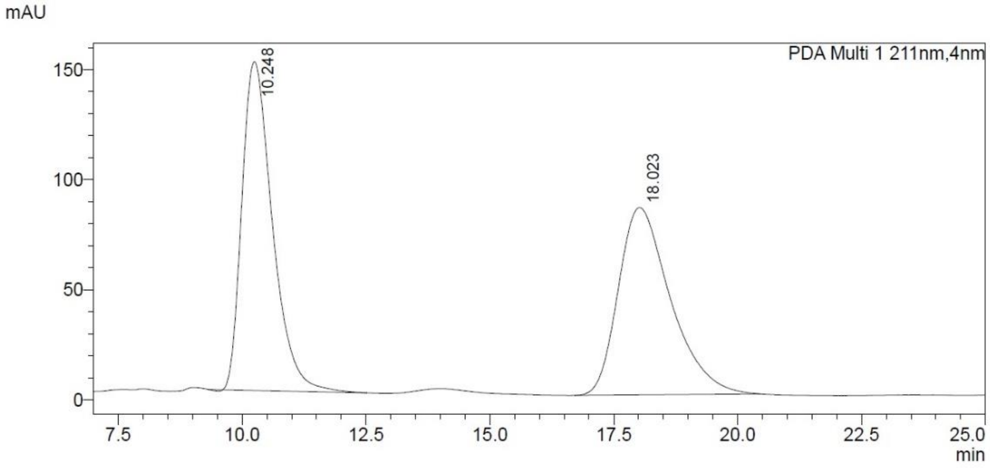

<Peak Table>

PDA Ch1 211nm

Peak#	Ret. Time	Area%
1	10.248	50.603
2	18.023	49.397
Total		100.000

HPLC Data for **26**: Chiralpak IB, (80:20 hexane: IPA, flow rate 1.0 mLmin^{-1}, 211 nm, 30 °C) t_R (major): 12.2 min, t_R (minor): 14.6 min, 94:6 er

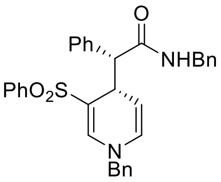

26

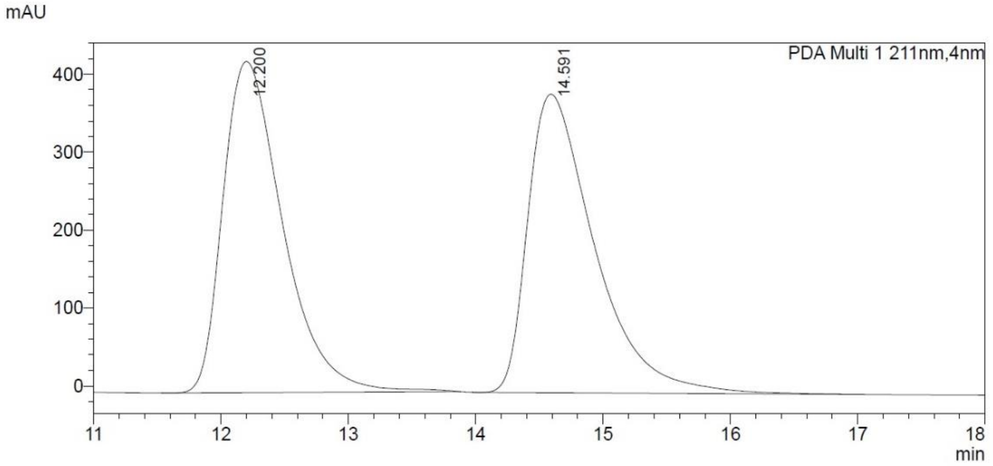

<Peak Table>

PDA Ch1 211nm

Peak#	Ret. Time	Area%
1	12.200	49.921
2	14.591	50.079
Total		100.000

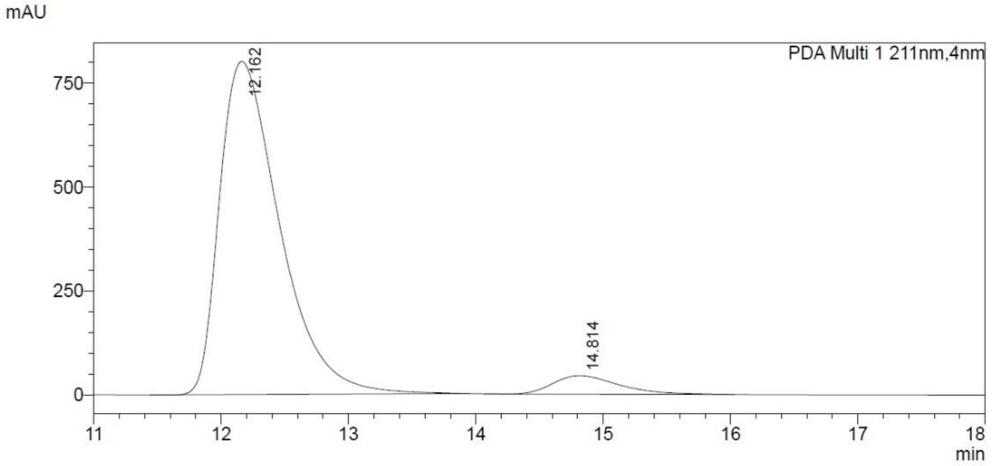

<Peak Table>

PDA Ch1 211nm

Peak#	Ret. Time	Area%
1	12.162	94.278
2	14.814	5.722
Total		100.000

HPLC Data for **27**: Chiralpak IB, (80:20 hexane: IPA, flow rate 1.0 mLmin^{-1}, 211 nm, 30 °C) t_R (major): 16.8 min, t_R (minor): 20.8 min, 94:6 er

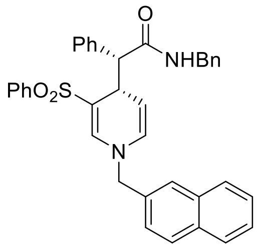

27

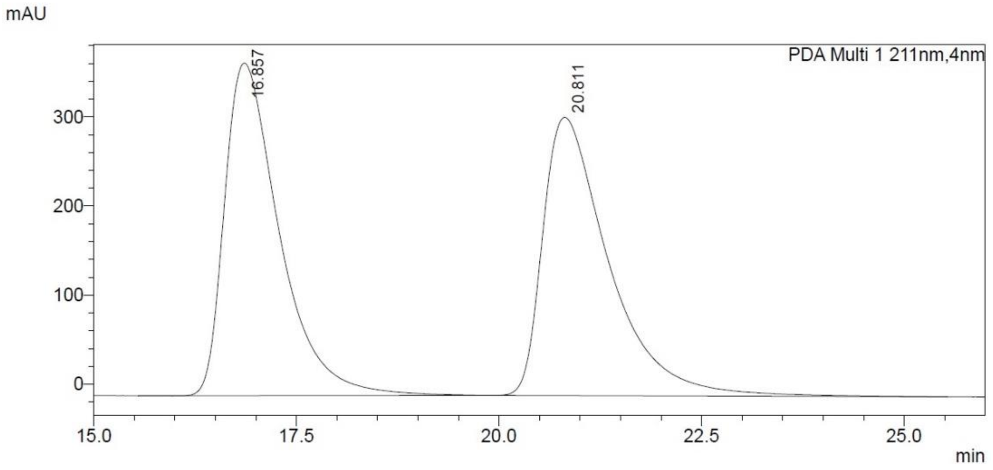

<Peak Table>

PDA Ch1 211nm

Peak#	Ret. Time	Area%
1	16.857	49.804
2	20.811	50.196
Total		100.000

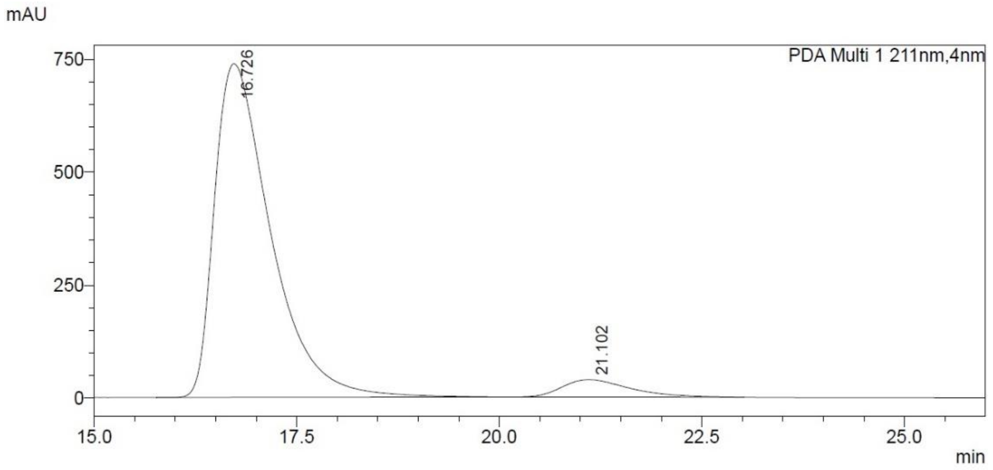

<Peak Table>

PDA Ch1 211nm

Peak#	Ret. Time	Area%
1	16.726	94.081
2	21.102	5.919
Total		100.000

HPLC Data for **28**: Chiralpak IB, (80:20 hexane: IPA, flow rate 1.0 mLmin⁻¹, 211 nm, 30 °C) t_R (major): 14.8 min, t_R (minor): 17.4 min, 91:9 er

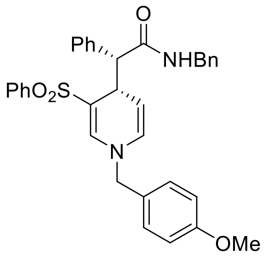

28

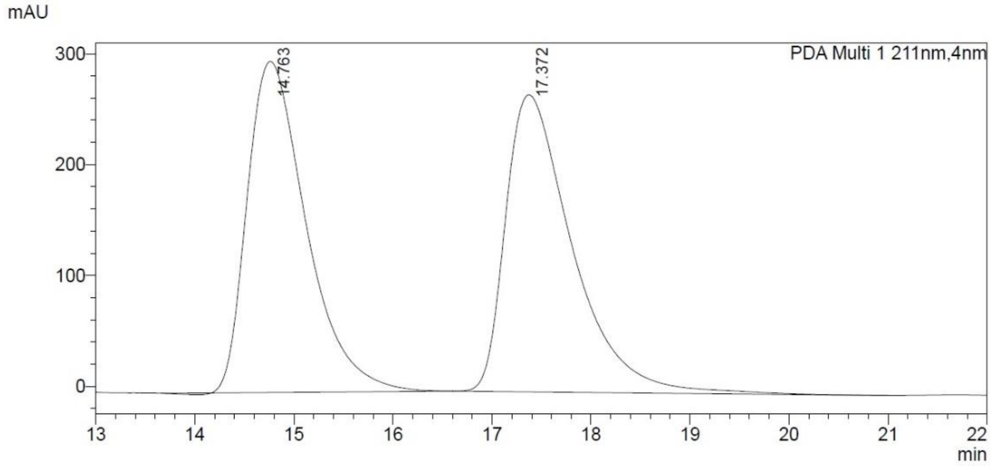

<Peak Table>

PDA Ch1 211nm

Peak#	Ret. Time	Area%
1	14.763	49.587
2	17.372	50.413
Total		100.000

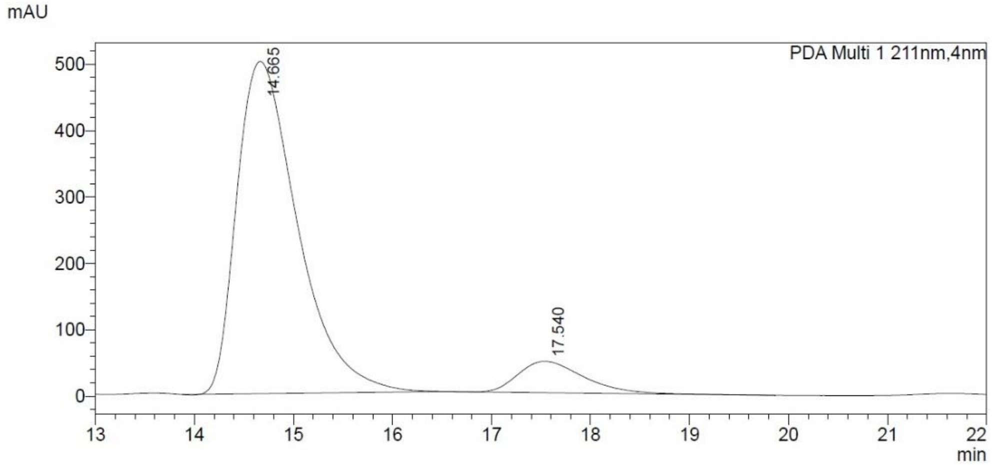

<Peak Table>

PDA Ch1 211nm

Peak#	Ret. Time	Area%
1	14.665	90.758
2	17.540	9.242
Total		100.000

HPLC Data for **29**: Chiralcel OD-H, (80:20 hexane: IPA, flow rate 1.0 mLmin^{-1}, 220 nm, 30 °C) t_R (major): 14.5 min, t_R (minor): 18.9 min, 93:7 er

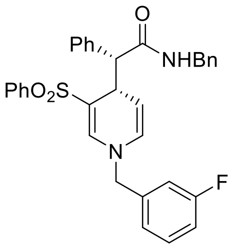

29

<Chromatogram>

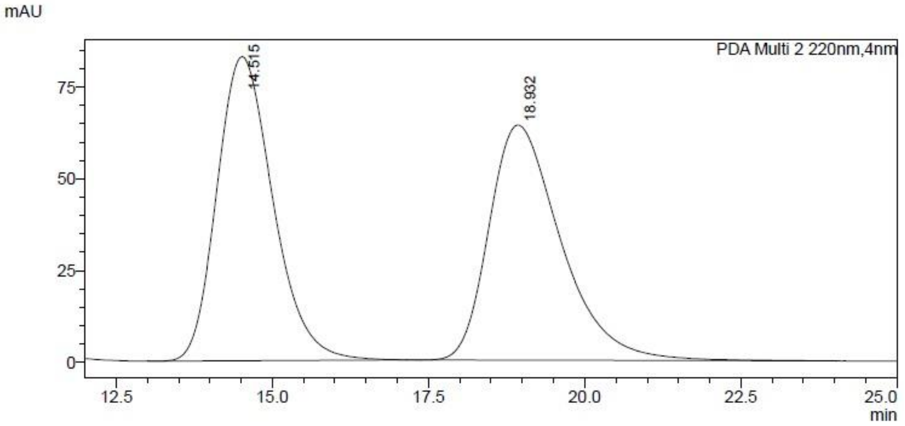

<Peak Table>

PDA Ch2 220nm

Peak#	Ret. Time	Area%
1	14.515	50.244
2	18.932	49.756
Total		100.000

<Chromatogram>

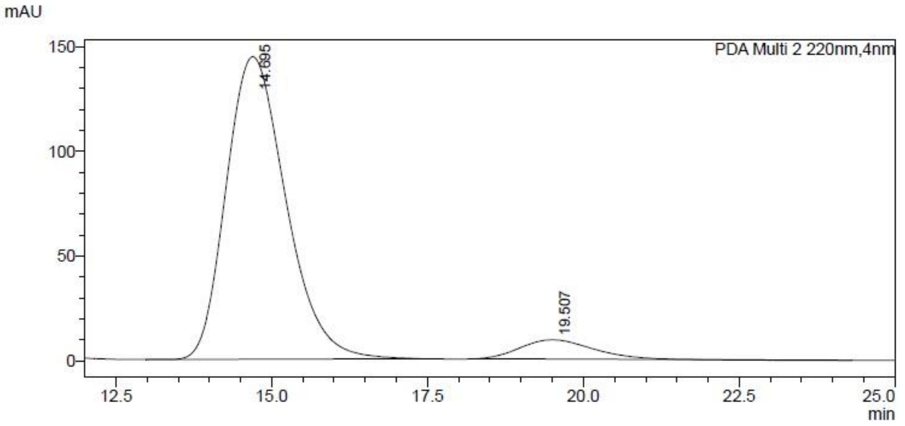

<Peak Table>

PDA Ch2 220nm

Peak#	Ret. Time	Area%
1	14.695	92.752
2	19.507	7.248
Total		100.000

HPLC Data for **30**: Chiralpak AS-H, (80:20 hexane: IPA, flow rate 1.0 mLmin^{-1}, 211 nm, 30 °C) t_R (minor): 22.4 min, t_R (major): 40.7 min, 90:10 er

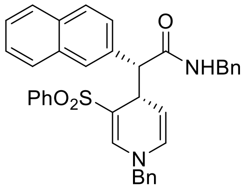

30

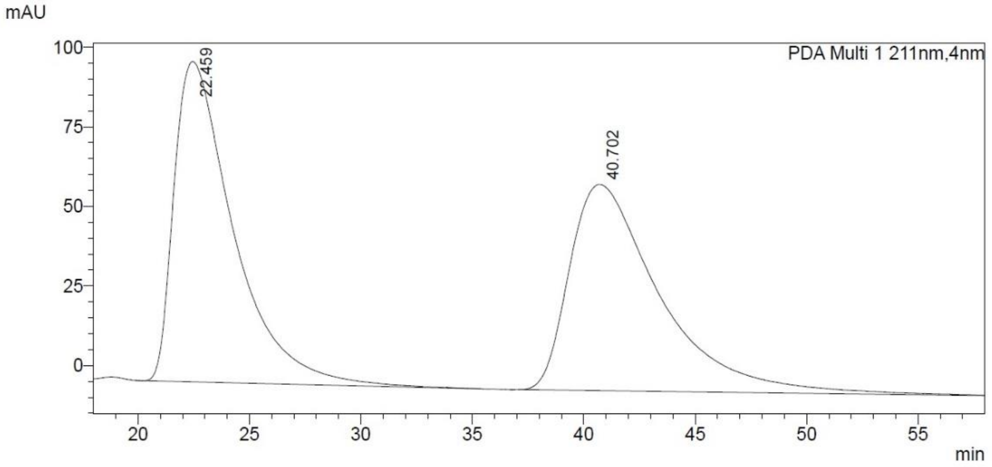

<Peak Table>

PDA Ch1 211nm

Peak#	Ret. Time	Area%
1	22.459	50.519
2	40.702	49.481
Total		100.000

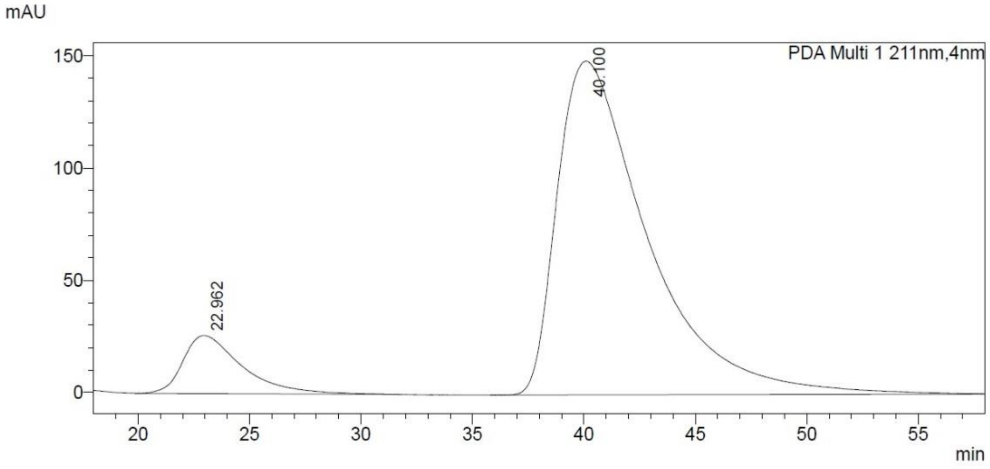

<Peak Table>

PDA Ch1 211nm

Peak#	Ret. Time	Area%
1	22.962	10.109
2	40.100	89.891
Total		100.000

HPLC Data for **31**: Chiralpak IB, (85:15 hexane: IPA, flow rate 1.0 mLmin^{-1}, 220 nm, 30 °C) t_R (major): 17.6 min, t_R (minor): 20.3 min, 98:2 er

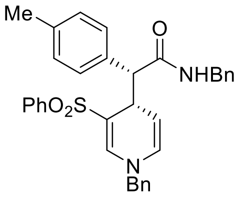

31

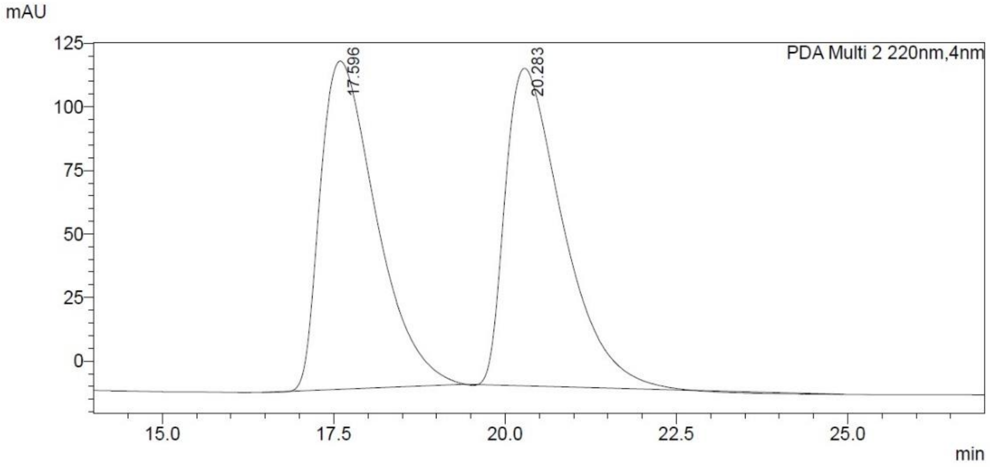

<Peak Table>

PDA Ch2 220nm

Peak#	Ret. Time	Area%
1	17.596	50.113
2	20.283	49.887
Total		100.000

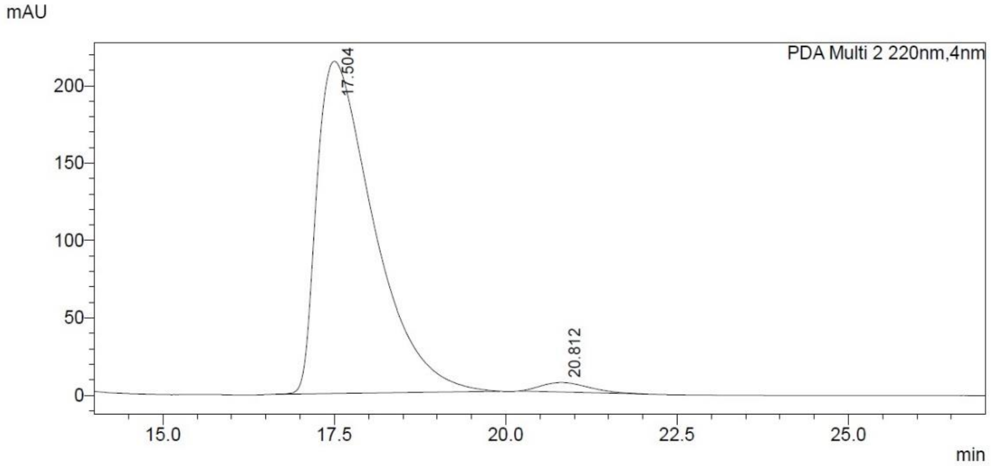

<Peak Table>

PDA Ch2 220nm

Peak#	Ret. Time	Area%
1	17.504	97.653
2	20.812	2.347
Total		100.000

HPLC Data for **32**: Chiralpak IB, (90:10 hexane: IPA, flow rate 0.5 mLmin^{-1}, 211 nm, 30 °C) t$_R$ (major): 79.4 min, t$_R$ (minor): 87.9 min, 98:2 er

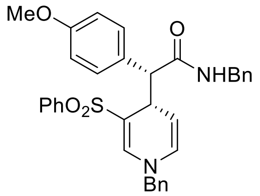

32

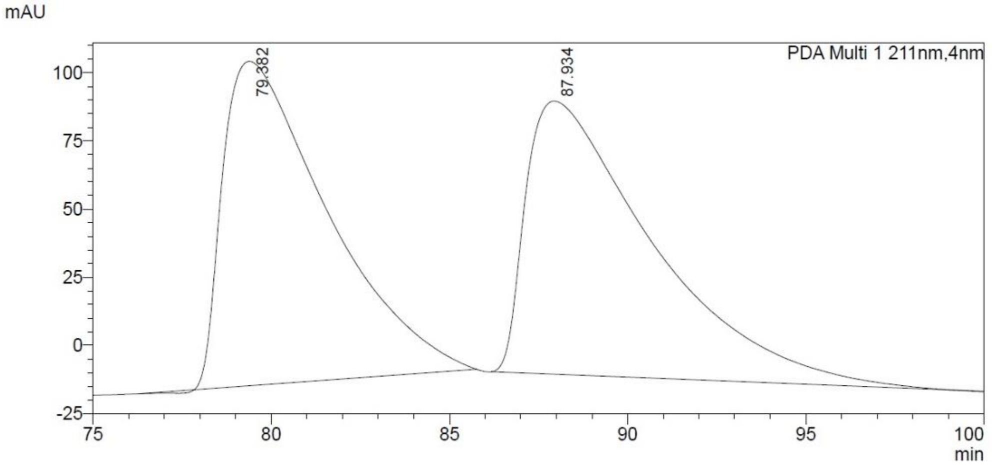

<Peak Table>

PDA Ch1 211nm

Peak#	Ret. Time	Area%
1	79.382	49.518
2	87.934	50.482
Total		100.000

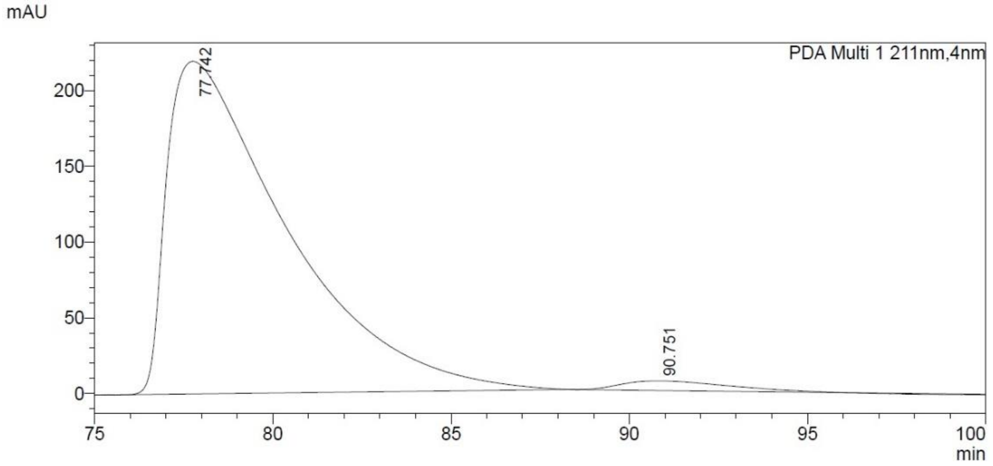

<Peak Table>

PDA Ch1 211nm

Peak#	Ret. Time	Area%
1	77.742	97.661
2	90.751	2.339
Total		100.000

HPLC Data for **33**: Chiralpak IA, (80:20 hexane: IPA, flow rate 1.0 mLmin^{-1}, 211 nm, 30 °C) t_R (major): 31.2 min, t_R (minor): 44.3 min, 96:4 er

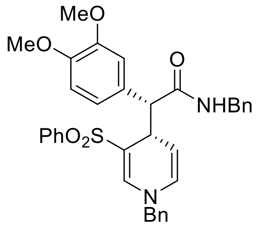

33

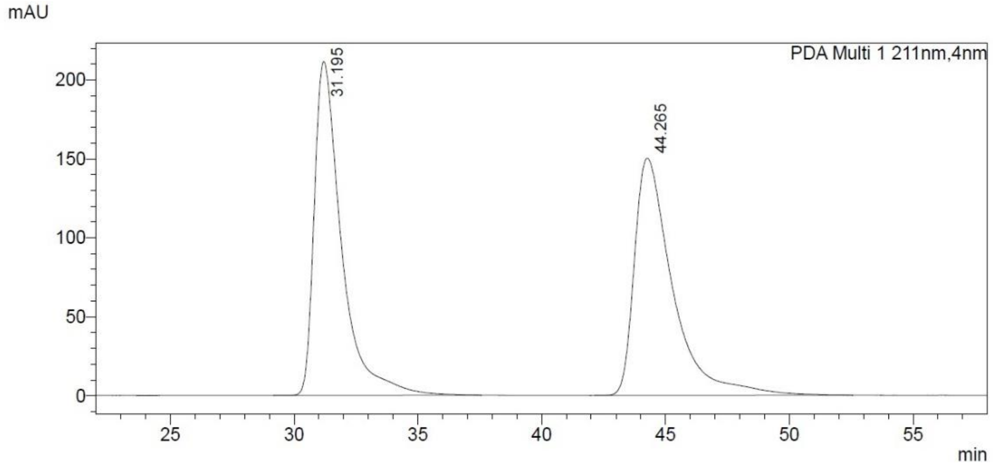

<Peak Table>

PDA Ch1 211nm

Peak#	Ret. Time	Area%
1	31.195	50.064
2	44.265	49.936
Total		100.000

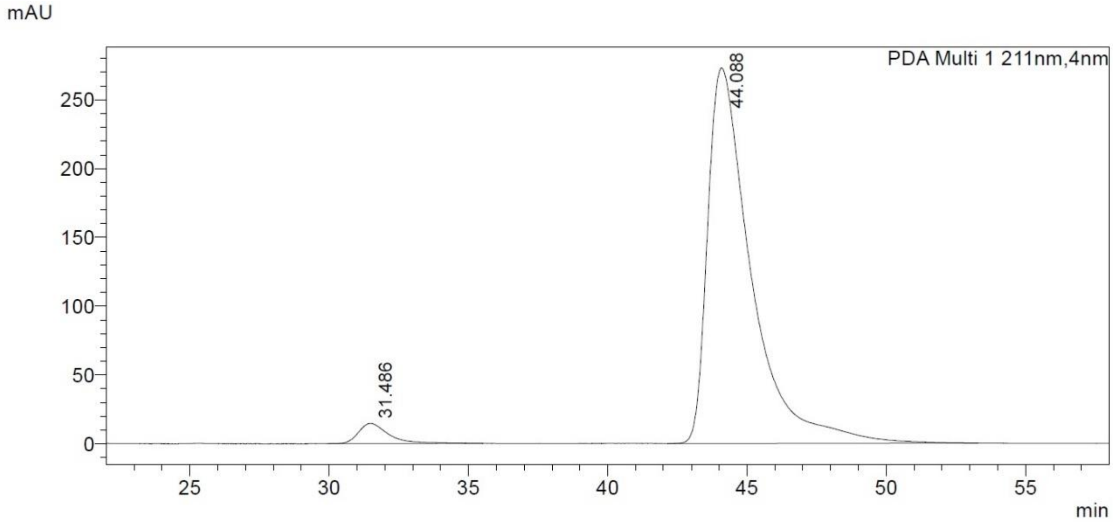

<Peak Table>

PDA Ch1 211nm

Peak#	Ret. Time	Area%
1	31.486	3.637
2	44.088	96.363
Total		100.000

HPLC Data for **34**: Chiralpak IB, (80:20 hexane: IPA, flow rate 1.0 mLmin−1, 211 nm, 30 °C) tR (major): 15.4 min, tR (minor): 19.4 min, 89:11 er

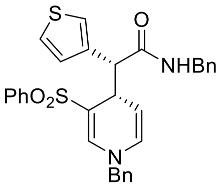

34

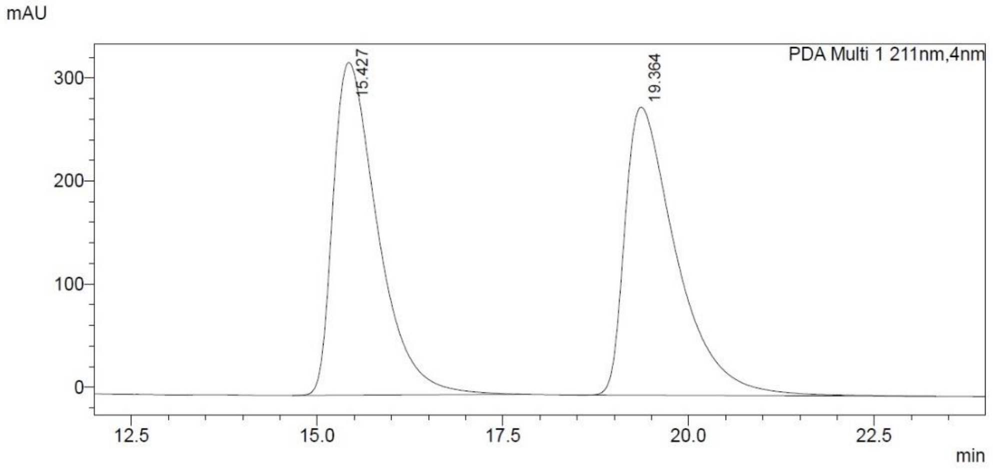

<Peak Table>

PDA Ch1 211nm

Peak#	Ret. Time	Area%
1	15.427	49.912
2	19.364	50.088
Total		100.000

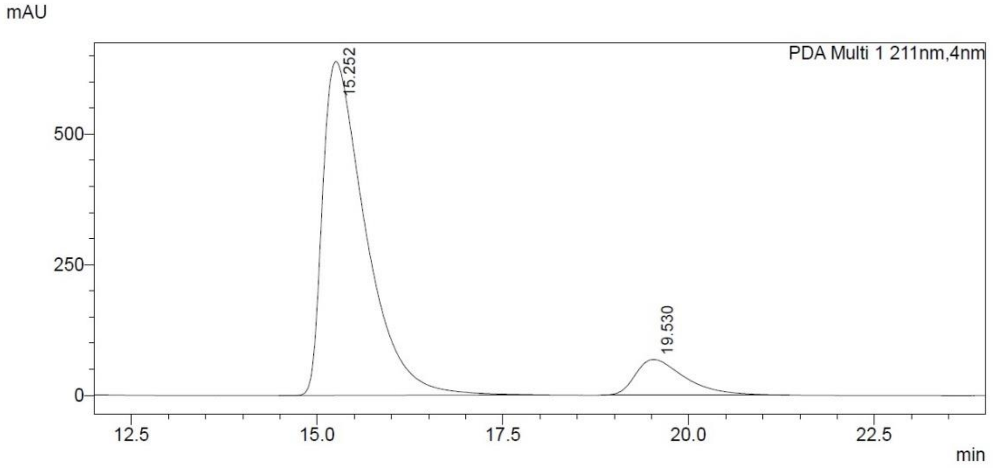

<Peak Table>

PDA Ch1 211nm

Peak#	Ret. Time	Area%
1	15.252	89.078
2	19.530	10.922
Total		100.000

HPLC Data for **35**: Chiralpak AS-H, (85:15 hexane: IPA, flow rate 1.0 mLmin^{-1}, 211 nm, 30 °C) t_R (minor): 13.1 min, t_R (major): 30.0 min, 52:48 er

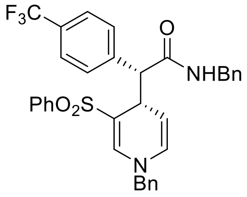

35

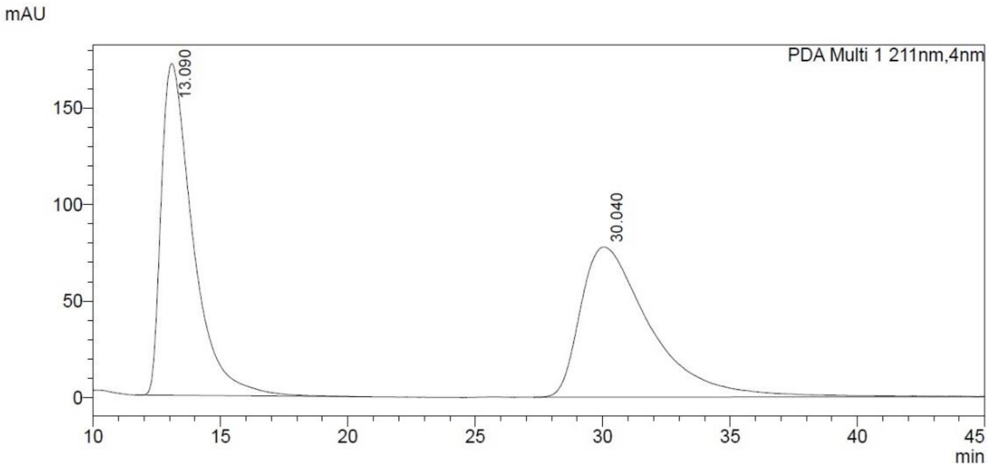

<Peak Table>

PDA Ch1 211nm

Peak#	Ret. Time	Area%
1	13.090	49.768
2	30.040	50.232
Total		100.000

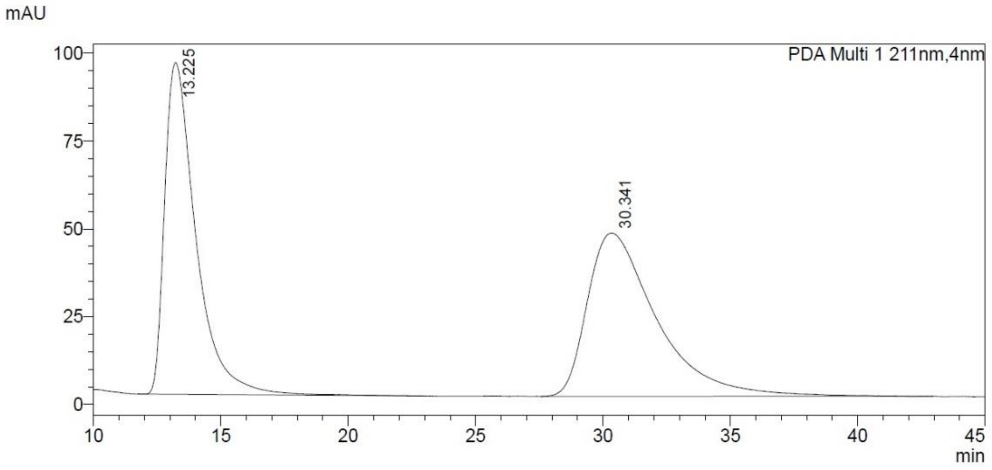

<Peak Table>

PDA Ch1 211nm

Peak#	Ret. Time	Area%
1	13.225	47.851
2	30.341	52.149
Total		100.000

HPLC Data for **36**: Chiralpak AS-H, (80:20 hexane: IPA, flow rate 1.0 mLmin^{-1}, 211 nm, 30 °C) t_R (minor): 11.1 min, t_R (major): 18.2 min, 93:7 er

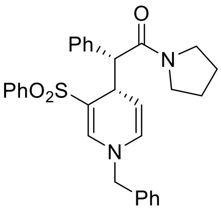

36

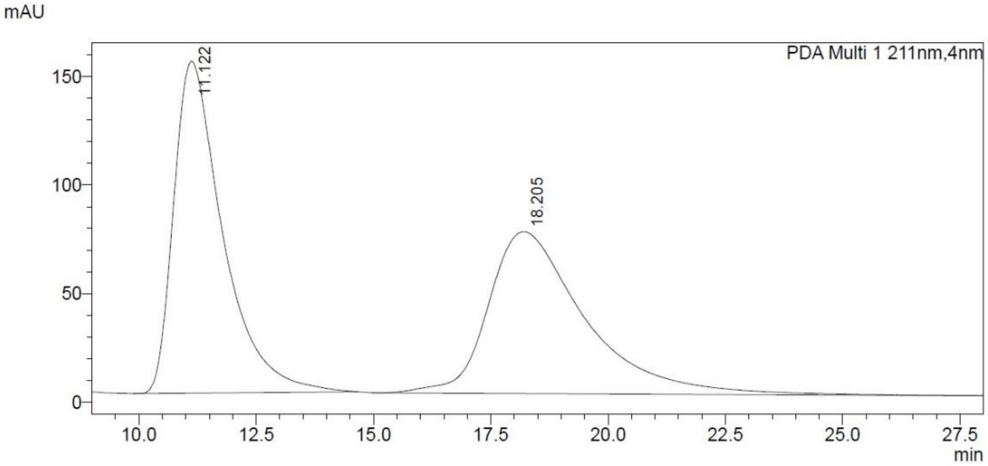

<Peak Table>

PDA Ch1 211nm

Peak#	Ret. Time	Area%
1	11.122	50.993
2	18.205	49.007
Total		100.000

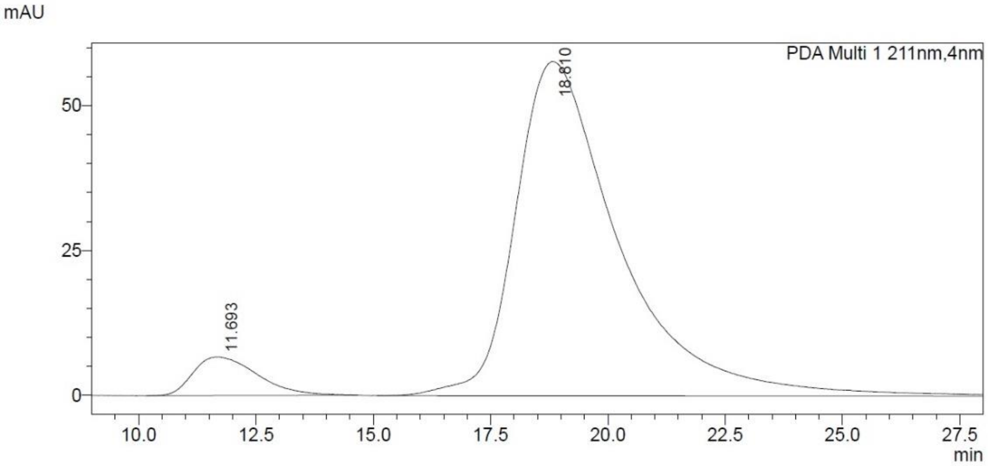

<Peak Table>

PDA Ch1 211nm

Peak#	Ret. Time	Area%
1	11.693	6.658
2	18.810	93.342
Total		100.000

HPLC Data for **37**: Chiralpak IB, (80:20 hexane: IPA, flow rate 1.0 $mLmin^{-1}$, 211 nm, 30 °C) t_R (major): 14.0 min, t_R (major): 19.2 min, 94:6 er

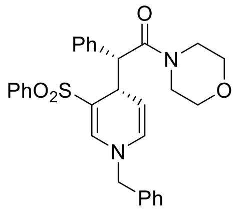

37

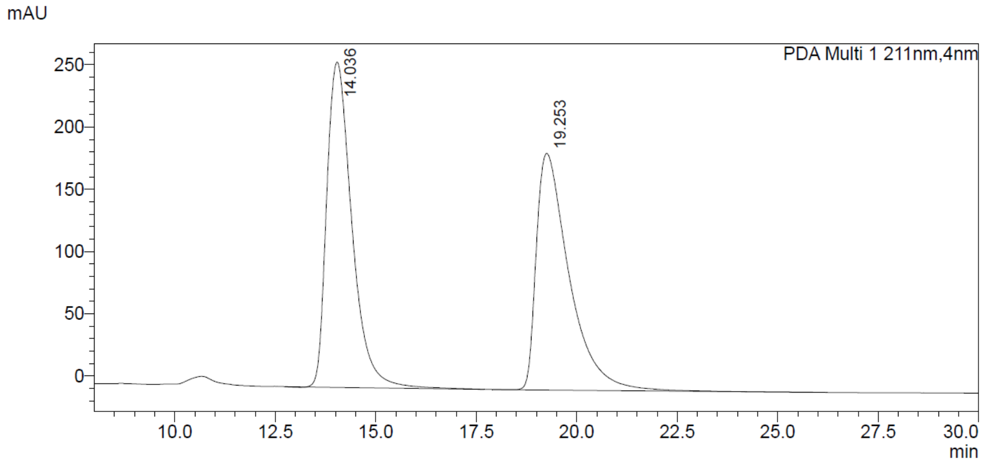

<Peak Table>

PDA Ch1 211nm

Peak#	Ret. Time	Area%
1	14.036	51.302
2	19.253	48.698
Total		100.000

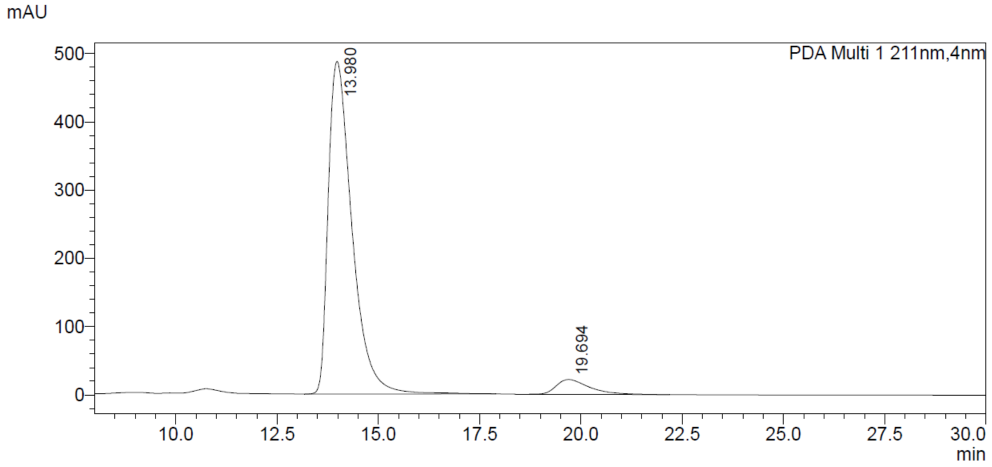

<Peak Table>

PDA Ch1 211nm

Peak#	Ret. Time	Area%
1	13.980	94.133
2	19.694	5.867
Total		100.000

HPLC Data for **38**: Chiralpak IB, (80:20 hexane: IPA, flow rate 1.0 mLmin^{-1}, 220 nm, 30 °C) t_R (major): 12.1 min, t_R (major): 17.7 min, 93:7 er

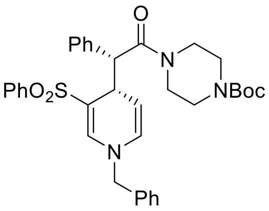

38

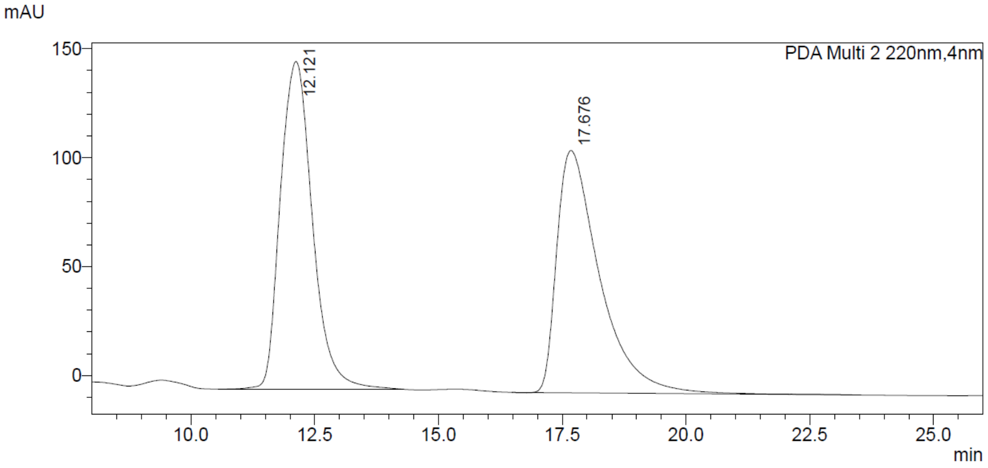

<Peak Table>

PDA Ch2 220nm

Peak#	Ret. Time	Area%
1	12.121	50.277
2	17.676	49.723
Total		100.000

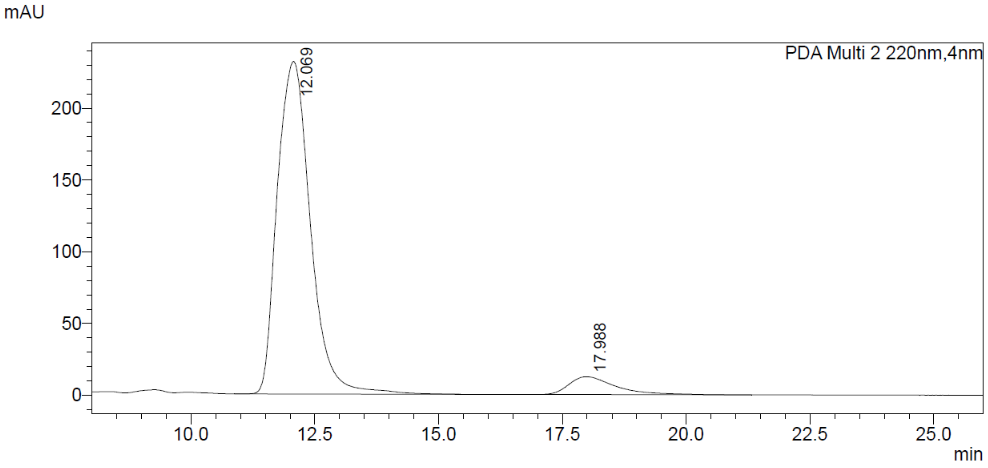

<Peak Table>

PDA Ch2 220nm

Peak#	Ret. Time	Area%
1	12.069	93.022
2	17.988	6.978
Total		100.000
